# Rational Design of Metal Oxide Nanostructures via Dopant Control: A Case Study in Photoelectrochemical Performance

**DOI:** 10.1021/acsami.5c16912

**Published:** 2025-11-13

**Authors:** Mariana A. Dotta, Fabio A. Pires, Karen C. Bedin, Ingrid Rodríguez-Gutiérrez, Francine Coa, Heloisa H. P. Silva, Gabriel R. Schleder, Carolina P. Torres, Fabiano E. Montoro, Diego S. T. Martinez, Jefferson Bettini, Edson R. Leite, Renato V. Gonçalves, Flavio L. Souza

**Affiliations:** † Brazilian Nanotechnology National Laboratory (LNNano), 215006 Brazilian Center for Research in Energy and Materials (CNPEM), Campinas, São Paulo, CEP 13083-100, Brazil; ‡ Institute of Chemistry (IQ), State University of Campinas (UNICAMP), Campinas, São Paulo, CEP 13083-970, Brazil; § School of Technology (FT), State University of Campinas (UNICAMP), Limeira, São Paulo, CEP 13484-332, Brazil; ∥ São Carlos Institute of Physics (IFSC), University of São Paulo (USP), São Carlos, São Paulo, CEP 13566-590, Brazil; ⊥ Humanities and Nature Science Center (CCNH), 74362 Federal University of ABC (UFABC), Santo André, São Paulo, CEP 09210-580, Brazil

**Keywords:** metal oxides, nanostructures, doping, hematite, energy conversion

## Abstract

On-demand material design is reshaping the synthesis landscape of multifunctional systems. In this scenario, the ability to tailor functional properties through deliberate control of composition and structure marks a significant advancement in materials science. Herein, a polymeric precursor solution method that enables spatially resolved dopant incorporation into oxide nanostructures is employed to design CuO, CeO_2_, and α-Fe_2_O_3_ multifunctional systems. X-ray diffraction confirms the obtention of high-purity single-phase oxides. Further investigations using hematite (α-Fe_2_O_3_) as a model system demonstrate that lattice doping yields nanostructures with at least 2-fold increase in thickness and enhanced porosity, while modulating intragrain conductivity, as revealed by intensity-modulated photocurrent spectroscopy. Interfacial dopant segregation leads to smaller grains (from 28 to 19 nm), more compact morphologies, and lower energy barriers at grain boundaries, enhancing intergrain charge transport. Density functional theory supports this behavior, showing interfacial barrier reduction from 1.88 eV (pristine) to 1.31 eV (doped). Targeting photoelectrochemical performance, the combined use of lattice and interfacial doping induces synergistic changes in porosity, grain size, film thickness, and both intra- and intergrain conductivity. Through spatial control of dopant distribution, the PPS approach offers a versatile platform for the rational design of tunable metal oxides for energy, catalysis, and beyond.

## Graphical abstract

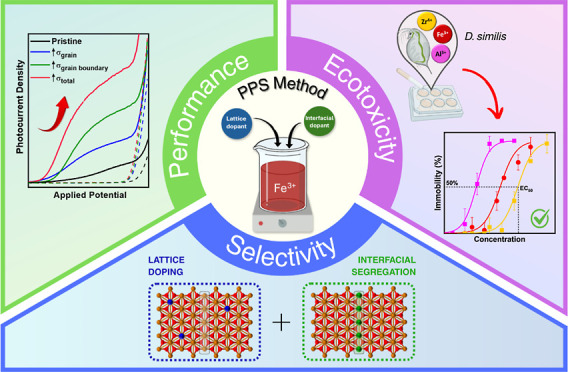

## Introduction

Polycrystalline metal oxides based on earth-abundant elements have emerged as promising candidates for next-generation functional materials,^1,2^ offering a combination of chemical stability, structural tunability, and environmental compatibility.^3,4^ Their wide-ranging applicability spans bioscience,^5^ nanomedicine,^6^ anticorrosion coatings,^7^ gas sensing,^8^ and energy conversion technologies,^9^ among others. Many of these oxides play a critical role in advancing sustainable solutions, particularly in the context of the global energy transition.^10^ Their ability to have structural and electronic properties tailored to specific functions makes them attractive for multifunctional device integration.^11,12^

However, despite these advantages, the polycrystalline nature of such materials introduces inherent limitations. Grain boundaries, essential features of polycrystalline structures, often serve as sources of defects, local strain, and compositional inhomogeneity.^13^ These irregularities can disrupt charge transport, reduce ionic conductivity, and hinder overall material performance.^14^ Additionally, several oxides—such as BiVO_4_, TiO_2_, CuO, and α-Fe_2_O_3_—exhibit low charge carrier mobility derived from strong electron–lattice interactions,^15–18^ which are intrinsically associated with the formation of small polarons within their crystal structures. This phenomenon introduces further complexity to their charge dynamics, as the restricted electron mobility impairs efficient transport and interfacial charge transfer. For instance, electronic issues such as trap states, interfacial charge recombination, and low carrier mobility are especially problematic for applications in photovoltaics, memristive devices, and photoelectrochemical systems.^19^ As a result, developing strategies to overcome these structural and electronic bottlenecks remains crucial for fully realizing their potential in advanced technologies.^20–23^

Over the years, material synthesis has typically followed the approach of identifying a target application and adapting synthesis methods to suit that specific need. While this strategy has led to the development of numerous methodologies,^24^ it lacks the ability to keep pace with the speed and complexity of modern technological demands. Increasingly, the field is shifting toward an “on-demand” paradigm—one in which materials are not just optimized for a known purpose, but tailored from the outset to satisfy diverse property requirements.^25^ This shift emphasizes inherently problem-driven synthesis strategies that enable the design and production of materials in advance of a specific application, guided by desired properties and performance targets. Recent advances in automation and computational design are accelerating this process.^26^ However, their full potential depends on the development of chemical routes that offer precise control over composition, crystallinity, and nanostructure to reliably achieve the desired material properties.

In this context, the polymeric precursor solution (PPS) method came forth as a promising alternative that recently hinted its potential toward selective placement of modifiers targeting to overcome the intrinsic limitations of hematite in photoelectrochemical (PEC) systems.^27–29^ Although widely studied as a model oxide in PEC devices, hematite—commonly known as rust—still requires careful tailoring to unlock its full potential.^30^ Following the PPS approach, lattice doping was employed according to traditional sol–gel strategies,^31^ with modifiers introduced to interstitially or substitutionally occupy positions within the hematite crystal structure. This doping aimed to reduce polaron formation and enhance charge transport by disrupting electron–lattice interactions through local symmetry breaking around Fe sites.^32^ While effective in generating mesoporous structures with enhanced grain-level conductivity, lattice doping alone proved insufficient to overcome interfacial transport limitations. To address this, a second element was introduced to segregate at the crystal surface, forming dopant-rich regions at grain boundaries. This interfacial modification reduced grain-to-grain energy barriers and improved electron mobility across the film.^33^ An additional benefit was the suppression of grain growth during annealing, resulting in finer grains and improved film packing. When both elements were introduced simultaneously into the precursor solution, the resulting hematite photoanodes demonstrated state-of-the-art performance, underscoring the effectiveness of this dual-modification strategy.^27,28^

Although the method was initially developed targeting a specific application (PEC systems), its ability to precisely and selectively control dopant incorporation in oxide nanostructures offers a flexible platform for tuning material properties, enabling on-demand design of metal oxide semiconductors. Regardless of the promising results pointing out an effective and robust strategy to address the main drawbacks shared by a variety of polycrystalline systems, however, key questions remain elusive regarding the chemical mechanism underlying the observed selective positioning of modifiers. Is this behavior a direct consequence of the PPS methodology, or is it primarily governed by the intrinsic properties of the incorporated elements? Furthermore, if lattice dopants exceed their solubility limit, will they migrate toward grain boundaries similarly to intentionally segregated modifiers? After some works that validated the strategy, expanding the procedure for different metal oxide systems and answering these questions became critical for improving control over dopant distribution and maximizing the method’s intended applicability.

Therefore, this work designs multifunctional metal oxides such as CuO, CeO_2_, and α-Fe_2_O_3_ to evaluate the versatility of the PPS method in selectively placing single or multiple modifiers within oxide-based nanostructures. Hematite (α-Fe_2_O_3_) was chosen as a model system for further investigation due to its well-known electronic complexity, particularly its strong polaronic effects.^18^ Hematite photoanodes modified with Al^3+^ (57 pm), Ga^3+^ (62 pm), Y^3+^ (106 pm), and La^3+^ (122 pm) were synthesized to investigate how modifiers with distinct ionic radii and electronegativities influence their final position within the structure and impact material performance. This selection enables a detailed assessment of the PPS method’s ability to guide modifier distribution through careful chemical design. The impact of these modifiers was systematically evaluated by correlating structural, morphological, optical, and electronic characteristics with photoelectrochemical performance. Notably, PEC measurements allowed us to distinguish how modifiers influence the material depending on whether they are incorporated into the lattice, segregated at the surface, or localized at grain boundaries. It is worth noting that this work does not aim to identify the optimal dopant for hematite, but rather to highlight a chemically robust and tunable synthesis route for engineering metal oxide nanostructures. The results presented here demonstrate the power of the PPS method as a platform for designing oxide-based materials with spatially controlled dopant distributions, paving the way for customizable functionalities in energy, catalysis, and beyond.

## Results and Discussion

Figure 1 illustrates how the polymeric precursor solution (PPS) method enables on-demand synthesis of polycrystalline metal oxide semiconductors by introducing cationic modifiers at distinct stages of the synthesis process (Step 1 or Step 2). This strategy enables the targeted incorporation of modifiers at specific crystal positions, addressing bulk, crystal surface, and interfacial bottlenecks for the fabrication of materials with controlled characteristics.

**Figure 1:**
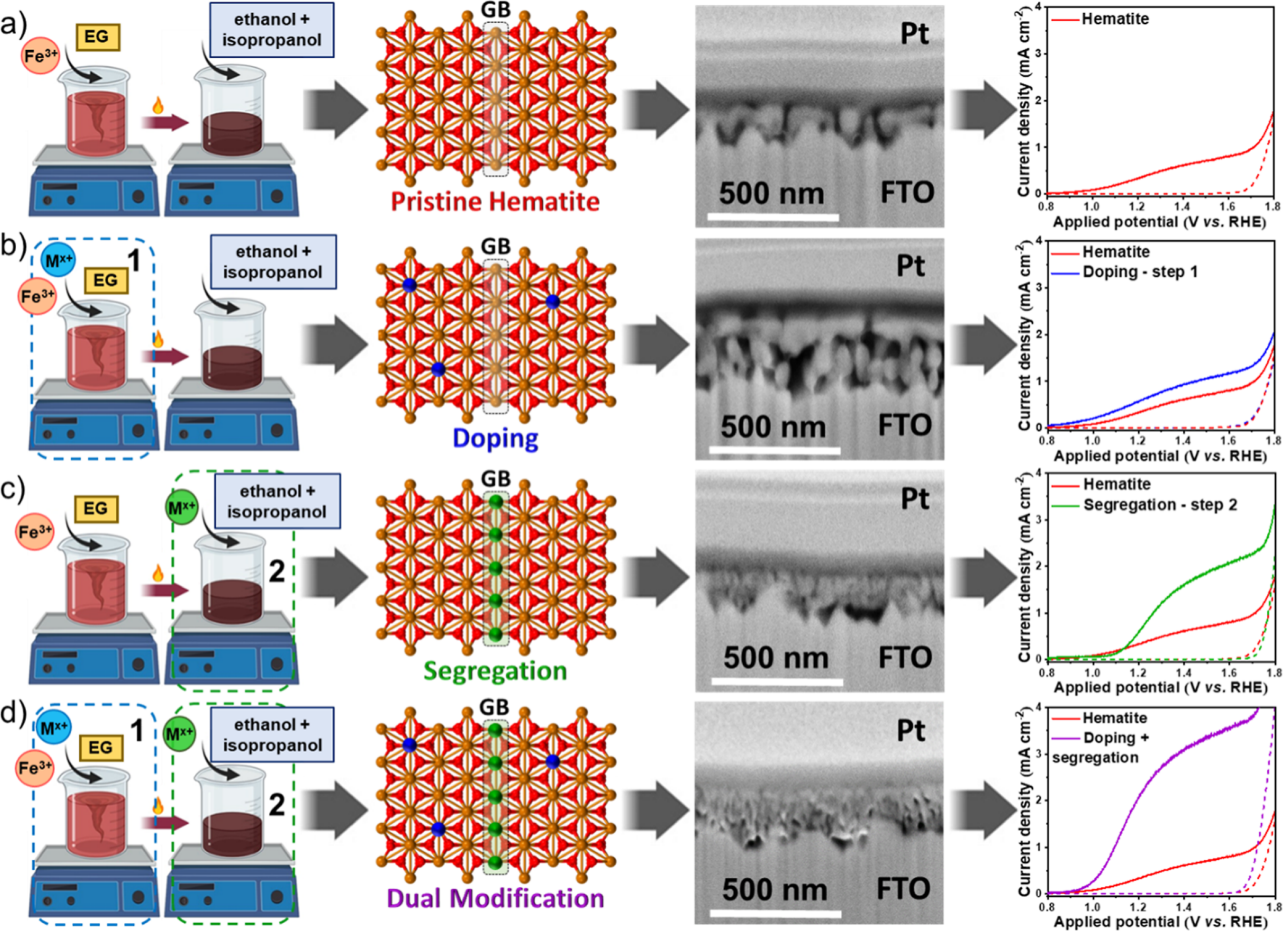
Scheme detailing the different steps of the polymeric precursor solution (PPS) method for selective modifier placement. (a) Exemplary synthesis of pristine hematite yields a nanostructure with multiple grain boundaries (GB), limiting solar water splitting performance. (b) Step 1 modification (lattice doping) yields porous, elongated grains with enhanced intragrain charge transport. (c) Modifications at Step 2 (interfacial segregation) result in nanostructures with smaller, densely packed grains and reduced GB energy barriers. (d) Dual modification combines these features synergistically, concurrently addressing intragrain and intergrain charge transport limitations.

A pristine metal oxide nanostructure (Figure 1a) is produced by mixing a metallic (e. g., Cu^2+^, Fe^3+^, Ce^4+^) salt with a carboxylic acid (citric acid) and a polyalcohol (ethylene glycol) in aqueous media and bringing the solution to polymerize, forming a polyester that can be sprayed, spin coated, or dip coated onto a conductive substrate, or even calcined to produce powders. This solution is kept under heating for several hours until its water content is reduced by 50%, aiming to enhance the polymerization yield (see ‘The PPS method’ section in the Supporting Information file). The obtained gel is then diluted with an ethanol/isopropanol mixture, which increases the solution’s polarity and induces conformational changes in the polymeric chains,^34^ promoting improved grain packing onto the substrate surface during annealing in thin films fabrication.^35^ This leads to a commonly observed worm-like grain morphology with multiple interfaces that uniformly cover even rough surfaces such as fluorine-doped tin oxide (FTO), as seen in the cross-sectional image of Figure 1a. The multi-interfaced nature of the pristine structures produced by the method typically yields materials with the presence of highly energetic interfaces between grains (i.e., grain boundaries–GB), which results in overall low conductivity.^33,34^ For iron oxide (hematite phase), for example, this translates into low photocurrent densities and, for copper oxide, accelerated photocorrosion at neutral pHs when applied for solar water splitting.

The introduction of lattice dopants is achieved by individually mixing selected metallic salts (solutes) with the host metal and citric acid in aqueous media before bringing the solution to polymerize (Step 1 of synthesis, Figure 1b). As, in this case, the host metal and the dopants share a common chemical environment during polymerization, modifying cations are incorporated into the polymeric network.^31^ This is ensured by mild temperature conditions, preventing oligomer decomposition and sustaining controlled water evaporation kinetics. Differential scanning calorimetry (DSC) analyses (Figure S1) showed different polymer crystallinities when dopants were incorporated during Step 1 of the PPS synthesis, corroborating the dopant integration into the polymeric structure. This step is done to favor dopant positioning within the crystal lattice during annealing, delivering metal oxides with elongated grains (changes in anisotropy), accompanied by increased porosity and film thickness. For materials like hematite, which have carrier mobility constrained by the polaronic domain, this modification leads to improved intragrain conductivity through the mitigation of small-polaron effects, translated as an enhancement in its photoelectrochemical (PEC) behavior (Figure 1b).

The synthesis stage referred as Step 2 (Figure 1c) promotes interfacial segregation of chemical modifiers by incorporating metallic cations into the solution after the thermoplastic gel is formed. This enables modifiers to percolate polymeric chains without being integrated into their structure, resulting in a material whose crystallinity remains comparable to the undoped polymer (Figure S1). As, in this case, modifiers do not share the same coordination environment as the host metal, their accumulation at crystal surfaces is favored during thermal treatments. This strategy yields nanostructured films with smaller, densely packed grains and improved substrate contact (see cross-sectional image of Figure 1c). For PEC applications, charge separation efficiency is usually greatly increased by this strategy, as intergrain charge transport is improved. When both strategies (lattice doping and interfacial segregation) are simultaneously employed (Figure 1d), a carefully tailored final material combines the features of individual modifications (increased porosity and more elongated grains that are better packed onto the substrate), tackling the main conductivity bottlenecks of multi-interfaced metal oxides.

We have shown the successful production of CuO, α-Fe_2_O_3_, and CeO_2_ thin films following the PPS protocol, with only pure phases detected when dopants were incorporated in any of the synthesis steps (see Figure S2). Iron was highlighted as the host metal in Figure 1 because hematite was chosen for the case study reported here, given that this iron oxide phase is commonly referred to as a model system for (photo)­(electro)­catalysis due to its well-documented intrinsic structural and electronic bottlenecks.^36^ A photoelectrochemical framework was also selected in this work to experimentally probe the modifications introduced by the PPS method. Naming a given dopant by X, from now on all samples in which modification took place during Step 1 of the synthesis will be represented as XH (H denoting Hematite). Accordingly, samples in which modification took place during Step 2 of the synthesis will be represented as HX. The following sections demonstrate the ability of the PPS method to selectively induce lattice doping and interfacial segregation using distinct trivalent cations, systematically comparing the fingerprints of each modification strategy to validate the methodology’s effectiveness.

### Lattice Doping

To initially highlight the ability of the PPS method in promoting lattice doping and to explore the role of modifiers in oxide semiconductors, distinct elements with the same oxidation state as iron (+3) were employed to dope α-Fe_2_O_3_. Y^3+^ (YH), La^3+^ (LaH), Al^3+^, and Ga^3+^ (GaH) salts (listed in Table S1) were incorporated into hematite at concentrations of 1.0% (molar ratio to Fe^3+^), according to what is described in Figure 1b. Recent computational studies revealed that the solubility limit of hematite lies within the dopant concentration of <0.1% for some elements of groups IV and XIV.^37^ Our previous experimental works further showed that this limit may be slightly higher for elements such as aluminum, gallium and yttrium, in which the optimal doping concentration was found to be 0.5%.^27–29^ The favored enthalpy of mixing modifier cations with iron during Step 1 of the PPS synthesis (Figure 1b) is expected to favor lattice doping, but adding 1.0% of dopants stresses the solubility limit imposed by the doping bottleneck in hematite (0.1%–0.5%), possibly leading to lattice strain during phase formation. This strain might force the excess of impurities to be exuded from the crystal, resulting in their accumulation at high energy surfaces during coarsening. Therefore, elements were incorporated in concentrations exceeding hematite’s solubility limit to determine whether lattice dopants partially migrate to the crystal surface, and to assess if the properties of resulting nanostructures are comparable to those in which modifiers are intentionally designed to segregate at grain boundaries.

In addition, trivalent elements were selected to minimize variations of charge donor density numbers (N_D_), as they possess the same oxidation state as iron in α-Fe_2_O_3_. This way, it is possible to study the influence of lattice dopants in enhancing intragrain conductivity by exclusively improving electronic mobility.

The incorporation of modifiers into the crystal lattice was first evaluated through X-ray diffraction (XRD) analysis. Diffractograms obtained for pristine hematite and lattice doped photoanodes are displayed in Figure S3. Diffraction peaks were assigned to α-Fe_2_O_3_ phase (rhombohedral structure, space group *R*3̅*c*, JCPDS 33-0664) and SnO_2_ (cassiterite phase, JCPDS 41-1445) from the commercial transparent oxide conductive layer (F/SnO_2_), with no detection of secondary phases associated with modifiers. The cell parameters (*a*, *c*, and volume) were calculated according to the hexagonal structure formula and are shown in Table S2. For pristine material, values of *a* = 4.93 Å, *c* = 13.47 Å, and V = 284 Å^3^ were estimated. YH, LaH, and GaH photoanodes presented *c* lengths of 13.49 Å, 13.46 Å, and 13.48 Å, respectively, slightly deviating from the Hem unit cell. YH and LaH also showed a small increase in a length, reaching 4.94 Å, while Y^3+^ incorporation resulted in an increase in the cell volume to 285 Å^3^. The slight variations observed in lattice parameters can be an indicative of elemental doping. The only exception was AlH, which presented the same *a*, *c*, and V values as pristine hematite. Lattice strain is expected to be lower upon Al^3+^ modification, as aluminum forms the same crystalline structure of hematite (corundum) and presents the lowest ionic radii among the studied modifiers. To further assess the impact of incorporated elements on the structure of photoanodes, the texturization coefficient and crystallite size of synthesized samples were analyzed.

Pristine hematite is textured along the (1 1 0) direction, which is induced by the FTO commercial substrate.^38^ This is translated by the Lotgering factor (F)^39^ of 0.71 for Hem in the (1 1 0) plane, as displayed in Figure 2a and Table S2. The incorporation of Y^3+^, La^3+^, Al^3+^, and Ga^3+^ clearly influenced the texturization of the films, as indicated by the decrease in the F factor for the (1 1 0) plane in YH (F = 0.49), LaH (F = 0.11), AlH (F = 0.49), and GaH (F = 0.69). This change is accompanied by an enhancement in the signals corresponding to the (1 0 4) and (3 0 0) planes in the diffractograms of lattice doped samples, indicating that modifier incorporation boosts film growth in orthogonal directions to (1 1 0). As a result, it is possible to notice changes in the crystallite sizes of doped photoanodes compared to pristine hematite in the (1 1 0), (1 0 4), and (3 0 0) planes (Table S2). This arises due to the reorientation of grains in doped samples, which alters their growth across different planes, potentially resulting in grain elongation. In addition, among modifiers, it is particularly evident that lanthanum induced film texturization along the (1 0 4) plane, which is assigned to the higher lattice strain provoked by La^3+^ in hematite’s structure due to its larger ionic radius (122 pm) in comparison to iron (64 pm).^29,40^

**Figure 2:**
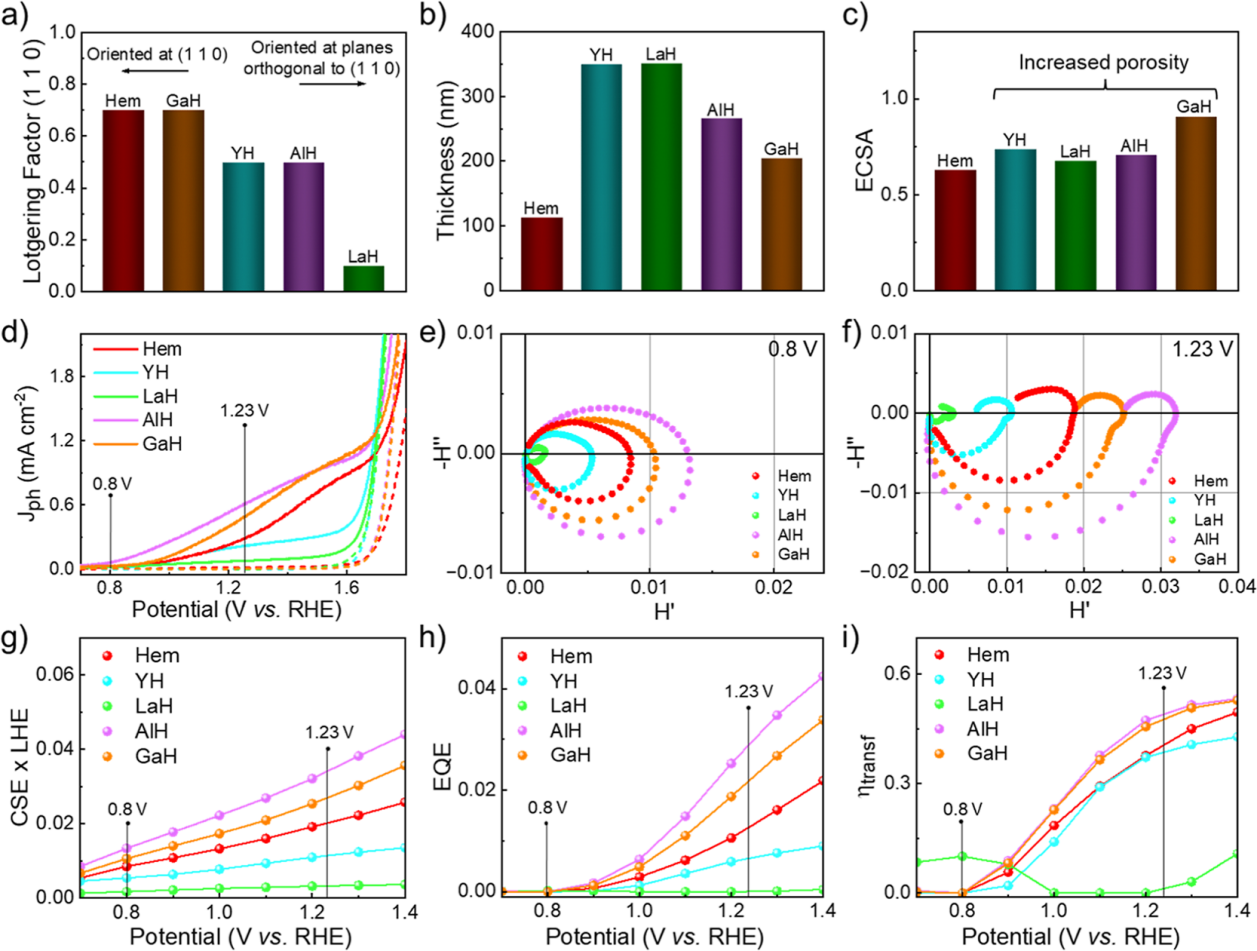
(a) Bar chart displaying the Lotgering factor calculated along the (1 1 0) plane for pristine (Hem) and lattice-doped (Step 1) hematite photoanodes. (b) Estimated thicknesses of Hem, YH, LaH, AlH, and GaH photoanodes, determined from FIB-SEM analysis. (c) Electrochemically active surface area (ECSA) estimated from cyclic voltammetry experiments for pristine (Hem) and lattice-doped (Step 1) hematite photoanodes. (d) Photocurrent density (*j*–*V*) profiles of Hem, YH, LaH, AlH, and GaH photoanodes under simulated sunlight (solid lines) and dark conditions (dashed lines). (e) Nyquist plots obtained from IMPS analysis at 0.8 *V*_RHE_ for Hem, YH, LaH, AlH, and GaH photoanodes. (f) Nyquist plots obtained from IMPS analysis at 1.23 *V*_RHE_ for Hem, YH, LaH, AlH, and GaH photoanodes. (g) Charge separation versus light harvesting (CSE × LHE), (h) external quantum (EQE), and (i) charge transfer (η_transf_) efficiencies derived from IMPS analysis across the 0.7–1.4 *V*_RHE_ potential range.

Focused ion beam scanning electron microscopy (FIB-SEM) analysis (Figure S4) revealed an increase in film thickness for all lattice-doped samples in comparison to pristine hematite (114 ± 6 nm), reaching 350 ± 16 nm for YH, 351 ± 36 nm for LaH, 266 ± 23 nm for AlH, and 205 ± 28 nm for GaH, as shown in the bar chart of Figure 2b. Higher thickness results in increased porosity, considering the amount of material deposited is constant in the PPS method. To investigate this aspect, the electrochemically active surface area (ECSA) of pristine and lattice doped photoanodes was estimated through cyclic voltammetry (Figures S5 and S6), as higher porosity is expected to expose a greater surface area to the electrolyte. Figure 2c and Table S3 show the calculated ECSA values for Hem (0.63), YH (0.74), LaH (0.68), AlH (0.71), and GaH (0.91). A clear increase in ECSA is noted upon doping, corroborating the more porous structures observed in FIB-SEM cross-sectional images.

Conversely, atomic force microscopy (AFM) analysis (Figure S7) revealed a reduction of surface roughness upon lattice dopant incorporation (Table S4), with mean roughness values of 45.1 nm for Hem, 18.2 nm for YH, 17.8 nm for LaH, 24.8 nm for AlH, and 32.2 nm for GaH. Given the inherently rough texture of the FTO substrate, the observed reduction in surface roughness from pristine to lattice-doped photoanodes suggests that the incorporation of modifiers alters the polymeric configuration, enabling polymer chains to more effectively fill the FTO valleys and form a smoother, uniform layer. Additionally, topographical images show the maintenance of nanograin size for pristine and lattice doped photoanodes, as previously observed through FIB-SEM analysis.

Adding modifying elements to dope the crystal lattice also influenced the optical properties of the photoanodes. As shown in Figure S8a, a considerable increase in the absorption properties was caused when lattice doping was applied, resulting in higher J_abs_ values (maximum generated photocurrent as a function of the light absorption of the materials, Figure S8b). However, the estimated optical bandgap values of doped materials (Figure S8c) show negligible variations in comparison to Hem, indicating the observed effect is only due to light absorption increase, consistent with different thicknesses estimated by cross-section FIB-SEM images.

To further assess the impact of modifiers on the oxide’s structure, X-ray photoelectron spectroscopy (XPS) measurements were performed. Figure S9 provides a detailed insight into the chemical state of iron in both pristine and lattice doped photoanodes. The Fe 2p core-level spectra indicate that Fe is present in the Fe^3+^ oxidation state across all samples, with characteristic peaks at binding energies around 710–712 eV (Fe 2p_3/2_) and 723–725 eV (Fe 2p_1/2_).^41^ A broad satellite peak in the 718–720 eV range further confirms the presence of Fe^3+^.^42^

Figure S10 displays the high high-resolution XPS spectra of Y 3d, La 3d, Al 2p and Ga 2p from YH, LaH, AlH, and GaH samples, respectively. Measured binding energies indicate that all modifiers are in their trivalent states.^43–46^ Given that XPS is sensitive only to the top few nanometers of the material, the detection of yttrium (Figure S10a), lanthanum (Figure S10b), aluminum (Figure S10c), and gallium (Figure S10d) implies that they are concentrated at or near the outermost surface layers. Considering the applied modifier concentration of 1.0%, which is ten times higher than the dopant solubility limit of hematite, it is feasible to consider that impurity excesses have been exuded to the surface of the photoanodes. This is a hint that may distinguish the partial migration of modifiers to the crystal surface during Step 1 of synthesis from their intentional placement at interfaces in Step 2. Notwithstanding, despite the observed diffusion of lattice dopants, the predominant behavior of samples modified during Step 1 of the PPS protocol was the increase in porosity and elongation of grains.

To unveil the impacts of lattice doping on the charge dynamics of hematite, photoelectrochemical experiments were conducted. Figure 2d depicts the *j*–*V* curves obtained for Hem, YH, LaH, AlH, and GaH. Pristine hematite exhibited a photocurrent density of 0.26 mA cm^–2^ at 1.23 *V*_RHE_. The lattice incorporation of aluminum (*J*_ph_ = 0.57 mA cm^–2^ at 1.23 *V*_RHE_) and gallium (*J*_ph_ = 0.45 mA cm^–2^ at 1.23 *V*_RHE_) was beneficial for photoelectrochemical applications, promoting a mild and constant improvement in the photogenerated current density throughout the analyzed potential range. This can be associated with the previously reported increase in the electrochemically active surface area of doped samples, indicating the presence of more active sites to promote the water oxidation reaction. AlH also promoted a cathodic shift in the onset potential of the oxygen evolution reaction.

Conversely, yttrium limited hematite’s PEC performance (*J*_ph_ at 1.23 *V*_RHE_ = 0.21 mA cm^–2^), while lanthanum (*J*_ph_ at 1.23 *V*_RHE_ = 0.07 mA cm^–2^) dropped hematite’s photoresponse to nearly zero. This is most likely given by the texturization of LaH in the (1 0 4) direction, which would lower the photoanode conductivity, as hematite electron hopping is favored within iron layers (i.e., in the (1 1 0) direction) and is spin-forbidden in planes orthogonal to the (0 0 1) basal plane.^47^ In addition, Y^3+^ and La^3+^ dopant clusters can take place at the structure,^37^ further constraining charge transfer processes. This shows that, despite the PPS protocol selectivity in promoting lattice doping, rigorous criteria must be applied during dopant selection to achieve enhanced performance in desired applications.^29^

Figure 2e,f show the intensity modulated photocurrent spectroscopy (IMPS) Nyquist plots obtained at 0.8 *V*_RHE_ and 1.23 *V*_RHE_ under blue LED illumination (λ = 470 nm) for pristine and doped hematite photoelectrodes. From these spectra, the quantitative parameters of CSE × LHE (charge separation efficiency versus light harvesting efficiency), EQE (external quantum efficiency), and η_transf_ (charge transfer efficiency) were extracted for Hem, YH, LaH, AlH and GaH, in accordance with IMPS general theory.^48^ The Nyquist plots reveal that, for all samples, the majority of charge carriers suffer recombination at 0.8 *V*_RHE_, which is represented by the semicircle of the upper quadrant returning to the origin. At 1.23 *V*_RHE_, Y^3+^ and La^3+^ modified samples still exhibit limited charge separation efficiency, as the upper loop circle crosses the *x*-axis in small *H*′ values. This is also noticed by the limited CSE × LHE, EQE, and η_transf_ parameters calculated for these samples in comparison to pristine hematite. Particularly for samples with near-zero photocurrent densities, such as LaH, the absence of photogenerated carrier activity at low bias makes the IMPS model prone to apparent fluctuations in η_transf_ at V < 1 *V*_RHE_, which arise from the intrinsic numerical limitations of the fitting procedure under low-current conditions rather than from any real physical processes. For AlH and GaH, however, the application of a 1.23 *V*_RHE_ potential causes the semicircle in the upper quadrant to become much smaller and cross the *x*-axis in more positive *H*′ values, indicating effective separation of photogenerated carriers. This is also revealed by the superior CSE × LHE and EQE values of AlH and GaH in comparison to pristine material, which indicate enhanced charge separation and collection per incident light. In addition, Al^3+^ and Ga^3+^-modified photoanodes exhibited a constant improvement in charge transfer efficiency throughout the analyzed potential range. To understand the superior charge dynamics of AlH and GaH photoelectrodes, Mott–Schottky (M-S) and Raman experiments were proposed.

The calculation of the charge donor density number from M-S plots (Figure S11) revealed that pristine hematite exhibits an N_D_ value in the order of 10^20^ donors per cm^3^ (Table S5). Despite the high charge availability, its photoelectrochemical performance is strongly limited by low carrier mobility, derived from the polaronic domain. LaH, AlH, and GaH photoanodes presented N_D_ values within the same order of magnitude as Hem (10^20^ cm^–3^), while YH showed an order of magnitude lower donor density value (10^19^ cm^–3^). Therefore, the improvement in charge transfer quantified by IMPS analysis for Al^3+^ and Ga^3+^ modified photoanodes is not associated with a greater number of charge donors. To validate the hypothesis that aluminum and gallium doping increase the electron mobility at the grain level through polaron mitigation, Raman experiments were performed. Raman spectroscopy can provide valuable insights regarding polaronic effects in α-Fe_2_O_3_, as small-polaron hopping promotes lattice displacements and structural distortions that could be tracked through the application of the technique.^49^ Given that the localization of small-polarons is wavelength dependent,^50^ being more accentuated upon the irradiation of light with energy similar to hematite’s band gap (2.1–2.2 eV, ∼590–560 nm),^51^ Raman spectra were recorded under blue (λ = 473 nm), green (λ = 532 nm) and red (λ = 638 nm) laser illumination.

Figure S12 shows the spectra obtained for Hem, YH, LaH, AlH, and GaH photoanodes under distinct wavelength irradiation. For all lasers, the characteristic bands attributed to hematite transverse optical vibrations (space group D^6^_3d_)^52^ were observed, as assigned in the spectra of Figure S12a. In addition, when Raman analysis was conducted under green (Figure S12b) and red (Figure S12c) laser illumination, a signal at approximately 660 cm^–1^, not observed in λ = 473 nm, was identified. This signal represents an infrared active longitudinal optical E_u_ mode that, although theoretically inactive in Raman spectroscopy, arises from lattice distortions that lower crystallite symmetry,^53^ being attributed to polaron hopping. The full width at half-maximum (fwhm) of the E_u_ peak at λ = 532 nm for Hem, YH, LaH, AlH, and GaH photoanodes is presented in Table S6. An increase in the fwhm values is observed with the incorporation of Y^3+^, La^3+^, Al^3+^, and Ga^3+^ in comparison to pristine hematite, which is consistent with the reduction of the 660 cm^–1^ peak in doped samples. Recent studies have shown that the interaction of dopants with small polarons can reduce their hopping barriers by promoting a decrease in the lattice expansion,^54^ therefore weakening the E_u_ signal. This shows that lattice dopants are directly interfering with polaronic domains, improving the mobility of electrons. Such modifications lead to enhanced charge separation and transport, as evidenced by the superior performance of AlH and GaH photoanodes, resulting in improved electronic conductivity and overall PEC efficiency.

In summary, the incorporation of lattice dopants through the PPS method yields nanostructures with increased film thickness, porosity, electrochemically active surface area, and intragrain electronic mobility, which was observed regardless of the intrinsic properties of employed chemical modifiers. The following section addresses the incorporation of the same elements into hematite photoanodes as interfacial modifiers, aiming to compare the fingerprints of intentional interfacial segregation to those of lattice doping when the solubility limit of hematite is surpassed.

### Interfacial Segregation

To assess the impact of intentional interfacial modifications on enhancing the electronic conductivity at grain boundaries by forming excesses between grains, and to distinguish it from lattice doping, the same chemical elements—Y^3+^ (HY), La^3+^ (HLa), Al^3+^ (HAl), and Ga^3+^ (HGa)—were incorporated into hematite at a 1.0% molar ratio (relative to Fe^3+^) after precursor solution polymerization, as described in Figure 1c. X-ray diffractograms of pristine and modified hematite photoanodes (Figure S13) show signals assigned to α-Fe_2_O_3_ (JCPDS 33-0664) and SnO_2_ from the FTO conductive layer (JCPDS 41-1445), with no detection of secondary phases attributed to modifier segregation at the crystal surface. The cell parameters of all photoanodes were calculated (Table S7), revealing no significant variations in *a*, *c*, and V values upon dopant addition in comparison to pristine hematite (Hem).

In contrast to the results observed when modifiers were incorporated as lattice dopants, the introduction of external modifiers significantly texturized the materials along the (1 1 0) direction. This is demonstrated by the increase in the texture coefficient of the (1 1 0) plane, which rose from 0.71 for Hem to 0.90, 1.00, 0.80, and 0.76 for HY, HLa, HAl, and HGa, respectively (Figure 3a). Interestingly, interfacial dopants did not induce crystal growth in other directions, as previously seen for lattice doping. These observations suggest that Y^3+^, La^3+^, Al^3+^, and Ga^3+^ preferentially accumulate at the grain surfaces of planes that are orthogonal to (1 1 0), lowering the crystal energy barrier and inhibiting further crystal growth, as noted by the reduction of crystallite size for all modified samples in (1 0 4) and (3 0 0) directions when compared to pristine hematite (Table S7). This behavior is characteristic of dopant migration during thermal treatments, in which modifiers form an excess or segregate at grain boundaries without precipitating a secondary phase.^55^

**Figure 3:**
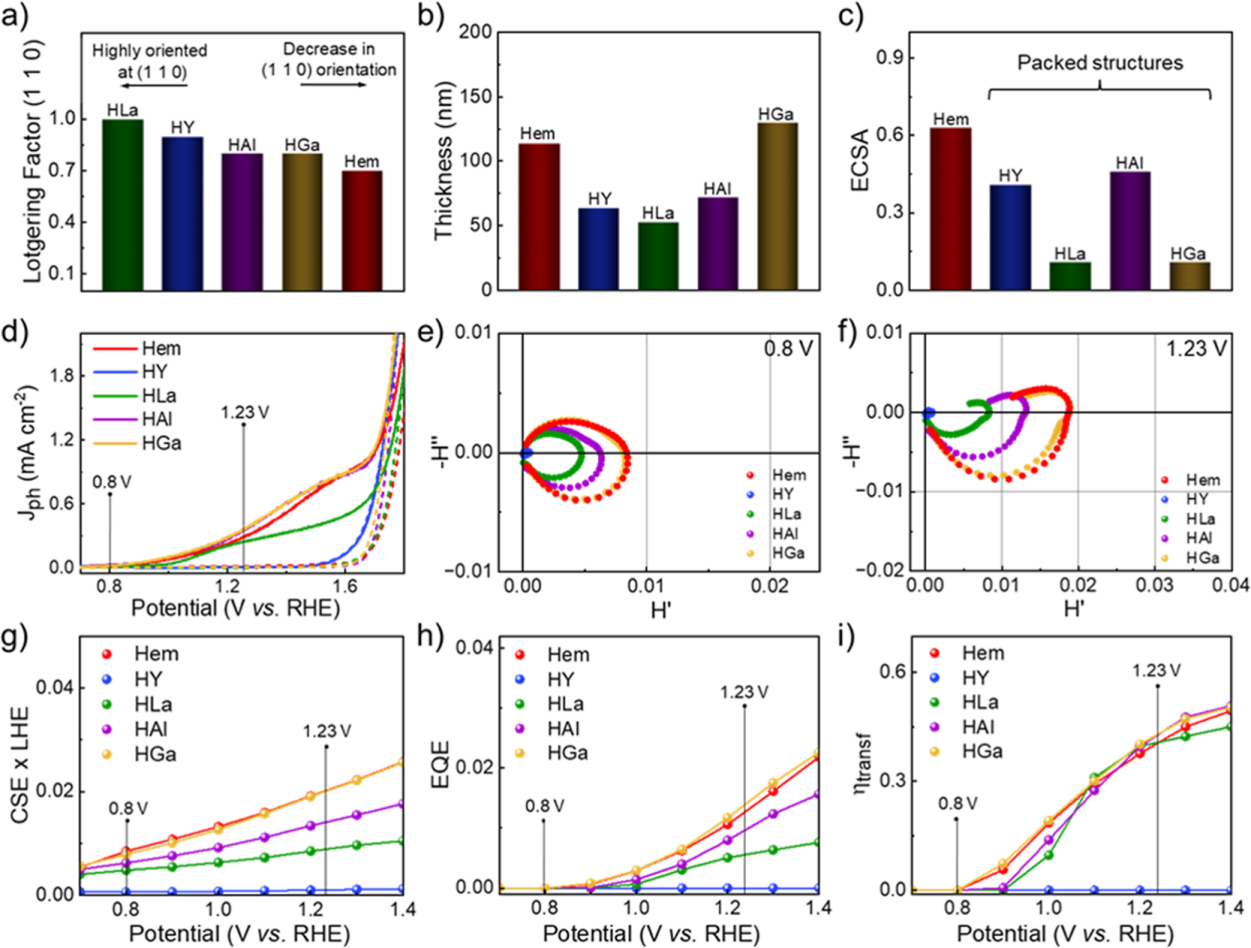
(a) Bar chart displaying the Lotgering factor calculated along the (1 1 0) plane for pristine (Hem) and interfacially modified (Step 2) hematite photoanodes. (b) Estimated thicknesses of Hem, HY, HLa, HAl, and HGa photoanodes, determined from FIB-SEM analysis. (c) Electrochemically active surface area (ECSA) estimated from cyclic voltammetry experiments for pristine (Hem) and interfacially modified (Step 2) hematite photoanodes. (d) Photocurrent density (*j*–*V*) profiles of Hem, HY, HLa, HAl, and HGa photoanodes under simulated sunlight (solid lines) and dark conditions (dashed lines). (e) Nyquist plots obtained from IMPS analysis at 0.8 *V*_RHE_ for Hem, HY, HLa, HAl, and HGa photoanodes. (f) Nyquist plots obtained from IMPS analysis at 1.23 *V*_RHE_ for Hem, HY, HLa, HAl, and HGa photoanodes. (g) Charge separation versus light harvesting (CSE × LHE), (h) external quantum (EQE), and (i) charge transfer (η_transf_) efficiencies derived from IMPS analysis across the 0.7–1.4 *V*_RHE_ potential range.

The cross-section images of the photoanodes modified with yttrium, lanthanum, aluminum, and gallium (Figure S14) showed the segregation trend of grain size pinning, accentuated for the elements with higher ionic radius. Steric hindering during annealing can inhibit coarsening more effectively and therefore deliver smaller nanograins.^56^ YH, LaH, and AlH photoanodes also presented reduced thicknesses in comparison to Hem (Figure 3b). Moreover, the materials modified during Step 2 of the PPS protocol showed a more compact, less porous structure, which is translated into inferior electrochemically active surface areas (Figures S15, S16, and Table S8). ECSA values of 0.41, 0.11, 0.46, and 0.11 were estimated for HY, HLa, HAl, and HGa samples, respectively, as displayed in the bar chart of Figure 3c, contrasting with the ECSA values calculated for lattice-doped materials.

The topographical profiles of hematite photoanodes modified during Step 2 of the PPS protocol revealed the formation of smaller nanograins for structures in which segregation was predominant, as presented in Figure S17. This observation is consistent with ion segregation at interfacial regions of oxide systems. In addition, grain size refinement is commonly linked to a diminution of surface roughness,^57^ which happens due to better packing of grains onto the substrate.^58^ Table S9 puts into perspective the lower mean roughness values observed for HY (15.3 nm), HLa (15.3 nm), HAl (18.1 nm), and HGa (26.1 nm) in comparison to Hem (45.1 nm). Surface roughness values of interfacially modified samples are also lower than the ones observed for lattice-doped photoanodes (Table S4), showing that the grain size reduction resulting from elemental incorporation during Step 2 of the PPS method improves substrate coverage by promoting more efficient grain accommodation within the FTO surface irregularities.

Interestingly, segregation promoted the maintenance of α-Fe_2_O_3_ optical properties (Figure S18a), as shown by the maximum current values permitted by the absorption properties (J_abs_) of the photoanodes (Figure S18b). While pristine hematite presented a J_abs_ of 3.66 mA cm^–2^, the mean value of J_abs_ for the five modified photoanodes was 3.55 mA cm^–2^. Moreover, no alterations in the estimated optical band gap values were observed (Figure S18c). As supported by AFM profiles and FIB-SEM cross-sectional images, modifiers are predominantly acting at grain boundaries, thereby preserving the intrinsic electronic transitions of the hematite matrix.

X-ray photoelectron spectroscopy (XPS) high resolution Fe 2p spectra of interfacially modified photoanodes (Figure S19) once again confirmed the predominance of Fe^3+^ across all samples. However, the characteristic peaks of Y 3d (HY), La 3d (HLa), Al 2p (HAl), and Ga 2p (HGa) were not detected when yttrium, lanthanum, aluminum, and gallium, respectively, were designed to segregate at interfaces (Figure S20). Given that XPS typically probes only a few nanometers of depth, modifiers preferentially allocated beyond this range would remain out of detection range. These results suggest that modifiers incorporated during Step 2 of the PPS synthesis are led to occupy deeper regions of the material, being placed at grain boundaries. While the partial migration of lattice dopants to outer crystal environments results in their diffusion and accumulation at the surface, intentional segregation preferentially leads modifiers to grain–grain interfaces, differentiating the migration of ions that are deliberately placed within different crystal positions. Notwithstanding, it is worth mentioning that signatures of surface enrichment have been photoelectrochemically observed upon the incorporation of higher interfacial dopant concentrations (e.g., 3%), as this accumulation contributes to the creation of surface trapping states.^33^ Similar states may also occur at the interface between the conductive substrate and the deposited layer, promoting increased charge carrier recombination at the back contact.^59^

Figure 3d shows, using photoelectrochemical measurements as a probe, that the behavior of the modifying elements added during Step 2 is different from when they were added during Step 1. HY presented a null photocurrent density, opposing the *J*_ph_ value of 0.21 mA cm^–2^ at 1.23 *V*_RHE_ observed in YH. HLa, on the other hand, showed a *J*_ph_ value of 0.23 mA cm^–2^ at 1.23 *V*_RHE_. Although still presenting a photocurrent density lower than hematite, lanthanum segregation displayed an inverse trend in comparison to lattice doping, in which the observed photocurrent was null. Aluminum (*J*_ph_ at 1.23 *V*_RHE_ = 0.30 mA cm^–2^) and gallium (*J*_ph_ at 1.23 *V*_RHE_ = 0.32 mA cm^–2^) exhibited photoresponses similar to that of pristine hematite when assembled as interfacial modifiers. The superior performance of AlH and GaH electrodes in comparison to HAl and HGa, aligned with Raman results, suggests that an enhanced solar water splitting efficiency is achieved for those cations only when promoting a symmetry-break effect in the Fe^3+^ chemical environment as lattice dopants, thus alleviating hematite’s polaronic domain.

The IMPS Nyquist plots in Figure 3e,f depict the charge recombination behavior of the interfacially modified samples at potentials of 0.8 and 1.23 *V*_RHE_, respectively. These potentials are emphasized because 0.8 *V*_RHE_ corresponds to the onset potential for charge transport, whereas 1.23 *V*_RHE_ represents the thermodynamic potential for water splitting. Semicircles returning to the origin indicate complete charge recombination, whereas intersections of the *x*-axis at more positive values reflect enhanced charge separation. Once again, these plots were used as the basis for applying the IMPS mathematical model to extract quantitative parameters (Figure 3g–i), providing deeper insight into the charge-carrier dynamics of the photoanodes and enabling a comprehensive interpretation of their photocurrent behavior.

From Nyquist plots, it is possible to notice that the majority of photogenerated charge carriers are recombined in HY upon the application of 0.8 *V*_RHE_ (Figure 3e) and 1.23 *V*_RHE_ (Figure 3f) potentials, which results in its negligible photocurrent density. This is also represented by the very low charge separation efficiency, external quantum efficiency, and charge transfer efficiency in the 0.7–1.4 *V*_RHE_ potential window for this sample (Figure 3g–i). HLa, on the other hand, exhibited an improvement in charge separation and transfer efficiencies at 1.23 *V*_RHE_ in contrast to 0.8 *V*_RHE_, despite exhibiting overall values lower than pristine material. HAl and HGa displayed CSE × LHE, EQE, and η_transf_ values similar to Hem, consistent with their comparable photocurrent densities. Furthermore, Mott–Schottky analysis (Figure S21) showed that charge donor densities were not increased by the addition of interfacial modifiers (Table S10), and the Raman spectra of interfacially modified samples revealed no displacements in the E_u_ mode at 660 cm^–1^ compared to Hem (Figure S22 and Table S11). The absence of short-range nanostructure disorder is a strong indicative that the chemical elements incorporated during Step 2 of the PPS method are not actively interfering within the polaronic domain in hematite.

To further unveil the segregation trends in the photoelectrochemistry of interfacially modified photoelectrodes, 13 other elements (Table S1) were assembled as interfacial modifiers. This time, chemical elements with lower (+1 and +2) and higher oxidation states than iron (+4 and +5) were also investigated. Figure S23 shows that none of the tetravalent and pentavalent elements exhibited photoresponses lower than pristine hematite, and most of them caused the onset of the oxygen evolution reaction to be shifted toward more positive potentials. This increased overpotential is consistent with the rise of surface trapping states due to surface enrichment with modifiers.^60,61^ Such trend was not observed for elements with lower oxidation states (≤+3), hinting the importance of the physicochemical properties of the elements for an assertive choice depending on the desired application. Here, we restrict ourselves to reporting only how the synthesis method selectively allocates the dopants, regardless of the final effect on material applications.

The differences between intentional interfacial segregation and dopant accumulation resulting from exceeding hematite’s dopant solubility limit were also observable by high-angle annular dark-field scanning transmission electron microscopy (HAADF-STEM) coupled with energy dispersive spectroscopy (EDS) and electron energy loss spectroscopy (EELS), as depicted in Figure 4. The heavy element lanthanum was selected for proof-of-concept, and lamellae of HLa and LaH were prepared by FIB-SEM for this analysis. While tin was only found in the FTO substrate region and iron is homogeneously distributed throughout the grain with EELS binding energy of approximately 844.0 eV at the Fe L_1_ edge for both samples, lanthanum chemical maps remark the dopant positioning driven by the step of the PPS method in which they were added.

**Figure 4:**
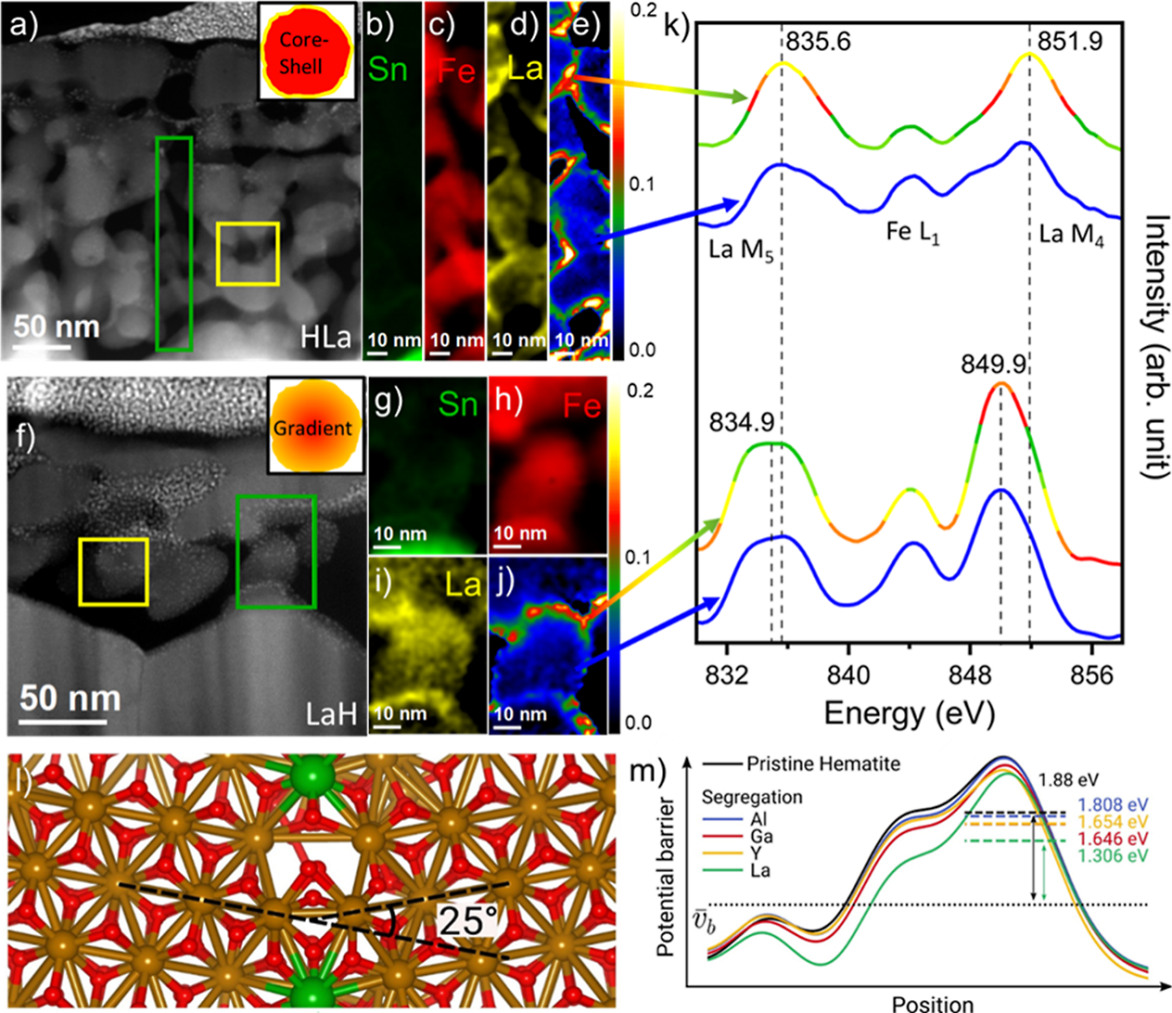
(a,f) High-angle annular dark-field scanning transmission electron microscopy (HAADF-STEM) spectra of (a) HLa and (f) LaH photoanodes. The figure inset illustrates the grain-dopant distribution in each sample. The yellow areas represent spatial drift regions, and green areas denote sample regions analyzed through energy dispersive X-ray spectroscopy (EDS). EDS analysis shows the distribution of (b,g) Sn, (c,h) Fe, and (d,i) La in the photoanodes. (e,j) Show the ratio between La/Fe peaks obtained from EDS analysis. (k) Electron energy loss spectroscopy (EELS) spectra obtained for HLa (top) and LaH (bottom) photoanodes. La M_5_, Fe L_1_, and La M_4_ peaks were extracted from the regions indicated by the arrows (blue and colored areas of each sample). (l) Atomistic model of the 25° tilt grain boundary in hematite used for density functional theory (DFT) calculations, with Fe (gold), O (red), and generic segregant atoms (green) at the interface. (m) Calculated effective potential energy barriers across the grain boundary for pristine hematite (1.88 eV) and hematite with segregated Al^3+^ (1.808 eV), Ga^3+^ (1.654 eV), Y^3+^ (1.646 eV), and La^3+^ (1.306 eV). Barriers are shown relative to the bulk mean potential (ν_b_).

For HLa (Figure 4a–e), lanthanum was mainly detected at interfaces. The determination of La/Fe peaks intensity ratio (Figure 4e) exhibited a high La^3+^ accumulation at grain boundaries (very intense warm colors at grain–grain interfaces), as expected for segregation. The EELS spectra (Figure 4k, top) showed binding energies of 851.9 and 835.6 eV for La M_4_ and M_5_ edges, respectively. The measured binding energies of M_4_ and M_5_ signals were the same in grain–grain interface regions (colored curve, region indicated by the colored arrow) and within hematite grains (blue curve, region indicated by the blue arrow), being consistent with La^3+^ oxidation state.^62^

On the other hand (Figure 4f–j), lanthanum was preferentially distributed throughout hematite grains in LaH (Figure 4i), forming a uniform gradient with iron (Figure 4h). The calculation of La/Fe peaks intensity ratio (Figure 4j) revealed a partial allocation of La^3+^ at the surface, as previously seen by XPS analysis. Nevertheless, the accumulation of lanthanum at grain boundaries was much less pronounced than in HLa, as depicted by the less intense warm colors at grain–grain interfaces. The EELS spectra (Figure 4k, bottom) acquired at regions comparable to HLa (within the grain and at the grain boundary) showed a shift in La M-edges in contrast to the photoanode with intentionally designed segregation: the M_4_ edge shifted to 849.9 eV, while the M_5_ edge shifted to 834.9 eV. While these observed energy values are still consistent with the lanthanum +3 oxidation state, the energy shift confirms that La^3+^ ions in LaH have different bonding states with neighboring atoms compared to HLa, reinforcing the occupancy of a different chemical environment for lanthanum in those samples.

Density functional theory (DFT) analysis was performed on a representative hematite grain boundary mode to further prove the unique effect of intentional segregation. Following the methodology proposed for hematite GB interfaces,^63^ a 25° tilt GB was examined (Figure 4l). For pristine hematite, these calculations reveal a significant potential energy barrier for electron transport across the GB, with a height (V_0_) of 1.88 eV relative to the bulk mean potential, as depicted in Figure 4m. The incorporation of Al^3+^, Ga^3+^, Y^3+^, and La^3+^ at this GB dramatically changed the potential barrier landscape, reducing V_0_ to 1.808, 1.654, 1.646, and 1.306 eV, respectively. These substantial reductions can be attributed to the lower local potential energy associated with the higher effective atomic charge at the GB.

Considering that the transmission coefficient (T_i_) for electron transport across a potential barrier, such as the Schottky-type found in hematite polycrystalline nanostructures,^64^ can be approximated using a quantum tunneling model for a rectangular barrier, the reduction in barrier height from V_0_ (pristine material) to V_1_ (segregation at the GB) can be linked to an enhanced T_i_. This assumption also considers a typical GB barrier width (*a*) of approximately 2.5 Å and a conduction band minimum effective mass (*m* ^*^) of 4.1*m*_0_ for hematite.^63^ Since the intergrain conductivity in polycrystalline materials with nonpercolating grains is directly dependent on this electron transmission rate between grains, the DFT results provide a fundamental understanding of how intentional segregation of specific modifiers to grain boundaries effectively lowers electronic barriers and increases electron tunneling probability, which, in turn, facilitates intergrain charge transport. An important disclaimer is that, although the final property (e.g., electronic conductivity) is dependent on the physicochemical properties of the elements, the behavior of segregated elements in the nanostructure (reduced V_0_) was shown to be independent from the element’s nature, reinforcing that the dopant placement driven by the PPS synthesis method comprises a tailored strategy for a targeted goal (formation of interfacial excess).

Therefore, the fingerprints of chemical modifications during Step 2 of the PPS protocol (Figure 1c) combine the changes caused by modifiers in the kinetic and thermodynamic processes of coarsening (sintering, coalescence, and grain growth), resulting in more compact structures with smaller nanograins, reduced surface roughness and lower electrochemically active surface area. Such observations differ from those associated with chemical modifications at Step 1 (Figure 1b), indicating that the intentional placement of modifiers at interfaces results in materials with distinct properties from when partial ions are exuded from within the crystal to the surface due to exceeded doping solubility limits. The following section further expands this concept by showcasing specific dopant choices that optimized the performance of hematite photoanodes.

### Case Study for Dopant Choices Targeting High PEC Performance on Hematite

As hematite often requires multiple surface and bulk modifications to address its intrinsic limitations, employing the polymeric precursor synthesis (PPS) method to selectively incorporate targeted dopants can enable precise tuning of electronic and structural properties. This approach highlights the critical role of dopant selection in mitigating key drawbacks—such as poor charge transport and short hole diffusion length—thereby significantly enhancing the photoelectrochemical performance of hematite under simulated sunlight conditions.

We have previously shown that a combination of 0.5% of Al^3+^ and 3.0% of Zr^4+^ effectively addressed hematite’s conductivity bottlenecks and delivered a photoanode (AlHZr) with a 4-fold current density enhancement at 1.23 V vs RHE compared to pristine hematite.^28^ Aluminum ions (Al^3+^) were selected as lattice dopants due to their similar ionic radius to Fe^3+^, thereby minimizing structural distortion while suppressing polaronic effects and enhancing intragrain charge mobility. Zirconium (Zr^4+^) was introduced as an interfacial modifier given its higher oxidation state, aiming to increase the conductivity at grain boundaries.

Our results showed that Al^3+^, incorporated during Step 1 of the protocol, was homogeneously distributed within the grains, whereas Zr^4+^, incorporated at Step 2, was primarily found at grain boundaries (Figure S24a).^28^ Interestingly, the current density shown at Figure S24b for AlHZr (3.65 mA cm^–2^ at 1.23 *V*_RHE_) exceeded the sum of the individual improvements achieved through single modifications (lattice doping or interfacial segregation) and is comparable with reported literature breakthroughs (see ref 28, Table S5). This outstanding performance was stable during 48 h of PEC operation (Figure S24c), using almost 100% of the photocurrent generated for O_2_ and H_2_ production (Figure S24d). Additionally, the surface composition integrity of the sample was maintained during the long-term stability test (Figure S25a–e), with preliminary studies confirming that ion leaching from the photoanode to the electrolyte did not exceed 0.5%—which corresponds to maximum leachate concentrations of 1.4 mg L^–1^ for Fe, 0.006 mg L^–1^ for Al, and 0.14 mg L^–1^ for Zr.

Ecotoxicological evaluations with *Daphnia similis* (Figure 5a) further probed the environmental-friendly characteristics of these materials. Dose–response curves constructed for Zr^4+^, Al^3+^, and Fe^3+^ (Figure 5b,c) show that the highest ion concentrations at which no adverse effects were observed (NOAEL) were 2.0 mg L^–1^ for Fe^3+^, 0.5 mg L^–1^ for Al^3+^, and 0.2 mg L^–1^ for Zr^4+^, and that the concentration that inhibited the mobility of 50% of daphnids (EC_50_) after 48 h was 6.8 mg L^–1^ for Fe^3+^, 1.5 mg L^–1^ for Al^3+^, and higher than 0.2 mg L^–1^ for Zr^4+^. The estimated maximum ion concentrations from photoanode leaching remained below the NOAEL for *Daphnia*, revealing that, under the studied conditions, leaching would occur at nontoxic levels to aquatic organisms, a very desirable feature toward sustainable technology transfer and scalability.

**Figure 5:**
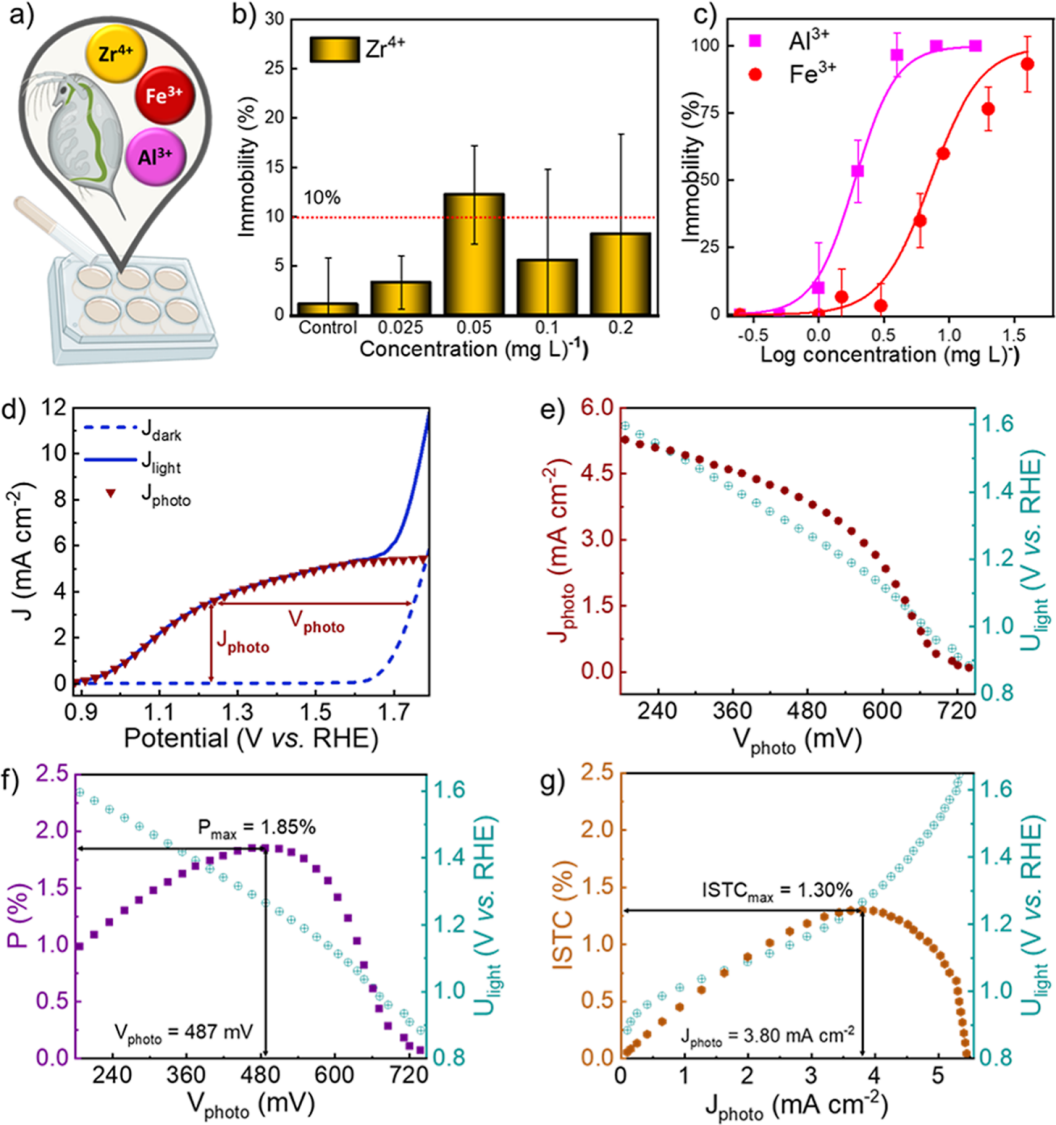
(a) Representation of the nanotoxicology study performed with *Daphnia similis.* Daphnids were exposed to Fe^3+^, Al^3+^, and Zr^4+^ solutions to assess ion toxicity. The immobility of organisms was monitored and recorded as a function of ion concentration. Negative controls (Daphnids exposed to reconstituted water) were assessed for validity, with the mortality of nonexposed organisms remaining below 10%. Dose–response curves obtained for (b) Zr^4+^ and (c) Al^3+^ and Fe^3+^ ions. Metal ions were tested at increasing concentrations, with five *Daphnia similis* neonates per concentration. Each concentration was tested in six replicates within the same assay, and the entire assay was independently repeated at least four times. (d) *j*–*V* curves obtained for the dual modified AlHZr photoanode in dark conditions (blue dashed line) and under 100 mW cm^–2^ simulated solar illumination (blue solid line). The generated photocurrent (*J*_photo_) was determined by extrapolating the J_light_ curve in the region where no electrolysis contribution is observed. (e) Generated photocurrent (*J*_photo_) of the dual modified AlHZr photoanode and applied potential under light conditions (*U*_light_) as a function of the photopotential (*V*_photo_). (f) Intrinsic photovoltaic power (*P*) of the dual modified AlHZr photoanode and applied potential under light conditions (*U*_light_) as a function of the photopotential (*V*_photo_). (g) Intrinsic solar to chemical conversion efficiency (ISTC) of the dual modified AlHZr photoanode and applied potential under light conditions (*U*_light_) as a function of the generated photocurrent (*J*_photo_).

Considering the assessed potential of AlHZr for application in PEC devices, an in-depth performance analysis based on the intrinsic solar-to-chemical conversion efficiency (ISTC) of the material^65^ was conducted (Figure 5d–g). The dual modified photoanode exhibited a maximum photovoltaic power (P, Figure 5f) of 1.85 mW cm^–2^ at a photopotential of 487 mV, i. e., 1.85% of the 100 mW cm^–2^ simulated sunlight reaching the sample was converted into electrical power. This indicates that the light-induced power generated by the sample reduces the need for an external power source (in this case, a potentiostat) by approximately 0.49 V.

Moreover, the maximum P value obtained for AlHZr corresponded to an ISTC of 1.30%, as shown in Figure 5g. This efficiency reflects the power generated within the photoanode that contributes to solar-to-chemical energy conversion, taking into account the efficiency of the photoelectrolysis process. Hence, 1.30 mW cm^–2^ of the 1.85 mW cm^–2^ power generated by the sample contributes to the energy conversion process, showcasing the energy economy sustained by the use of a highly efficient photoabsorbing semiconductor in water electrolysis devices.

The nanoscale mechanisms underlying the outstanding photoelectrochemical performance of the optimized AlHZr photoanode (i.e., enhanced intragrain mobility from lattice doping and improved charge transport at grain boundaries via interfacial segregation) can be represented in terms of the overall conductivity (σ_T_) of a polycrystalline metal oxide semiconductor. Equation 1 shows σ_T_ as a function of intragrain conductivity (σ_G_) and conductivity at grain boundaries (σ_GB_).

1 $$\sigma_{T}=\sigma_{G}+\sigma_{GB}$$

Figure 6 depicts a scheme of the overall effects of lattice doping, interfacial segregation, and dual modification designed by the PPS methodology in hematite-based materials. It summarizes the discussion thoroughly scrutinized throughout this work and serves as a guide to use the PPS method to effectively achieve specific structural and electronic modifications in multi-interfaced materials. For pristine polycrystalline hematite (Figure 6a), the electron effective mass (*m*_e_^*^/*m*_0_) in the lattice is close to 16.4 in d orbitals,^66^ rather large value derived from strong electron–phonon couplings that cause charge carriers to be self-trapped. In such case, intragrain charge mobility (μ_e_) and conductivity (σ_G_) are hindered (represented by dashed red arrows), resulting in slow carrier movement that promotes high recombination rates. Furthermore, highly energetic interfaces (Schottky-type barriers at hematite’s grain boundaries) continue to restrict carrier diffusion through the polycrystalline grains of α-Fe_2_O_3_ (↓σ_GB_), hindering grain–grain transport (represented by the red × symbol). The addition of chemical modifiers during Step 1 (Figure 1b) of the PPS protocol (Figure 6b) promotes a homogeneous distribution of metal cations throughout hematite grains, contributing to their preferential intragrain allocation. This selective incorporation of dopants provokes a local break in the symmetry of the Fe^3+^ environment due to fluctuations in iron bond lengths, globally changing *m*_e_^*^/*m*_0_ in the lattice and enhancing electron mobility.^67^ Consequently, intragrain conductivity is increased (↑σ_G_), as represented by thick solid arrows.

**Figure 6:**
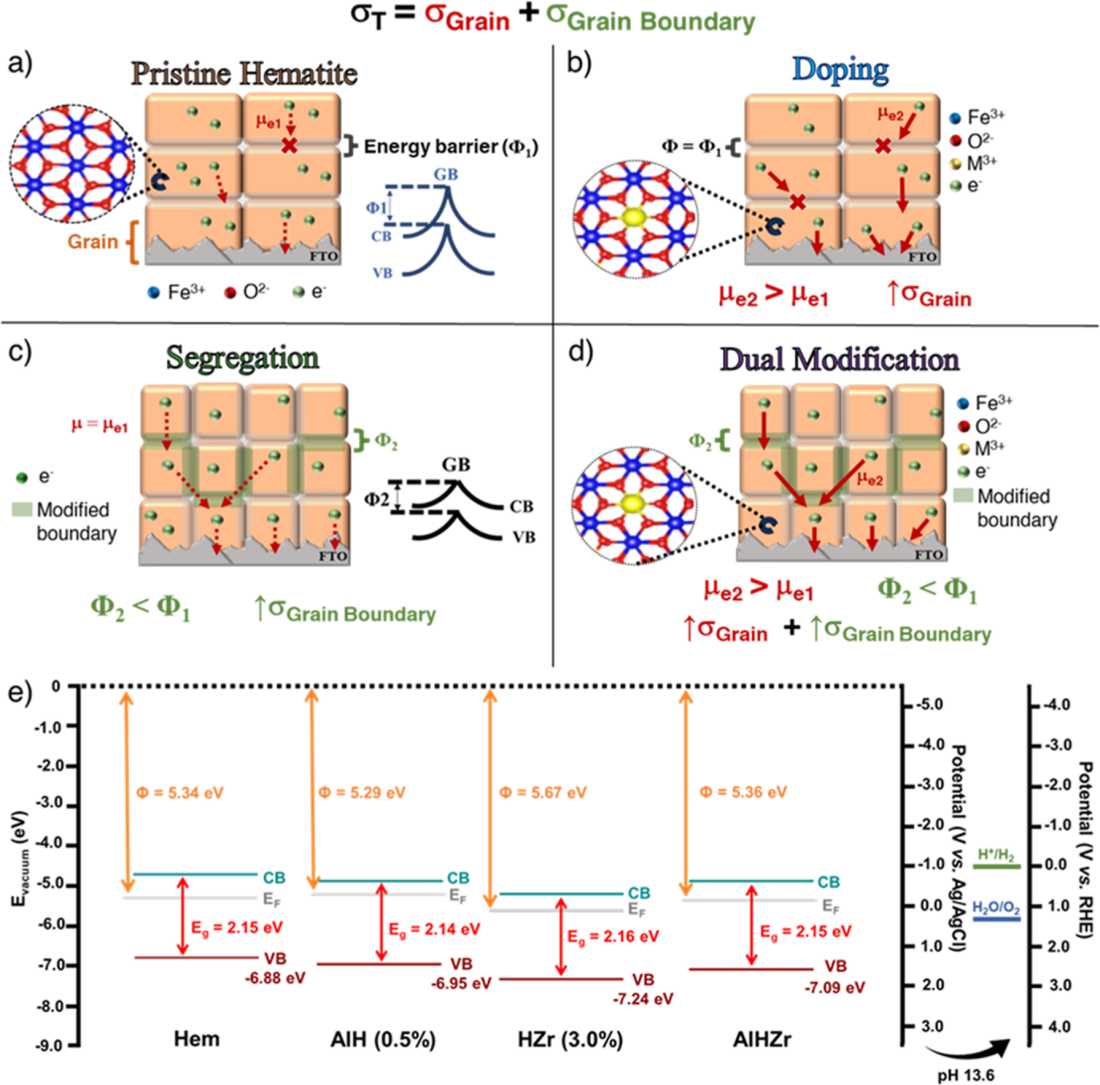
(a) Schematic representation of polycrystalline pristine hematite nanostructures. The intragrain charge carrier mobility of the pristine material is strongly hindered by small-polaron formation, while grain–grain charge transport is limited by highly energetic grain boundaries (GB). (b) Schematic representation of lattice doping effects in hematite photoanodes. Dopant integration mitigates the polaronic domain and increases intragrain conductivity. (c) Schematic representation of interfacial segregation effects in hematite photoanodes. The allocation of chemical modifiers at GB decreases interfacial energy barriers, allowing for improved intergrain charge transport. (d) Schematic representation of dual modifications (simultaneous lattice doping and interfacial segregation) effects in hematite photoanodes. This approach can concurrently address intragrain and intergrain bottlenecks of polycrystalline hematite-based nanostructures. (e) Band diagrams of pristine hematite (Hem), AlH (0.5%), HZr (3.0%), and AlHZr photoanodes. The valence band (VB) maximum and the work function (Φ) values were extracted from the expansion of the region near the Fermi level and the determination of the secondary electron cutoff of the UPS spectra of the photoanodes (Figure S26). Gap energies (Eg) were extracted from Tauc plots (Figure S27).

While intragrain charge mobility is enhanced by lattice doping, the segregation of modifiers at the grain boundaries (Figure 6c), caused by the addition of chemical elements during Step 2 (Figure 1c) of the PPS protocol, allocates chemical modifiers at solid–solid interfaces (represented by green colored interfaces), reducing the energy barriers that pose a challenge to the electron conduction through hopping mechanism. This is beneficial if segregation to the outer film surface is not relevant. DFT calculations confirmed that segregation lowers the potential barrier at grain boundaries (Figure 4m), enhancing electron tunneling probability and thus facilitating better carrier transport through grains. For zirconium, by lowering the energy barrier height (V_0_) to 1.48 eV compared to 1.88 eV of pristine hematite, the transmission coefficient (T_i_) for electron transport increased by approximately 3-fold.^28^ Even for electrons with energies above the original pristine barrier (E > V_0_), T_i_ was enhanced by ∼1.5-fold. Consequently, grain boundary electronic conductivity and charge separation efficiency are markedly improved (↑σ_GB_). Following this strategy, intragrain charge transport may still be somewhat restricted (↓σ_G_) due to lower electronic mobility (represented by thin dashed lines).

By simultaneously promoting lattice doping with Al^3+^ and interfacial segregation with Zr^4+^, a synergistic effect was achieved from the combination of enhanced intragrain electronic conductivity (σ_G_) and improved conductivity at grain boundaries (σ_GB_). Figure 6d schematically represents the dependence of the material’s overall conductivity (σ_T_) on both σ_G_ and σ_GB_. In this case, small polaron domain is mitigated concomitantly with interfacial energy barrier lowering, yielding a dual modified nanomaterial with enhanced generation and separation of electron–hole pairs, coupled with improved charge transport and diffusion.

The electronic effects resultant from selective dopant addition were assessed by experimental band diagrams obtained from ultraviolet photoelectron spectroscopy (UPS, Figure S26) and optical gap energies (E_g_, Figure S27) previously estimated. Upon lattice dopant incorporation, the possible introduction of localized states within the bandgap (e.g., due to d-orbitals) complicates the simple donor–acceptor charge equilibrium, as this may lead to Fermi level pinning, shallow or deep states, and modification of electronic conductivity, even interfering with defect formation energies (like oxygen vacancies).^68^ With all these phenomena, the semiconductor reaches a new thermodynamic equilibrium, and the Fermi level adjusts to reflect the new balance of occupied and unoccupied electronic states. AlH (0.5%) sample (Figure 6e) exhibited lower work function (Φ) value in contrast to Hem, representing an upward shift of the Fermi level in the direction of the conduction band, favoring electron injection to the external circuit for water splitting, for example. Conversely, Fermi level shifts were also markable for lattice-doped samples (Figures S28 and S29), meaning that, despite the individual properties of the chemical elements, the dopant role directed by the method will affect the semiconductor thermodynamic equilibrium, with the magnitude and shift direction depending on dopant valence and orbital character.

Meanwhile, the main effect observed for HZr (3.0%) sample was a shift in the energy level of the valence band maximum (VBM) relative to vacuum (Figure 6e). This phenomenon is mainly driven by band bending and dipole formation at the crystal surface/interface, which affects how energy levels appear with respect to vacuum level, even though the Fermi level remains mostly fixed in the bulk (segregated dopants do not alter bulk carrier densities).^69^ Consequently, water oxidation performance is usually enhanced, as the upward shift in VBM (closer to vacuum level) makes the hole energy more positive (i.e., a stronger oxidizing potential is achieved). A similar trend was observed for other elements intentionally designed for segregation (Figures S30 and S31).

Overall, the particularity of some chemical elements considered, the selective incorporation of dopants promoted by the PPS protocol represents a significant advancement in the development of high-performance multi-interfaced functional materials based on metal oxides. The integration of doping and segregation strategies in hematite photoanodes effectively addresses the limitations of intragrain and interfacial conductivity, precisely manipulating metal oxide nanostructures’ integrity, morphology, electronic, structural, and catalytic properties. This achievement gains relevance when the dopants selected for case studies, such as Al, Zr, Ga, and Y, have been extensively studied but always faced drawbacks to unlock hematite’s potential for solar water splitting, yielding limited PEC performances (see Table S12 and Figure S32). Here, we could show that a carefully designed synthesis method that accounts for the main drawbacks of a multi-interfaced metal oxide is fundamental to the advancement of materials processing and applications. Subsequently, guided by the knowledge acquired in this work, we now hope to focus on trying to predict dopant combinations that are suitable for delivering materials properties on demand toward a targeted application.

## Conclusion

This study applied a polymeric precursor solution (PPS) method for the fabrication of nanostructured polycrystalline metal oxides, enabling on-demand modulation of materials properties based on selective lattice doping and interfacial segregation. Using hematite as model system, lattice doping was found to alter the material’s morphology, increasing film thickness and porosity while maintaining the average grain size. Additionally, enhanced optical absorption was observed with dopant incorporation, and intensity-modulated photocurrent spectroscopy showed element-specific variations in charge transfer efficiencies. This is attributed to alterations in polaronic effects due to symmetry-breaking in the host cation environment, which changes intragrain charge carrier mobility.

Conversely, the incorporation of chemical modifiers during Step 2 of the PPS protocol led to their accumulation at grain boundaries. This interfacial segregation introduced steric hindrance, resulting in interface-rich structures with refined grain sizes that improve film packing on rough surfaces. The application of this strategy effectively reduced Schottky energy barriers at grain–grain interfaces, as confirmed by DFT calculations, leading to increased intergrain electronic conductivity, optimized electron migration between grains, and a significant boost in photocurrent density due to improved photogenerated charge separation.

When appropriately integrated, the combination of lattice doping and interfacial segregation strategies was able to synergistically to improve the overall (intragrain and intergrain) conductivity of metal oxide nanostructures. The achievement of improved performance through this dual approach, however, depended on the careful selection of dopant elements, proven to be both deliberate and application-targeted considering their chemical behavior, solubility limits, and interaction with the host lattice and interfacial environment. As a proof of concept, we demonstrated that codoping hematite photoanodes with Al^3+^ and Zr^4+^ significantly enhanced its photoelectrochemical performance, adding up increased electronic mobility given by aluminum doping and the reduction of interfacial energy barriers through zirconium segregation. The resulting material was nontoxic and yielded 1.30% intrinsic solar-to-chemical conversion efficiency, marking its potential for application in PEC devices. Notably, the PPS method here described provides a scalable framework for designing metal oxide nanostructured films tailored to specific applications, including photoelectrochemical systems.

## Experimental Section

### Substrate Cleaning

Substrates of aluminum borosilicate glass coated with F/SnO_2_ (FTO, Solaronix, 8–10 Ω cm) were successively washed with Extran (100 °C), ultrapure water (100 °C), acetone (60 °C) and isopropyl alcohol (70 °C). The washing procedure involved immersing the substrates in aforementioned solutions, each maintained at the specified temperature, for 30 min. In sequence, washed substrates were thermally treated at 550 °C in air atmosphere for 60 min (Lindberg/Blue M Mini-Mite horizontal tube furnace). This cleaning process was adopted to remove impurities that could affect substrate conductivity.

### Precursor Solution Preparation

Pristine and chemically modified hematite precursors preparation followed the recent and optimized method patented by our group (INPI BR102023005372-6).^70^ The synthesis of the pristine material consisted in dissolving citric acid (C_6_H_8_O_7_, Sigma-Aldrich, 99.5%) in ultrapure water (18.2 MΩ cm, 25 °C) in the ratio of 1:2 (g/mL) under constant magnetic stirring that was throughout maintained. To this solution, iron nitrate (Fe­(NO_3_)_3_·9H_2_O, Sigma-Aldrich, 98%) was added in the molar ratio of 1:3 in relation to citric acid (1Fe^3+^:3C_6_H_8_O_7_). This stage was highlighted as Step 1 in the synthetic process. The system was then heated to 60–70 °C and ethylene glycol (C_2_H_6_O_2_, Sigma-Aldrich, 99.8%) was added in the percentage ratio of 60:40 (mass/mass) in relation to citric acid. Heating was performed on a hot plate equipped with a magnetic stirrer, and the temperatures of both the plate and the solution were continuously monitored using an alcohol thermometer and a K-type wired thermocouple, respectively. Thereafter, maintaining the temperature between 60 and 70 °C, the solution was concentrated by water evaporation until its volume reached 50% of the initial value. The temperature gradient between the plate and the solution was controlled to regulate the evaporation kinetics, and the final volume was verified to ensure accurate reduction to half of the original volume. This stage of obtaining a concentrated precursor was named Step 2 in the synthetic process. After the solution cooled down to room temperature (25 °C), an aliquot of 5.0 mL was reserved and diluted with 1.5 mL of anhydrous ethanol (Sigma-Aldrich, 99.8%) and 1.0 mL of isopropyl alcohol (Sigma-Aldrich, 99.5%). The solution was stirred for 15 min for complete homogenization and stored under refrigeration (7 °C) for a minimum period of 24 h before deposition.

Modified hematite precursors were produced by adding metallic cations during either Step 1 or Step 2 of the solution preparation. For Step 1, the four salts highlighted in Table S1 were added stoichiometrically along with Fe^3+^ salt in aqueous citric acid solution to achieve a 1.0% molar ratio (metal/Fe^3+^ relation) concentration. For Step 2, 18 elements were added as modifiers. Before its addition, ethanolic solutions of all precursor salts shown in Table S1 were prepared. Five mL of the concentrated precursor was reserved and heated up to ∼65 °C, and ethanolic solutions containing the modifying elements were added to achieve a concentration of 1.0% cation/Fe^3+^ molar ratio. The volume of ethanol added to dilute the precursor (1.5 mL) was maintained by discounting the amount of ethanol added from the modifier solution.

### Film Deposition

A volume of 100.0 μL of polymeric solution was spin-coated onto previously cleaned FTO substrates. A first spinning program of 500 rpm for 5 s was employed, followed by 7000 rpm for 30 s. After coating, a small section of the film was cleaned using a cotton swab to expose the FTO surface for electrical contact. The films were then dried on a hot plate at 90 °C for 5 min.

### Thermal Treatments

After drying in the hot plate, films were annealed at 550 °C in air atmosphere for 30 min (Lindberg/Blue M Mini-Mite horizontal tube furnace) to remove polymer organic compounds and promote hematite phase formation. In sequence, the photoanodes underwent thermal treatment at 750 °C under N_2_ flux (150 mL min^–1^) for 30 min (MTI Corporation tubular furnace with automatic sliding quartz, model OTF-1200X-50-SL) to activate film surface for photoelectrochemical applications.

### Photoanodes Characterization

Details about the structural, optical, morphological, electrochemical and photoelectrochemical characterizations performed on hematite-based photoanodes can be found in the Experimental Section of the Supporting Information file.

### Density Functional Theory (DFT) Calculations

First-principles calculations based on Density Functional Theory were performed using the Vienna Ab initio Simulation Package (VASP).^71^ Details about the employed methodology can be found in the Experimental Section of the Supporting Information file.

### Ecotoxicity Evaluation

Ecotoxicity tests using *D. similis* were carried out as a proactive approach to evaluate the potential environmental risks associated with the chemical waste disposal from photoelectrochemical cells. Details about the employed methodology can be found in the Experimental Section of the Supporting Information file.

## Supplementary Material



## Notes

Experimental details for structural, optical, morphological, electrochemical, and photoelectrochemical characterizations; experimental details for density functional theory (DFT) calculations and ecotoxicity tests; DSC, XRD, FIB-SEM, cyclic voltammetry, AFM, UV–Vis spectroscopy, XPS, Mott–Schottky, Raman, and UPS analyses of lattice-doped and interfacially modified hematite photoanodes; photocurrent density profiles of hematite photoanodes modified with M^+^, M^2+^, M^3+^, M^4+^, and M^5+^ metal cations; HAADF-STEM, EDS, linear sweep voltammetry under dark and illuminated (100 mW cm^–2^) conditions, 48 h chronoamperometry, gas evolution, and XPS analyses before and after the stability test for the AlHZr dual-modified photoanode; comparative literature review of the performance of chemically modified hematite photoanodes synthesized using distinct methods (PDF)
