# Supplementary material for: Rational Design of Metal Oxide Nanostructures via Dopant Control: A Case Study in Photoelectrochemical Performance
Source: ACS Appl Mater Interfaces. 2025 Nov 13;17(48):65976–92. doi: 10.1021/acsami.5c16912 (PMC12679547; doi:10.1021/acsami.5c16912)
Supplement: Supplementary file 1 [file am5c16912_si_001.pdf]

SUPPORTING INFORMATION

Rational Design of Metal Oxide Nanostructures via Dopant Control: A Case Study in Photoelectrochemical Performance

*Mariana A. Dotta[1,2]†, Fabio A. Pires[1,2]†, Karen C. Bedin[1], Ingrid Rodríguez-Gutiérrez[1], Francine Coa[1], Heloisa H. P. Silva[1,3], Gabriel R. Schleder[1,5], Carolina P. Torres[1], Fabiano E. Montoro[1], Diego S. T. Martinez[1], Jefferson Bettini[1], Edson R. Leite[1], Renato V. Gonçalves[4], Flavio L. Souza[1,2,5]**

[1] Brazilian Nanotechnology National Laboratory (LNNano), Brazilian Center for Research in Energy and Materials (CNPEM), Campinas, São Paulo, CEP 13083-100, Brazil

[2] Institute of Chemistry (IQ), State University of Campinas (UNICAMP), Campinas, São Paulo, CEP 13083-970, Brazil

[3] School of Technology (FT), State University of Campinas (UNICAMP), Limeira, São Paulo, CEP 13484-332, Brazil

[4] São Carlos Institute of Physics (IFSC), University of São Paulo (USP), São Carlos, São Paulo, CEP 13566-590, Brazil

[5] Humanities and Nature Science Center (CCNH), Federal University of ABC (UFABC), Santo André, São Paulo, CEP 09210-580, Brazil

† These authors contributed equally to this work.

**Corresponding author: flavio.souza@lnnano.cnpem.br*

GENERAL SUMMARY

The Supporting Information file is organized into subsections to better guide reading. Each section mirrors the titles used in the manuscript text to facilitate navigation and contextual understanding. The contents are as follows:

1. Results and discussion 3

1.1. The PPS method 3

1.2. Lattice doping 7

1.3. Interfacial segregation 21

1.4. Case study for dopant choices targeting high PEC performance on hematite 35

2. Experimental section 46

3. References 53

Each section further includes a dedicated summary detailing the figures and tables presented therein.

1. RESULTS AND DISCUSSION

1.1. The PPS method

SUMMARY:

- Discussion of the PPS method 3
- **Figure S1 –** DSC analysis of pristine and Step 1/Step 2-modified hematite polymers 5
- **Figure S2 –** XRD patterns of CuO and CeO_2 nanostructures synthesized through the PPS method 6

Following the idea of the traditional Pechini-based synthesis for oxides,[1] the polymeric precursor solution method is based on the formation of a polymeric network through an polyesterification reaction between a hydrocarboxylic acid (citric acid) coordinated to a metal cation (Cu^{2+}, Fe^{3+}, Ce^{4+}, *etc.*) and a polyhydroxy alcohol (ethylene glycol). Nonetheless, two modifications to this conventional sol-gel method were introduced to enhance the properties of the resulting polymer.

The first one consists in reducing the volume of the polymerized gel to 50% of its original volume by evaporating water at a temperature of 60-70 °C.[2] Given that esterification is a condensation reaction in which water is a byproduct,[3] the removal of H_2O through evaporation shifts the chemical equilibrium towards polymer formation, thereby ensuring complete solution polymerization. The second modification entails the addition of a mixture of ethanol and isopropyl alcohol to the polymerized gel to improve substrate wettability during film deposition and control film thickness.[4] The dilution of the polymeric precursor with this solvent mixture increases the polarity of the solution and induces a conformational rearrangement of the polymer chains from a predominantly linear to an entangled configuration, which improves molecular packing on rough surfaces (such as the FTO substrate).[5]

Applying hematite as a model system for this study, it is possible to see that the pristine polymer presents an amorphous nature, as evidenced by the presence of a glass transition in its differential scanning calorimetry (DSC) thermogram (given by the subtle change in slope at approximately 20 °C) without other discernible crystallization exotherms (Figure S1a). The incorporation of distinct modifiers during Step 1 of the PPS protocol (YH, LaH, AlH, and GaH, Figures S1b-e, respectively) leads to the emergence of a crystalline behavior, reflected by well-defined exothermic events. These changes can be given by the

intercalation of metal cations and iron in the polymer crystalline domains,[6-7] as well as ion-polymer chain complexation.[8] This behavior indicates effective dopant integration into the polymeric structure, which is expected to favor their distribution within the crystal lattice during thermal treatments.

Conversely, when modifiers are introduced during Step 2 of synthesis (HY, HLa, HAl, and HGa, Figures S1b-e, respectively), DSC thermograms show no significant deviations from the pristine polymer. This indicates that dopants are not incorporated into the polymeric chains but remain dispersed in the solution. Consequently, modifying cations are likely to accumulate at interfacial regions during annealing, rather than being integrated into the lattice structure.

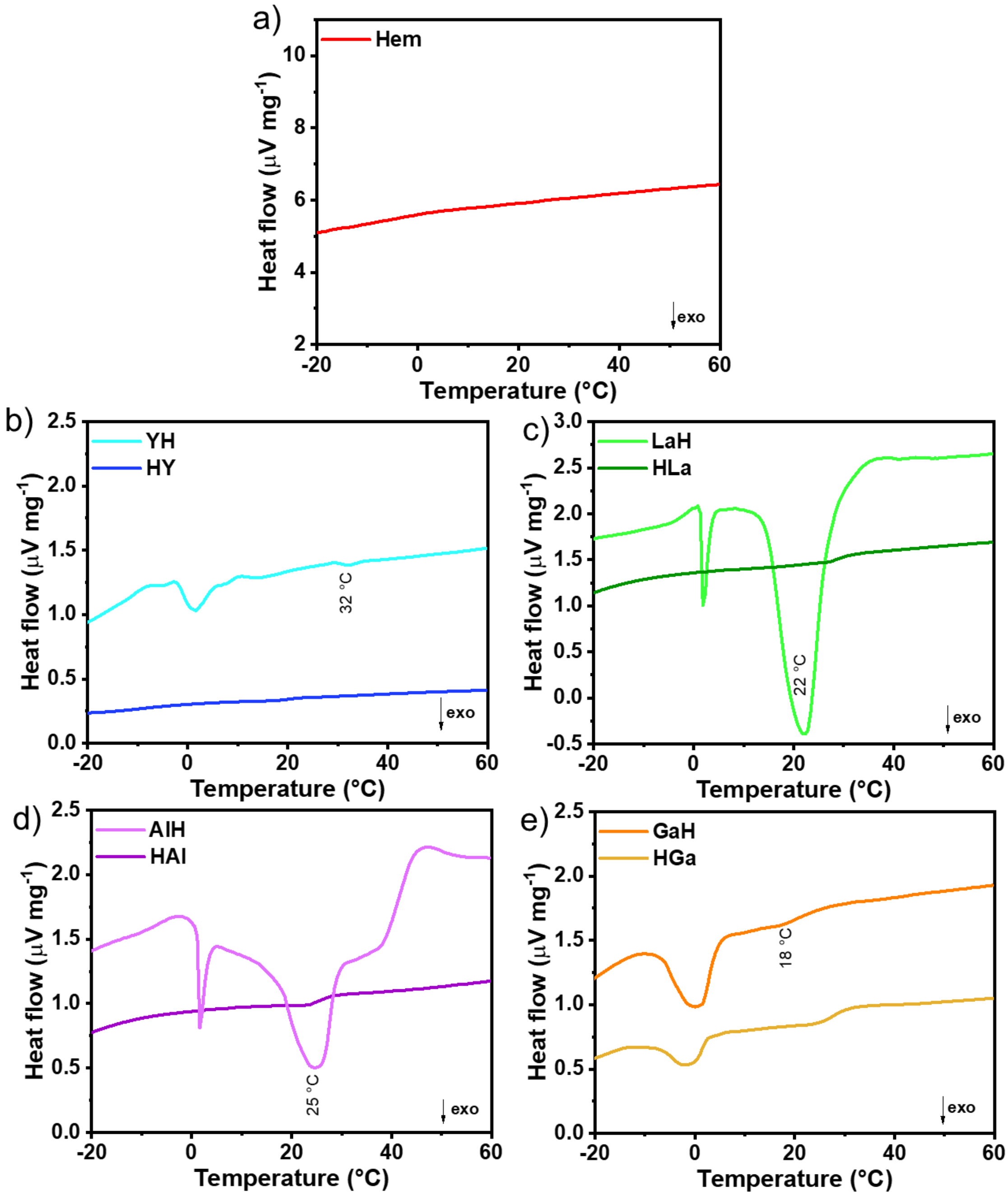

Figure S1. Differential scanning calorimetry (DSC) thermograms of a) pristine hematite polymeric precursor (Hem), b) yttrium-modified polymeric precursors (YH and HY), c) lanthanum-modified polymeric precursors (LaH and HLa), d) aluminum-modified polymeric precursors (AlH and HAl), and e) gallium-modified polymeric precursors (GaH and HGa).

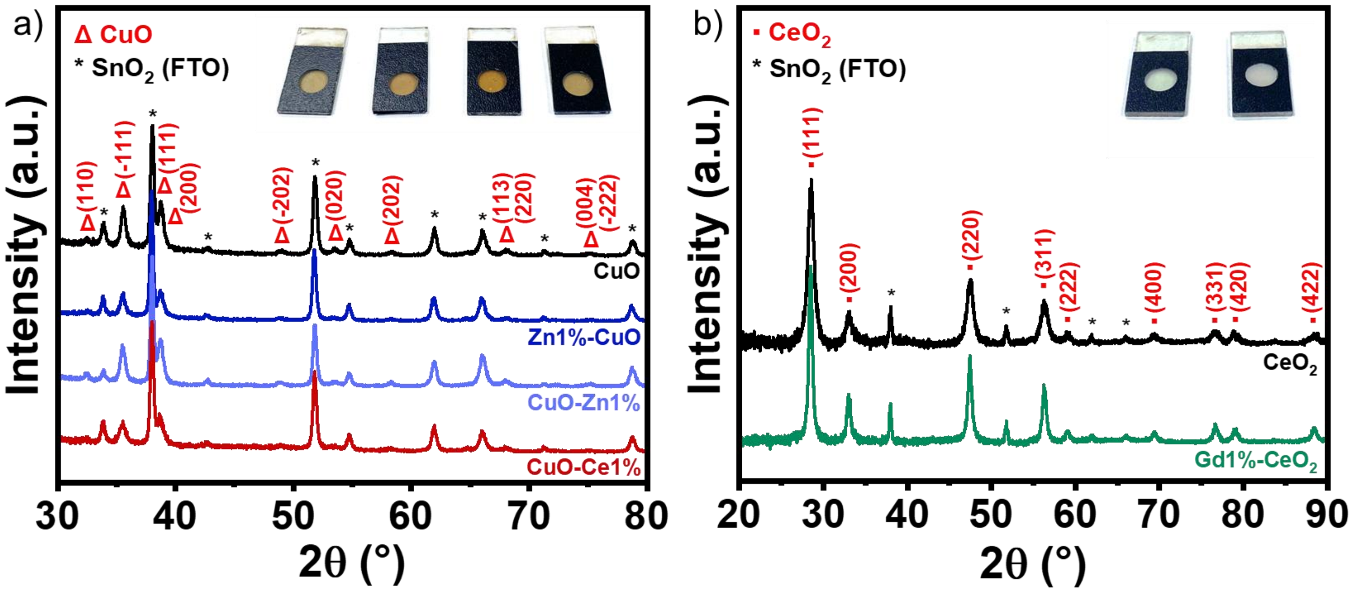

Figure S2. X-ray diffraction (XRD) patterns obtained for pristine and doped a) CuO and b) CeO_2 nanostructures produced through the PPS methodology. CuO signals (Δ) are assigned to JCPDS card no. 48-1548. CeO_2 signals (▪) are assigned to JCPDS card no. 34-0394. Other signals are assigned to the FTO conductive layer SnO_2 phase (*, JCPDS card no. 41-1445). The insets of the figures show pictures of synthesized CuO and CeO_2 photoanodes.

1.2. Lattice doping

SUMMARY:

- **Table S1 –** Chemical elements and precursor reagents used as hematite modifiers 8
- **Figure S3 –** XRD patterns of pristine and Step 1-modified hematite 9
- **Table S2 –** Cell parameters, Lotgering factor, and crystallite sizes of pristine and Step 1-modified hematite ... 9
- **Figure S4 –** FIB-SEM analysis of pristine and Step 1-modified hematite 10
- **Figure S5 –** Cyclic voltammetry of pristine and Step 1-modified hematite 11
- **Figure S6 –** Average *j vs.* scan rate for pristine and Step 1-modified hematite 12
- **Table S3 –** ECSA values of pristine and Step 1-modified hematite 12
- **Figure S7 –** AFM analysis of pristine and Step 1-modified hematite 13
- **Table S4 –** Mean roughness and RMS values of pristine and Step 1-modified hematite 13
- **Figure S8 –** Absorbance spectra, Tauc plots, and J_{abs} values for pristine and Step 1-modified hematite 14
- **Figure S9 –** Fe 2p XPS spectra of pristine and Step 1-modified hematite 15
- **Figure S10 –** XPS spectra of Y 3d, La 3d, Al 2p, and Ga 2p for Step 1-modified photoanodes 16
- **Figure S11 –** Mott-Schottky plots of pristine and Step 1-modified hematite 17
- **Table S5 –** Calculated charge donor densities for pristine and Step 1-modified hematite 18
- **Figure S12 –** Raman spectra of pristine and Step 1-modified hematite 19
- **Table S6 –** Raman spectral deconvolution for pristine and Step 1-modified hematite 20

Table S1. Screening of 18 chemical elements employed in this work as hematite modifiers. Yttrium, lanthanum, aluminum and gallium were selected for an in-depth study and employed as modifiers both at Step 1 and Step 2 of the PPS protocol. The precursor salts were incorporated into the polymeric solution as powders along with Fe^{3+} when added at Step 1, and in ethanol solutions at Step 2.

Group (Periodic Table)	Modifying ion	Precursor	Addition at Step 1	Addition at Step 2
**1**	Na^{+}	$NaNO_3$, ≥99%, Sigma-Aldrich		HNa
**2**	Sr^{2+}	$Sr(NO_3)_2$, ≥99%, Alfa Aesar		HSr
**3**	Y^{3+}	$Y(NO_3)_3.6H_2O$, 99.8%, Sigma-Aldrich	YH	HY
	La^{3+}	$La(NO_3)_3.6H_2O$, 99.999%, Sigma-Aldrich	LaH	HLa
**4**	Ti^{4+}	$C_{16}H_{36}O_4Ti$, 97%, Sigma-Aldrich		HTi
	Zr^{4+}	$ZrO(NO_3)_2.xH_2O$, 99%, Sigma-Aldrich		HZr
	Hf^{4+}	$HfCl_4$, 98%, Sigma-Aldrich		HHf
**5**	Nb^{5+}	$NbCl_5$, ≥99.9%, Sigma-Aldrich		HNb
	Ta^{5+}	$TaCl_5$, 99.99%, Sigma-Aldrich		HTa
**7**	Mn^{2+}	$Mn(NO_3)_2.4H_2O$, ≥97%, Sigma-Aldrich		HMn
**10**	Ni^{2+}	$Ni(NO_3)_2.6H_2O$, ≥97%, Sigma-Aldrich		HNi
**12**	Zn^{2+}	$Zn(NO_3)_2.6H_2O$, 99%, Alfa Aesar		HZn
**13**	Al^{3+}	$Al(NO_3)_3.9H_2O$, 99%, Sigma-Aldrich	AlH	HAl
	Ga^{3+}	$Ga(NO_3)_3.xH_2O$, 99.9%, Alfa Aesar	GaH	HGa
**14**	Si^{4+}	$SiC_8H_{20}O_4$, 99.999+%, Alfa Aesar		HSi
	Ge^{4+}	$Ge(C_2H_5O)_4$, 99.995%, Alfa Aesar		HGe
	Sn^{4+}	$SnCl_4.5H_2O$, 98%, Sigma-Aldrich		HSn
**15**	Sb^{5+}	$SbCl_3$, ≥99%, Sigma-Aldrich		HSb

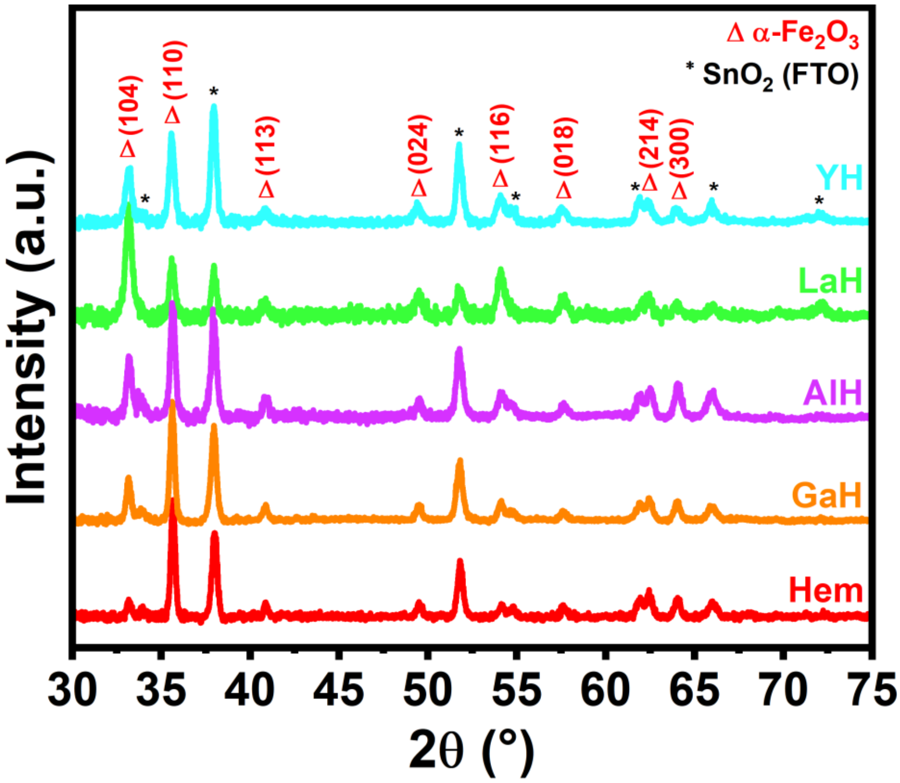

Figure S3. X-ray diffraction (XRD) patterns obtained for pristine hematite (Hem), YH, LaH, AlH, and GaH photoanodes. α-Fe_2O_3 signals (Δ) are assigned to JCPDS card no. 33-0664. Other signals are assigned to the FTO conductive layer SnO_2 phase (*, JCPDS card no. 41-1445).

Table S2. Cell parameters, Lotgering factor (110 plane) and crystallite sizes (110, 104 and 300 planes) calculated from XRD analysis for pristine hematite (Hem), YH, LaH, AlH, and GaH photoanodes.

Photoanode	Cell parameters			Lotgering factor (F) (1 1 0)	Crystallite size (nm) (1 1 0)	Crystallite size (nm) (1 0 4)	Crystallite size (nm) (3 0 0)
	a (Å)	*c* (Å)	V (Å^3)				
**Hem**	4.93	13.47	284	0.71	28.3	26.5	25.2
**YH**	4.94	13.49	285	0.49	22.1	19.2	17.5
**LaH**	4.94	13.46	284	0.11	21.7	17.6	18.2
**AlH**	4.93	13.47	284	0.49	23.6	22.9	21.6
**GaH**	4.93	13.48	284	0.69	26.3	25.4	23.0

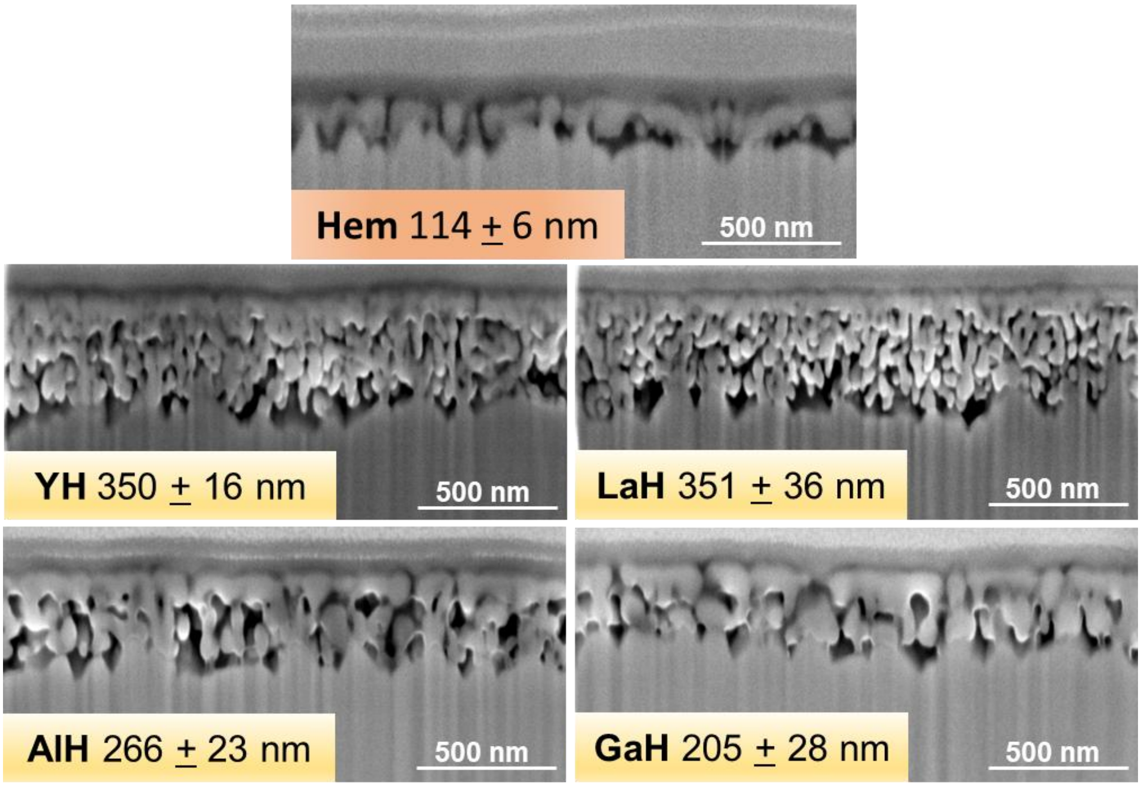

Figure S4. Cross-section micrographs obtained from focused ion beam analysis in configuration with scanning electron microscopy (FIB-SEM) for pristine hematite (Hem) and lattice-doped materials (addition of dopants during Step 1 of the PPS protocol – YH, LaH, AlH and GaH).

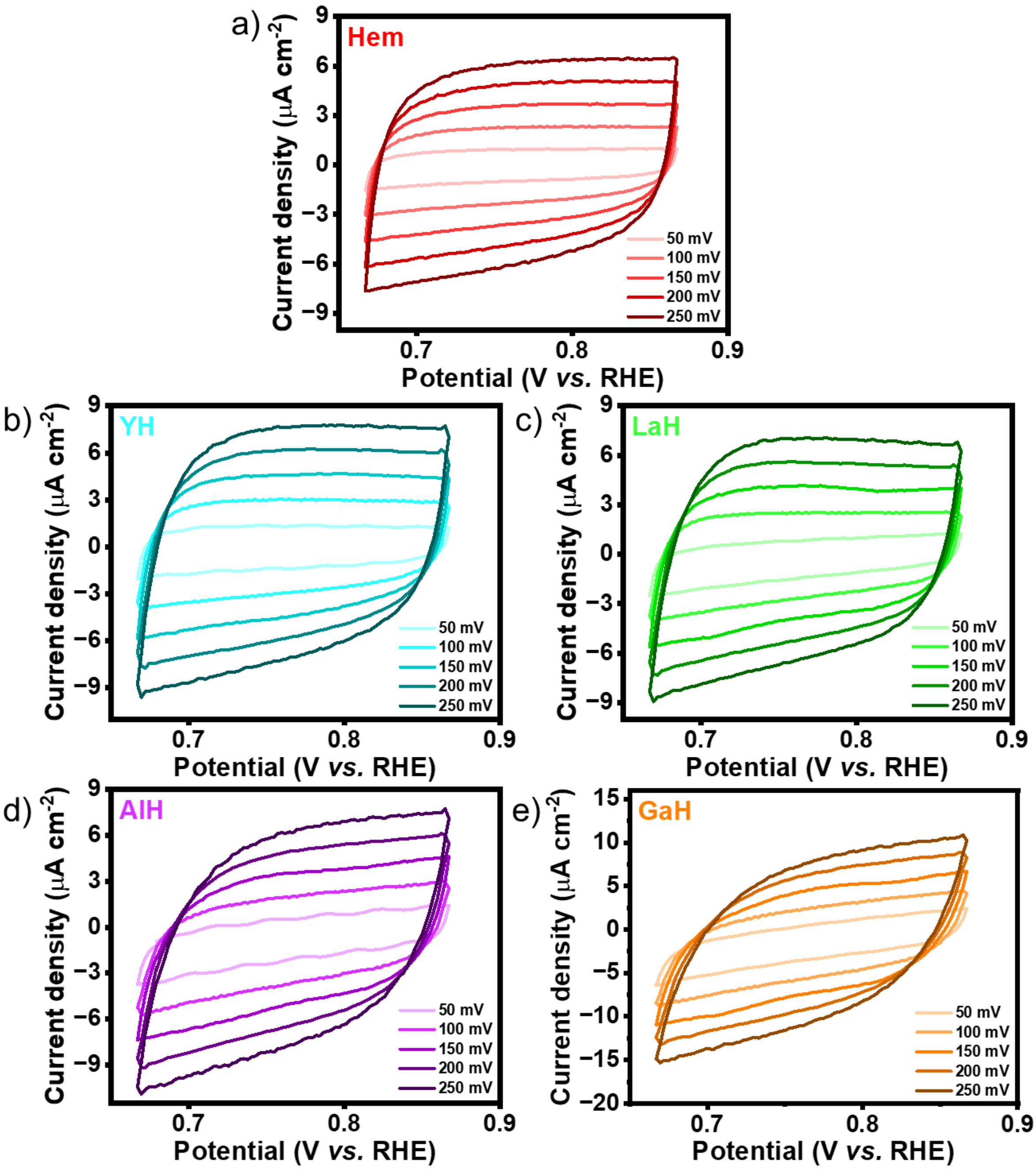

Figure S5. Cyclic voltammograms obtained under dark conditions for a) pristine hematite (Hem), b) YH, c) LaH, d) AlH, and e) GaH. Scan rates of 50 mV, 100 mV, 150 mV, 200 mV and 250 mV were employed to conduct the measurements.

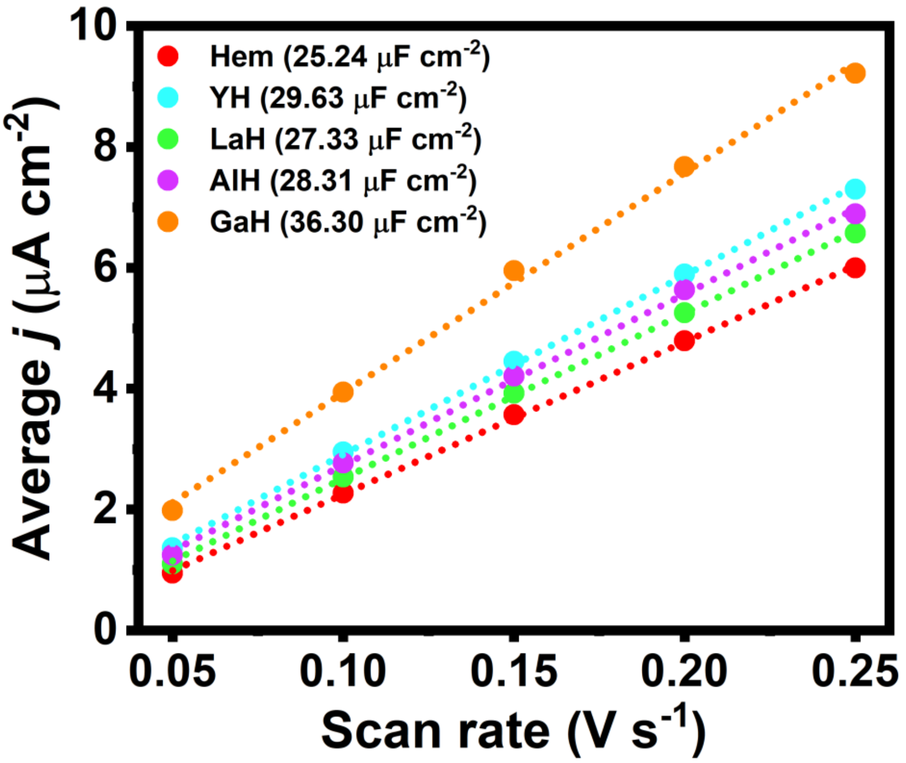

Figure S6. Average *j versus* scan rate plot constructed from cyclic voltammetry measurements for pristine hematite (Hem) and lattice-doped materials (addition of dopants during Step 1 of the PPS protocol – YH, LaH, AlH and GaH). The slope of the curve represents the double layer capacitance (C_{DL}) of the materials, with calculated values being represented in parentheses.

Table S3. Estimated electrochemically active surface area (ECSA) values for pristine hematite (Hem) and lattice-doped materials (addition of dopants during Step 1 of the PPS protocol – YH, LaH, AlH and GaH). Data was obtained from cyclic voltammetry experiments.

Photoanode	ECSA
**Hem**	0.63
**YH**	0.74
**LaH**	0.68
**AlH**	0.71
**GaH**	0.91

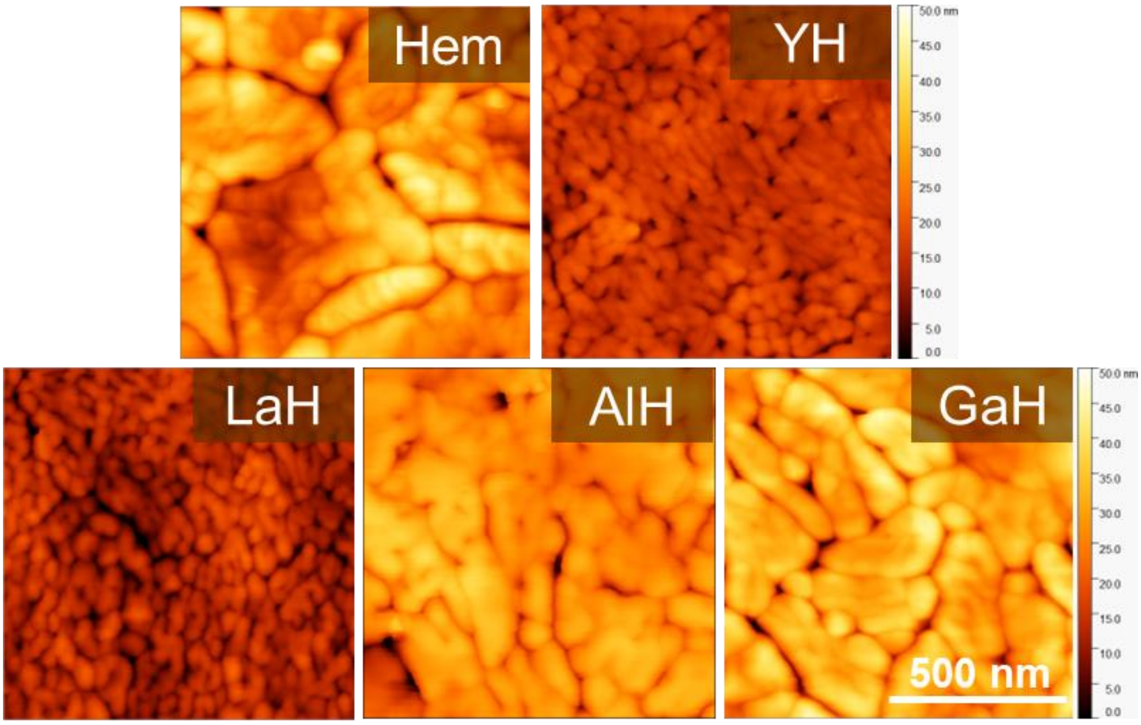

Figure S7. Topography maps of pristine hematite (Hem), YH, LaH, AlH and GaH photoanodes obtained by atomic force microscopy (AFM) technique. All hematite-based photoanodes were modified with 1% molar ratio (element:Fe^{3+}) of dopants. Modifiers were added during Step 1 of the PPS protocol.

Table S4. Mean value and mean square roughness (RMS) of height irregularities calculated from atomic force microscopy images of pristine hematite (Hem) and photoanodes modified during Step 1 (YH, LaH, AlH, GaH) of the PPS protocol.

Photoanode	Mean value (nm)	RMS (nm)
**Hem**	45.1	9.6
**YH**	18.2	5.1
**LaH**	17.8	4.4
**AlH**	24.8	5.9
**GaH**	32.2	7.0

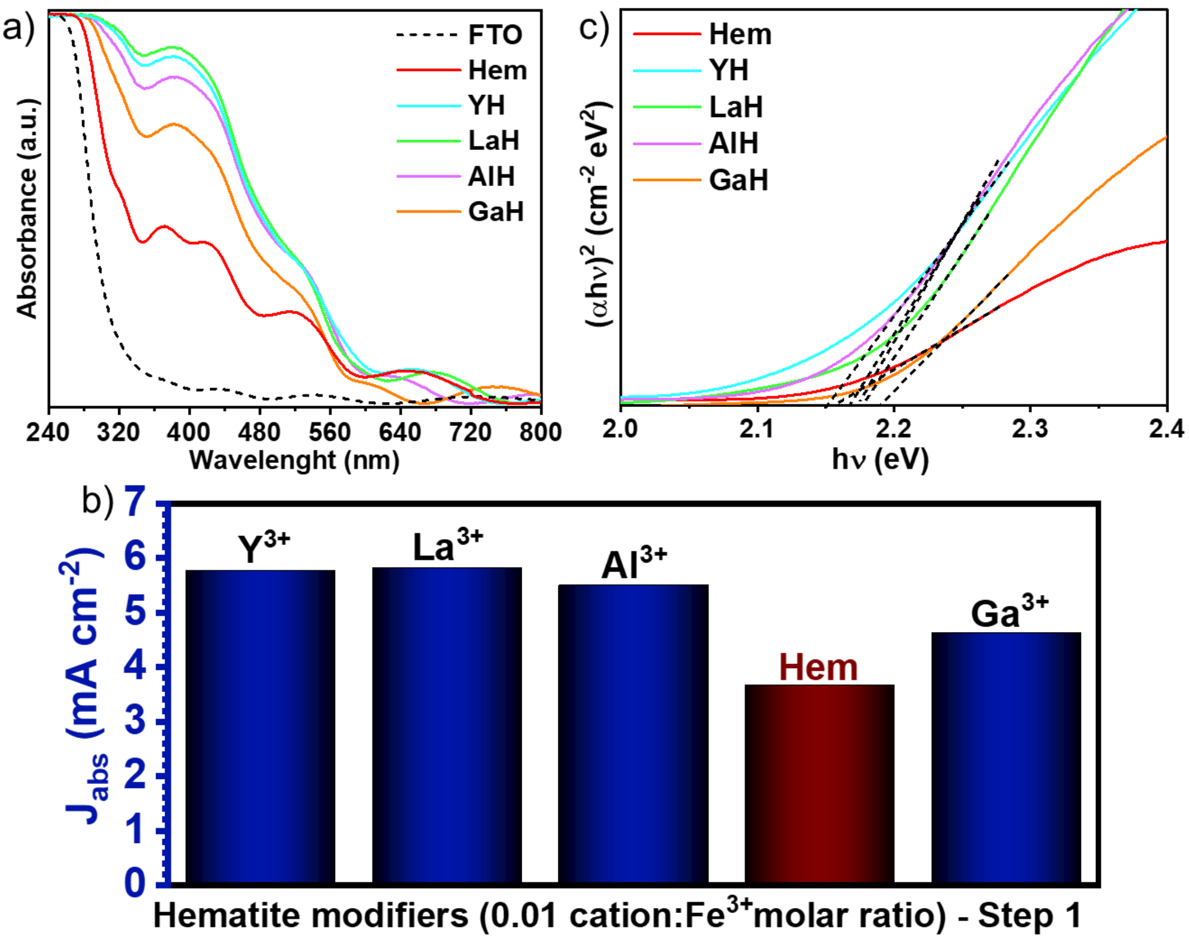

Figure S8. a) Absorbance spectra of pristine hematite (Hem) and photoanodes modified during Step 1 (MH) of the PPS protocol. b) Bar chart of the maximum experimental current due to the absorption properties of pristine hematite and photoanodes modified during Step 1 (MH) of the PPS protocol (J_{abs}). c) Tauc plots for direct band-gap determination of pristine hematite (Hem) and photoanodes modified during Step 1 (MH) of the PPS protocol. Dashed lines represent the linear fit of the curve's linear regions.

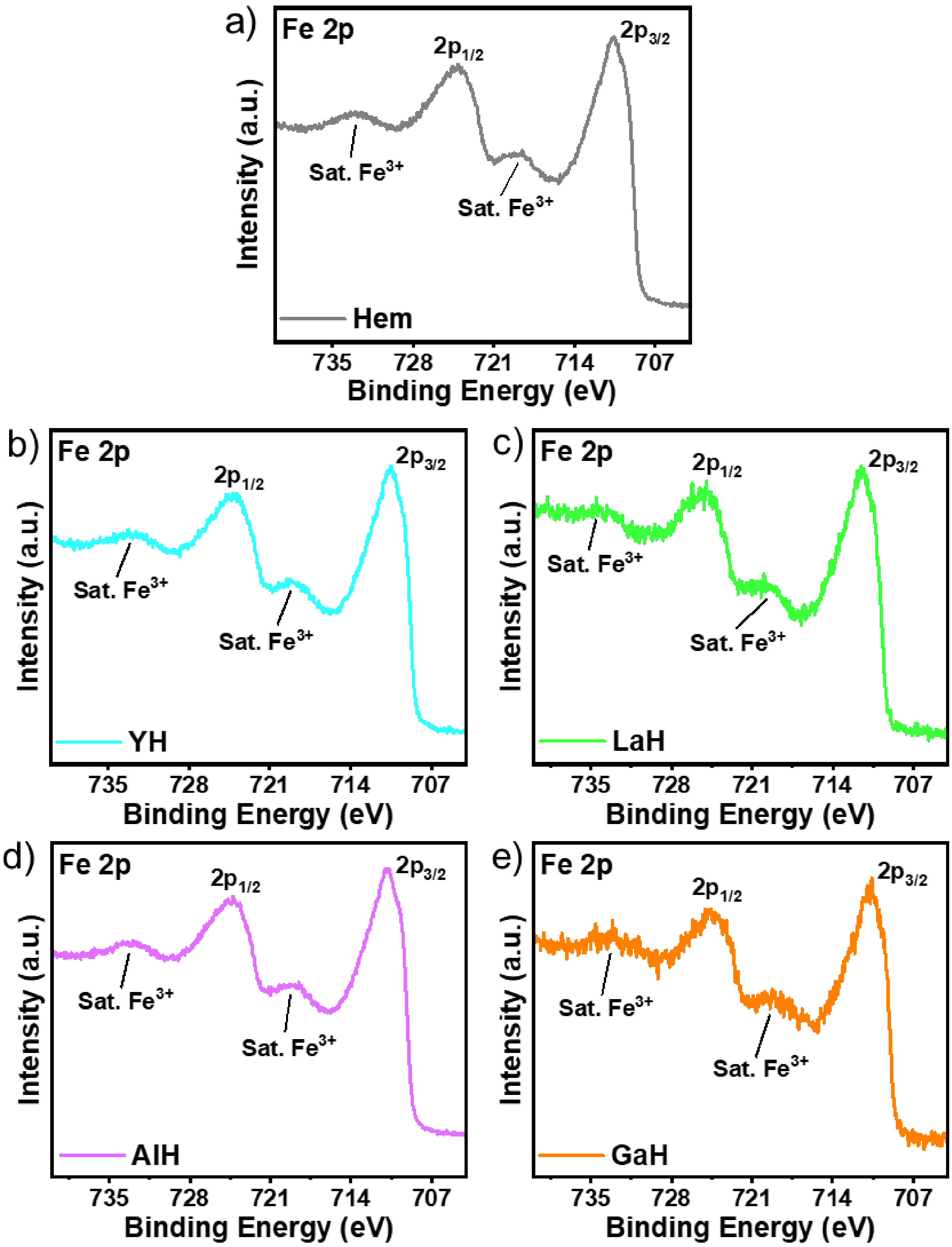

Figure S9. Deconvoluted high-resolution XPS spectra of Fe 2p for a) pristine hematite (Hem), b) YH, c) LaH, d) AlH, and e) GaH.

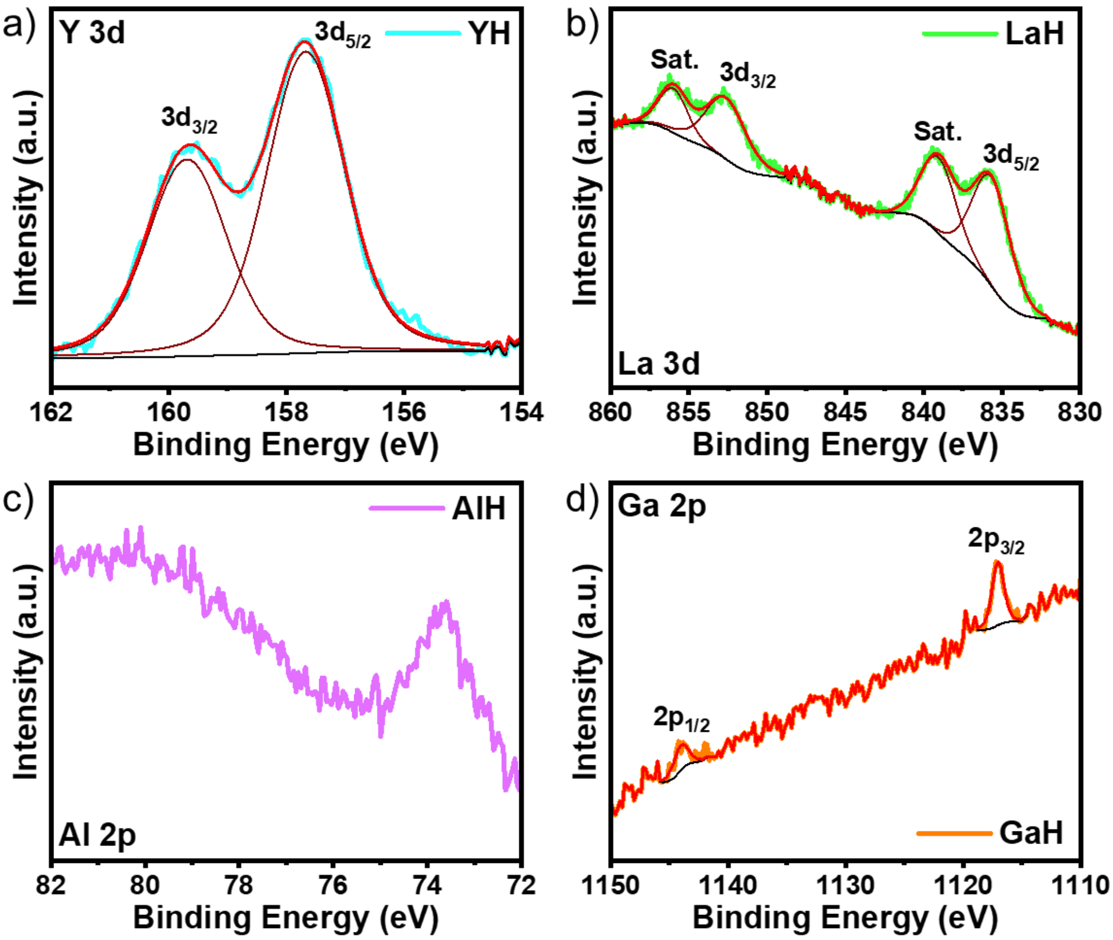

Figure S10. Deconvoluted high-resolution XPS spectra of a) Y 3d from YH, b) La 3d from LaH, c) Al 2p from AlH, and d) Ga 2p from GaH.

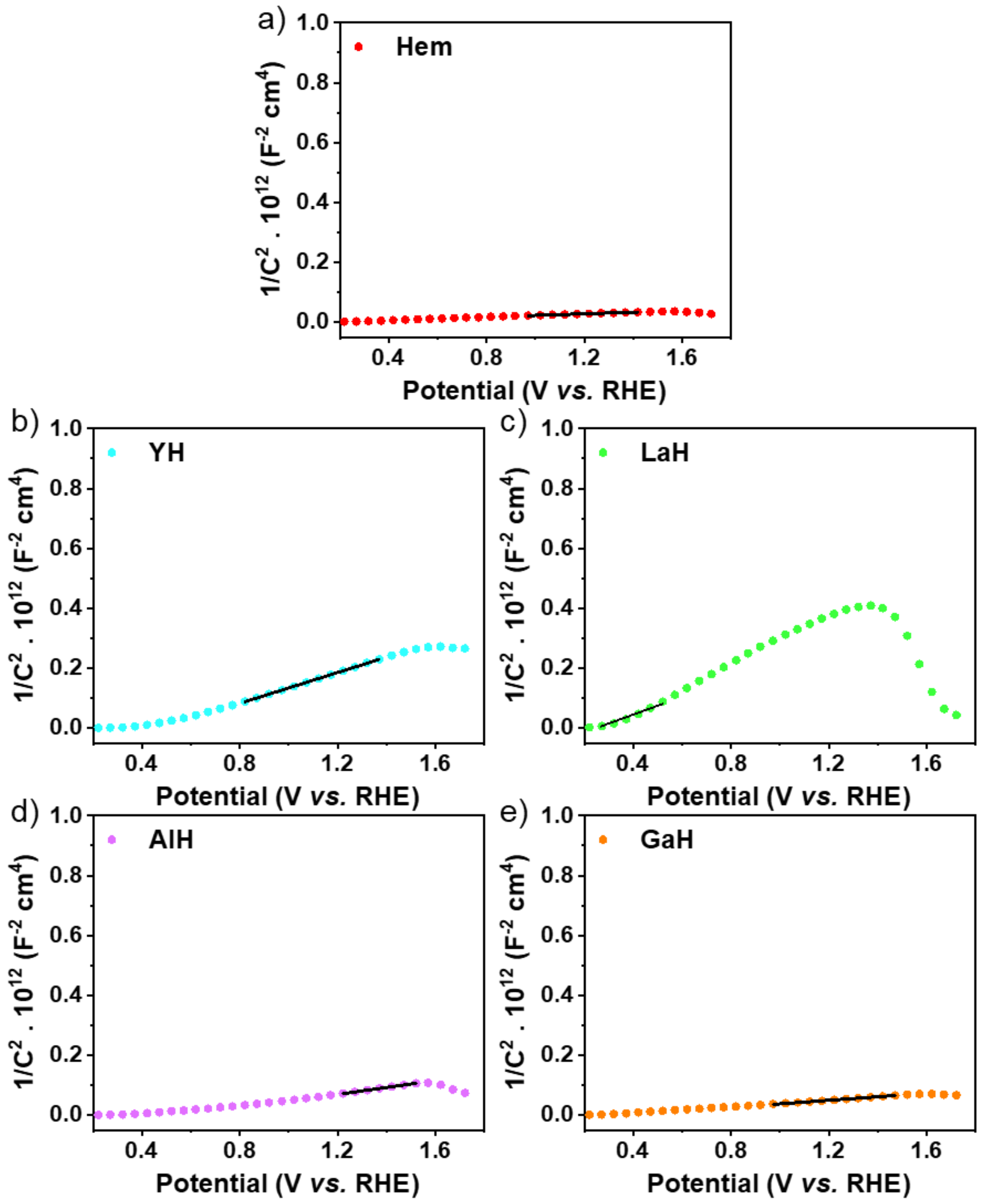

Figure S11. Mott-Schottky analysis of a) pristine hematite (Hem), b) YH, c) LaH, d) AlH, and e) GaH photoanodes. Data was extracted from electrochemical impedance spectroscopy (EIS) at the frequency of 1 kHz.

Table S5. Calculated charge donor density (N_D) for pristine hematite (Hem), YH, LaH, AlH and GaH photoanodes. Data extracted from Mott-Schottky analysis.

Photoanode	N_D (cm^{-3})
**Hem**	10^{20}
**YH**	10^{19}
**LaH**	10^{20}
**AlH**	10^{20}
**GaH**	10^{20}

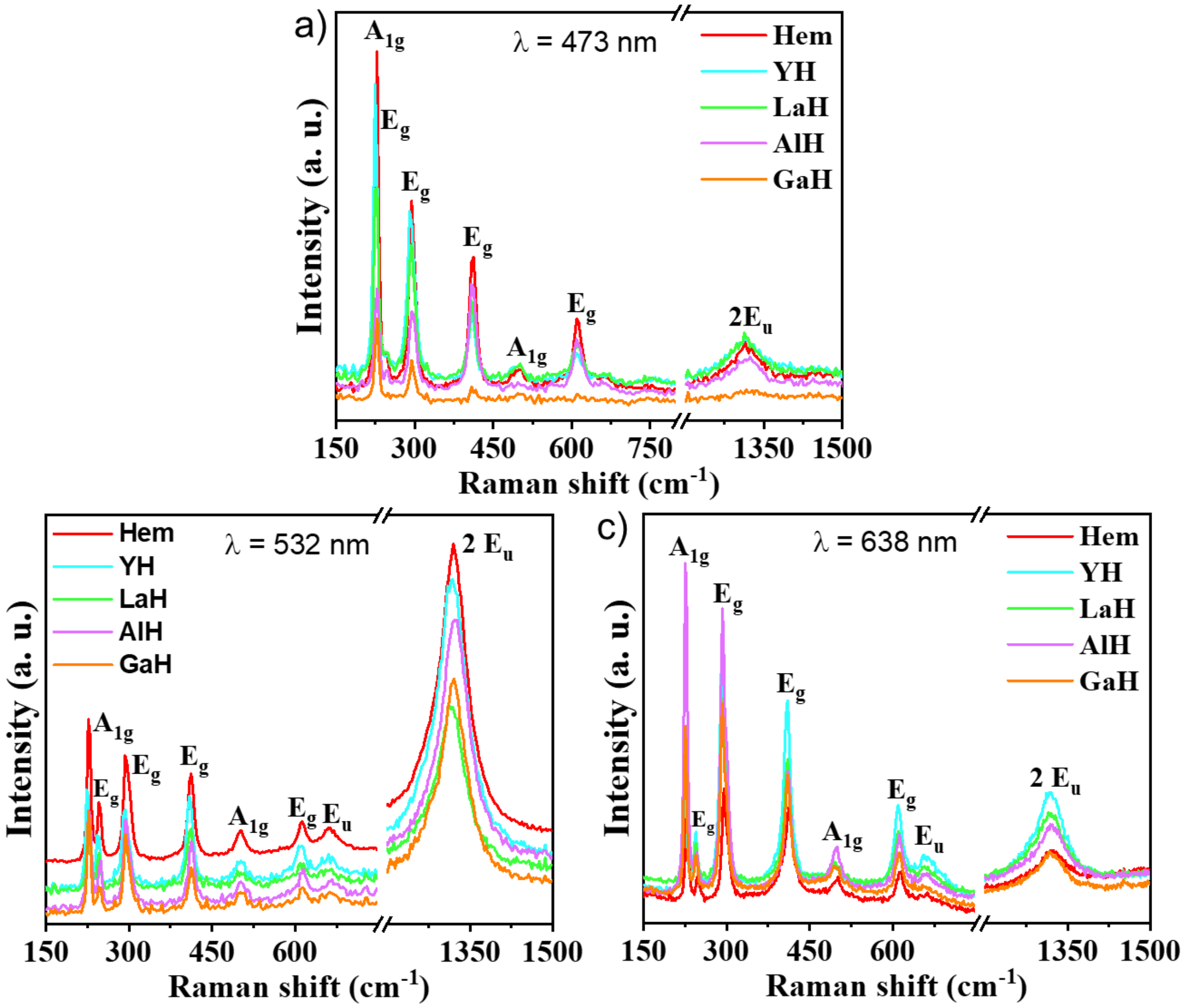

Figure S12. Raman spectra obtained for pristine hematite (Hem), YH, LaH, AlH and GaH photoanodes acquired through the irradiation of lasers with wavelength of a) 473 nm (blue), b) 532 nm (green), and c) 638 nm (red).

Table S6. Calculated full width at half maximum (FWHM) of the E_u peak at 660 cm^{-1} obtained from Raman spectroscopy analysis at λ = 532 nm for pristine hematite (Hem), YH, LaH, AlH and GaH photoanodes.

Photoanode	FWHM E_u peak (660 cm^{-1})
	**λ = 532 nm**
**Hem**	37
**YH**	43
**LaH**	41
**AlH**	49
**GaH**	43

1.3. Interfacial segregation

SUMMARY:

- **Figure S13 –** XRD patterns of pristine and Step 2-modified hematite 22
- **Table S7 –** Cell parameters, Lotgering factor, and crystallite sizes of pristine and Step 2-modified hematite ... 22
- **Figure S14 –** FIB-SEM analysis of pristine and Step 2-modified hematite 23
- **Figure S15 –** Cyclic voltammetry of pristine and Step 2-modified hematite 24
- **Figure S16 –** Average *j vs.* scan rate for pristine and Step 2-modified hematite 25
- **Table S8 –** ECSA values of pristine and Step 2-modified hematite 25
- **Figure S17 –** AFM analysis of pristine and Step 2-modified hematite 26
- **Table S9 –** Mean roughness and RMS values of pristine and Step 2-modified hematite 26
- **Figure S18 –** Absorbance spectra, Tauc plots, and J_{abs} values for pristine and Step 2-modified hematite 27
- **Figure S19 –** Fe 2p XPS spectra of pristine and Step 2-modified hematite 28
- **Figure S20 –** XPS spectra of Y 3d, La 3d, Al 2p, and Ga 2p for Step 2-modified photoanodes 29
- **Figure S21 –** Mott-Schottky plots of pristine and Step 2-modified hematite 30
- **Table S10 –** Calculated charge donor densities for pristine and Step 2-modified hematite 31
- **Figure S22 –** Raman spectra of pristine and Step 2-modified hematite 32
- **Table S11 –** Raman spectral deconvolution for pristine and Step 2-modified hematite 33
- **Figure S23 –** Photocurrent density profiles of multiple hematite photoanodes modified during Step 2 34

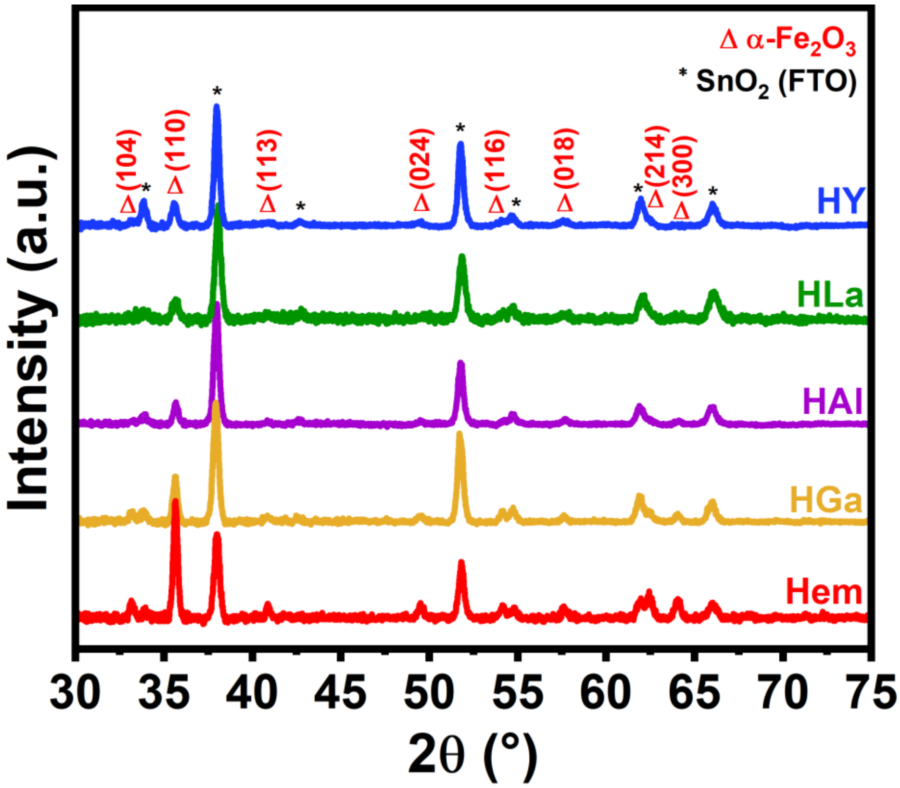

Figure S13. X-ray diffraction (XRD) patterns obtained for pristine hematite (Hem), HY, HLa, HAl and HGa photoanodes. α-Fe_2O_3 signals (Δ) are assigned to JCPDS card no. 33-0664. Other signals are assigned to the FTO conductive layer SnO_2 phase (*, JCPDS card no. 41-1445).

Table S7. Cell parameters, Lotgering factor (110 plane) and crystallite sizes (110, 104 and 300 planes) calculated from XRD analysis for pristine hematite (Hem), HY, HLa, HAl and HGa photoanodes.

Photoanode	Cell parameters			Lotgering factor (F) (1 1 0)	Crystallite size (nm) (1 1 0)	Crystallite size (nm) (1 0 4)	Crystallite size (nm) (3 0 0)
	a (Å)	*c* (Å)	V (Å^3)				
**Hem**	4.93	13.47	284	0.71	28.3	26.5	25.2
**HY**	4.93	13.48	284	0.90	23.2	-	-
**HLa**	4.94	13.49	285	1.00	19.8	-	-
**HAl**	4.93	13.47	284	0.80	24.4	-	19.6
**HGa**	4.93	13.47	284	0.76	26.1	23.3	22.7

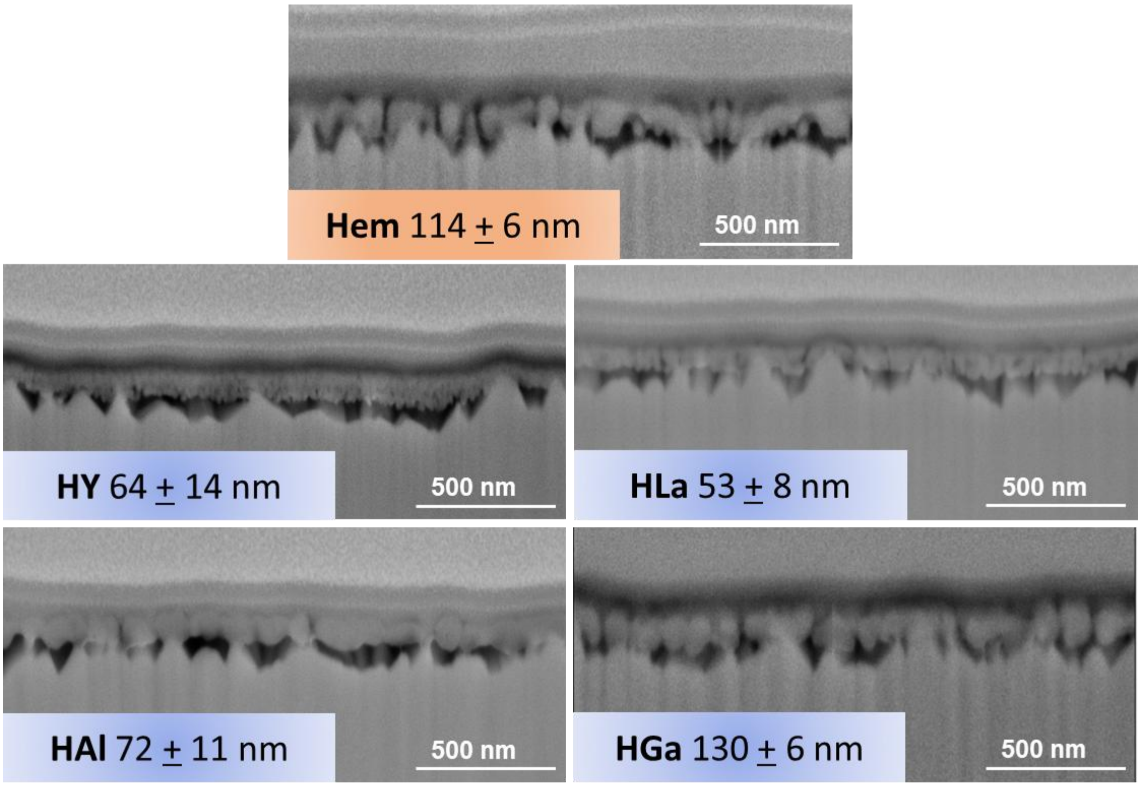

Figure S14. Cross-section micrographs obtained from focused ion beam analysis in configuration with scanning electron microscopy (FIB-SEM) for pristine hematite (Hem) and interfacially modified materials (addition of dopants during Step 2 of the PPS protocol – HY, HLa, HAl and HGa).

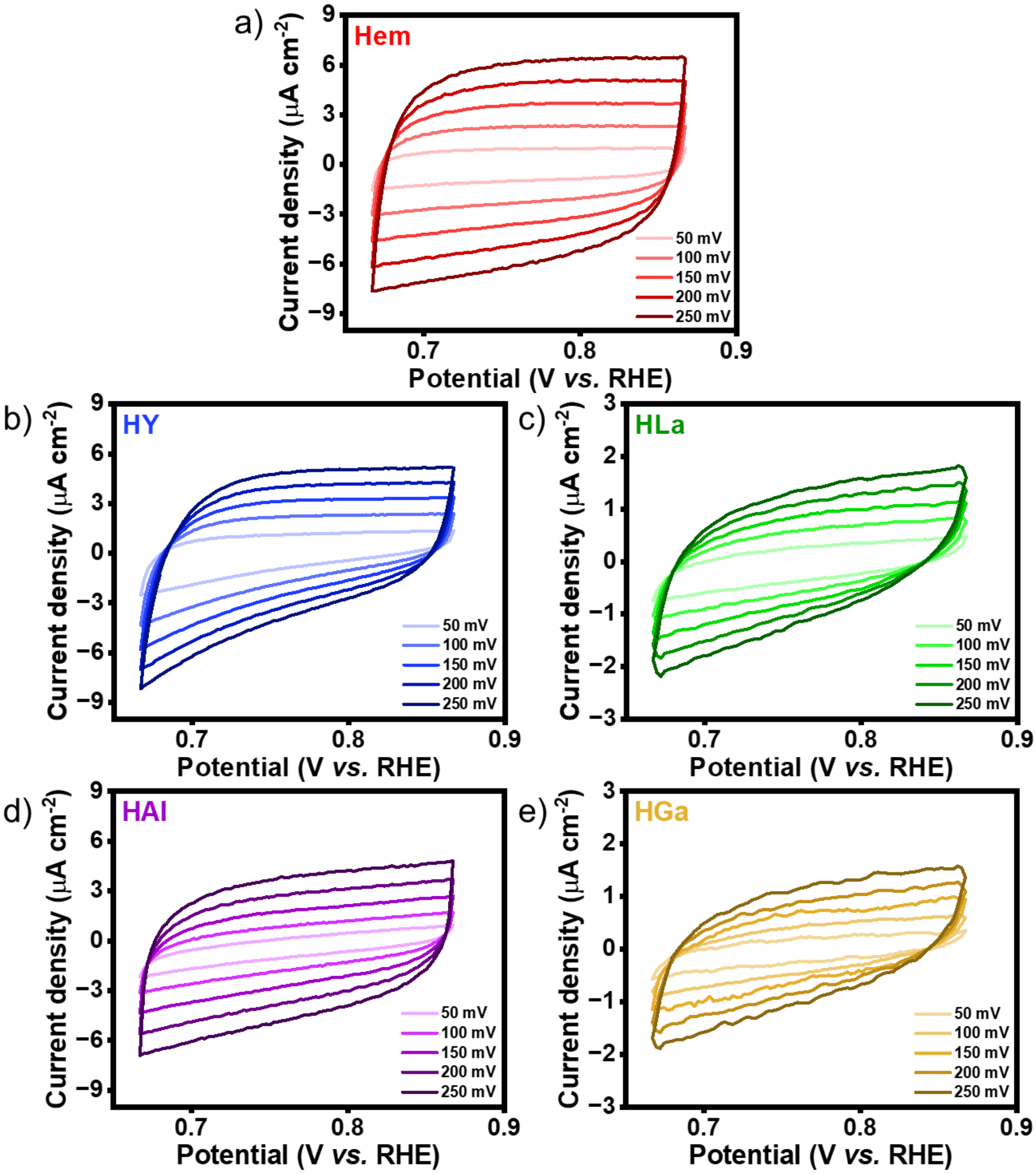

Figure S15. Cyclic voltammograms obtained under dark conditions for a) pristine hematite (Hem), b) HY, c) HLa, d) HAl, and e) HGa. Scan rates of 50 mV, 100 mV, 150 mV, 200 mV and 250 mV were employed to conduct the measurements.

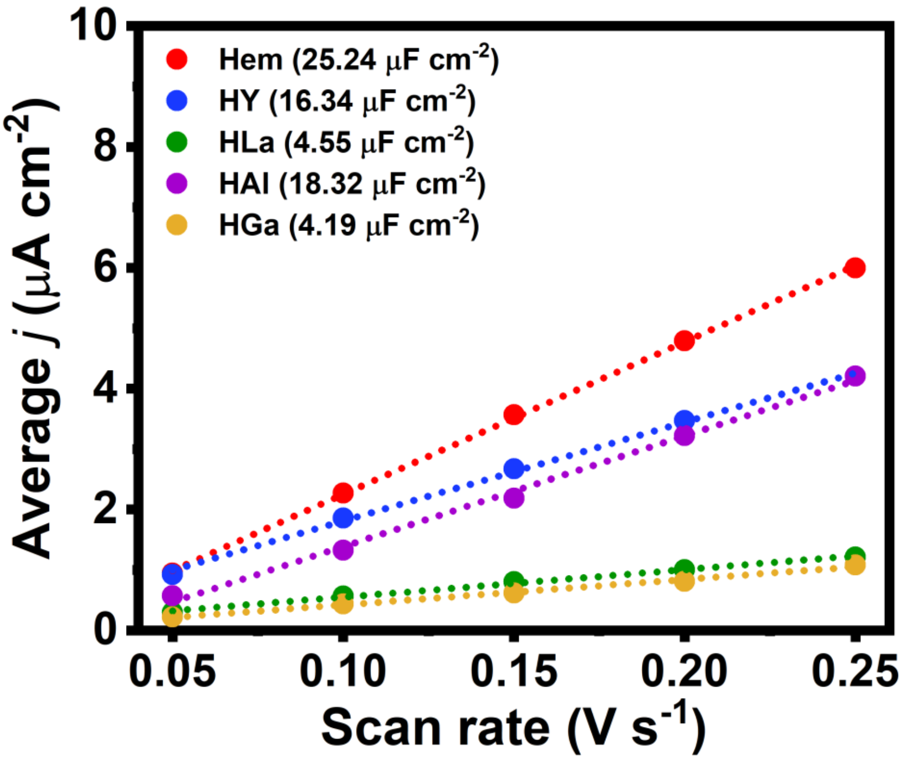

Figure S16. Average *j versus* scan rate plot constructed from cyclic voltammetry measurements for pristine hematite (Hem) and interfacially modified materials (addition of dopants during Step 2 of the PPS protocol – HY, HLa, HAl and HGa). The slope of the curve represents the double layer capacitance (C_{DL}) of the materials, with calculated values being represented in parentheses.

Table S8. Estimated electrochemically active surface area (ECSA) values for pristine hematite (Hem) and interfacially modified materials (addition of dopants during Step 2 of the PPS protocol – HY, HLa, HAl and HGa). Data was obtained from cyclic voltammetry experiments.

Photoanode	ECSA
**Hem**	0.63
**YH**	0.41
**LaH**	0.11
**AlH**	0.46
**GaH**	0.11

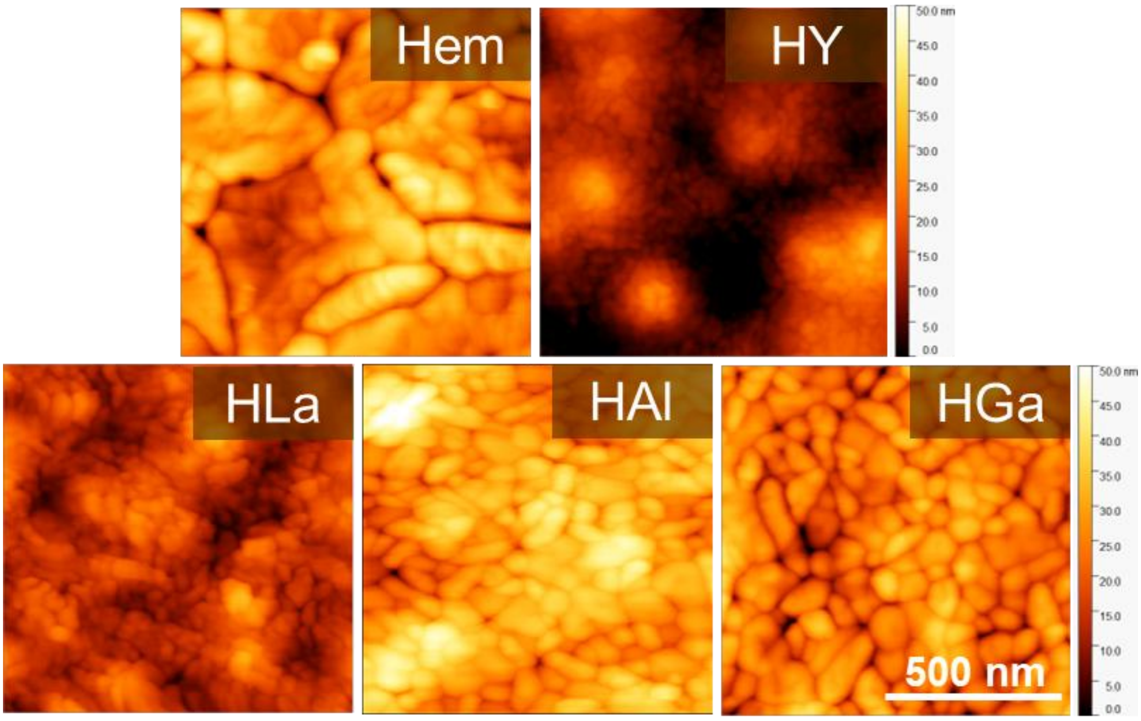

Figure S17. Topography maps of pristine hematite (Hem), HY, HLa, HAl and HGa photoanodes obtained by atomic force microscopy (AFM) technique. All hematite-based photoanodes were modified with 1% molar ratio (element:Fe^{3+}) of dopants. Modifiers were added during Step 2 of the PPS protocol.

Table S9. Mean value and mean square roughness (RMS) of height irregularities calculated from atomic force microscopy images of pristine hematite (Hem) and photoanodes modified during Step 2 (HY, HLa, HAl, HGa) of the PPS protocol.

Photoanode	Mean value (nm)	RMS (nm)
**Hem**	45.1	9.6
**HY**	15.3	3.7
**HLa**	15.3	3.7
**HAl**	18.1	5.0
**HGa**	26.1	6.1

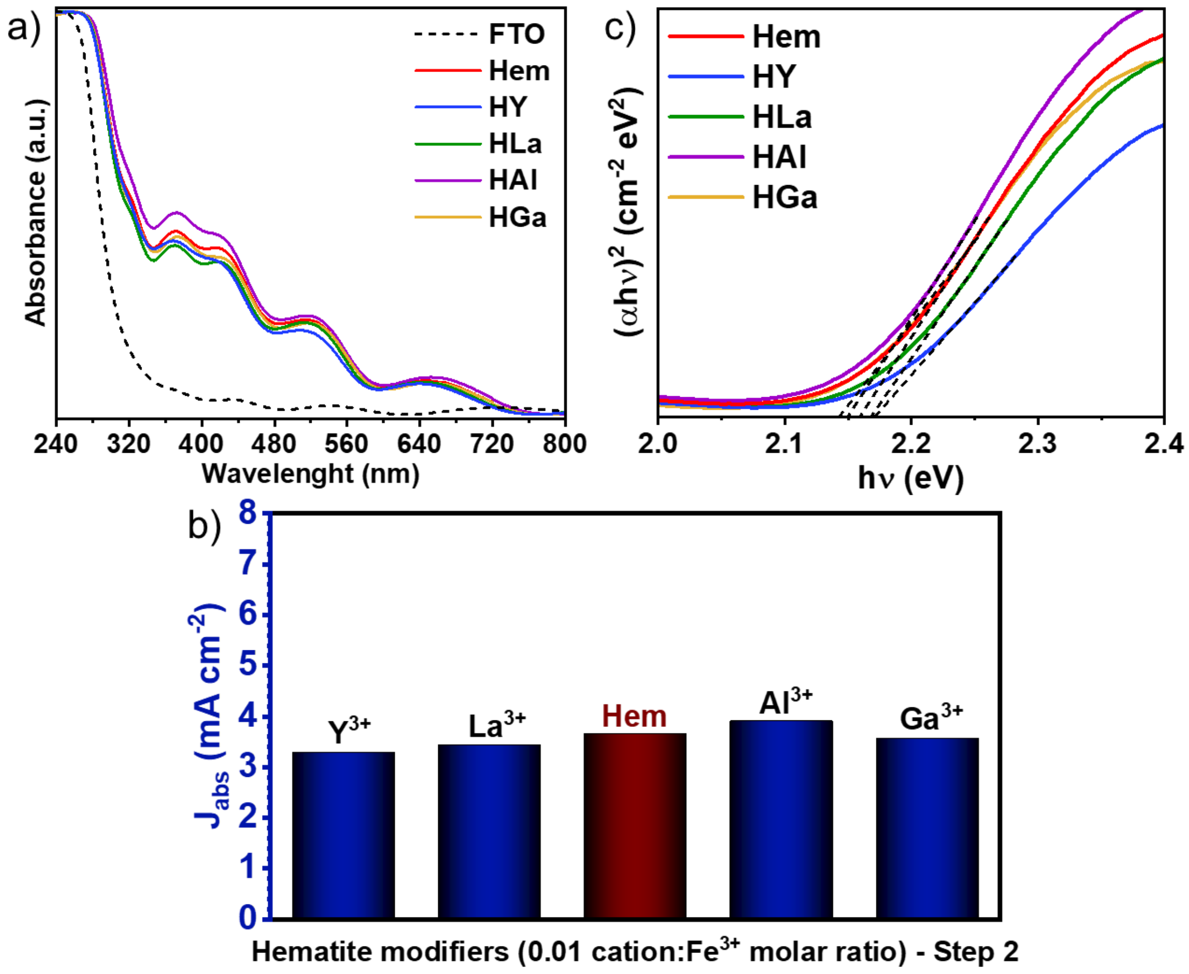

Figure S18. a) Absorbance spectra of pristine hematite (Hem) and photoanodes modified during Step 2 (HM) of the PPS protocol. b) Bar chart of the maximum experimental current due to the absorption properties of pristine hematite and photoanodes modified during Step 2 (HM) of the PPS protocol (J_{abs}). c) Tauc plots for direct band-gap determination of pristine hematite (Hem) and photoanodes modified during Step 2 (HM) of the PPS protocol. Dashed lines represent the linear fit of the curve's linear regions.

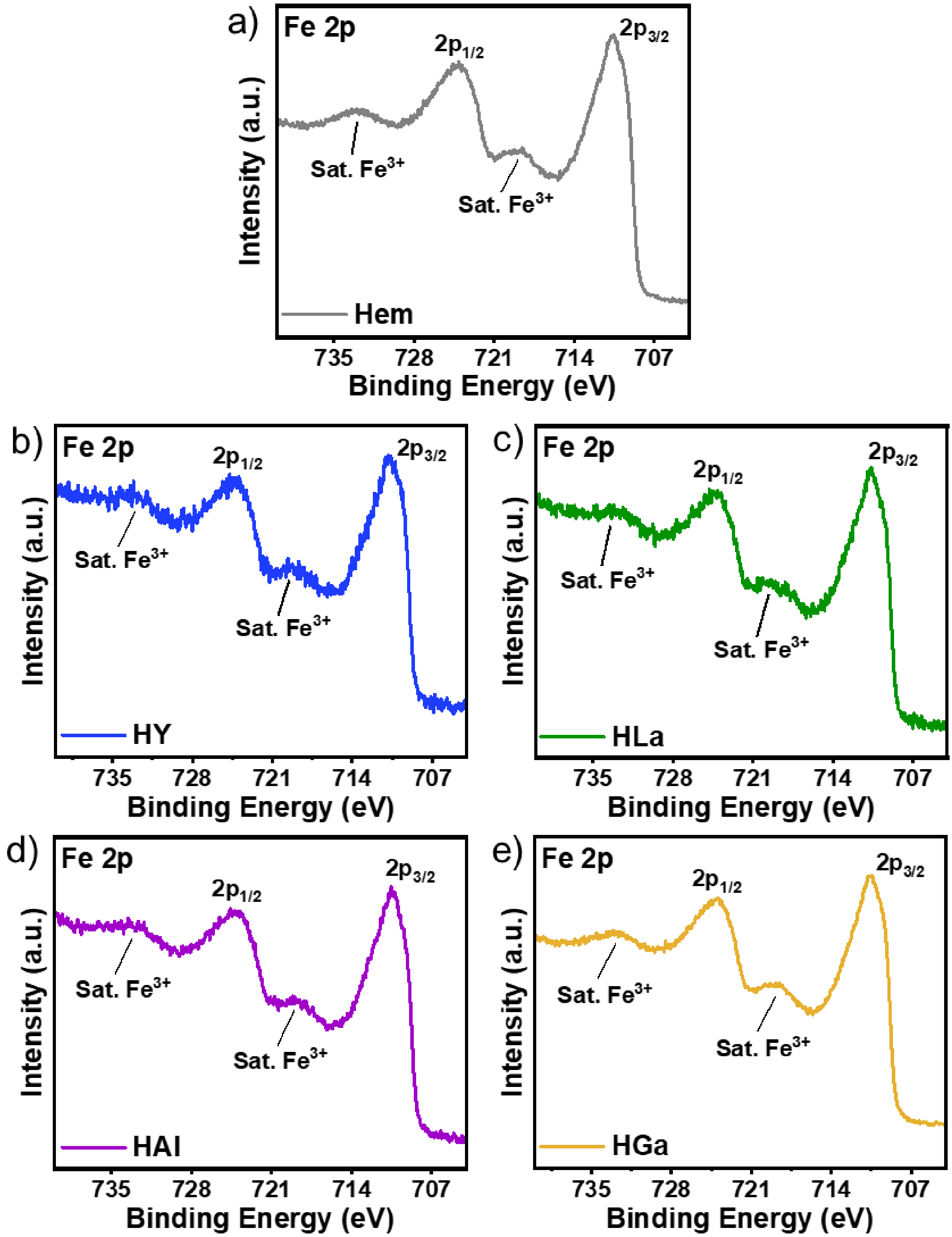

Figure S19. Deconvoluted high-resolution XPS spectra of Fe 2p for a) pristine hematite (Hem), b) HY, c) HLa, d) HAl, and e) HGa.

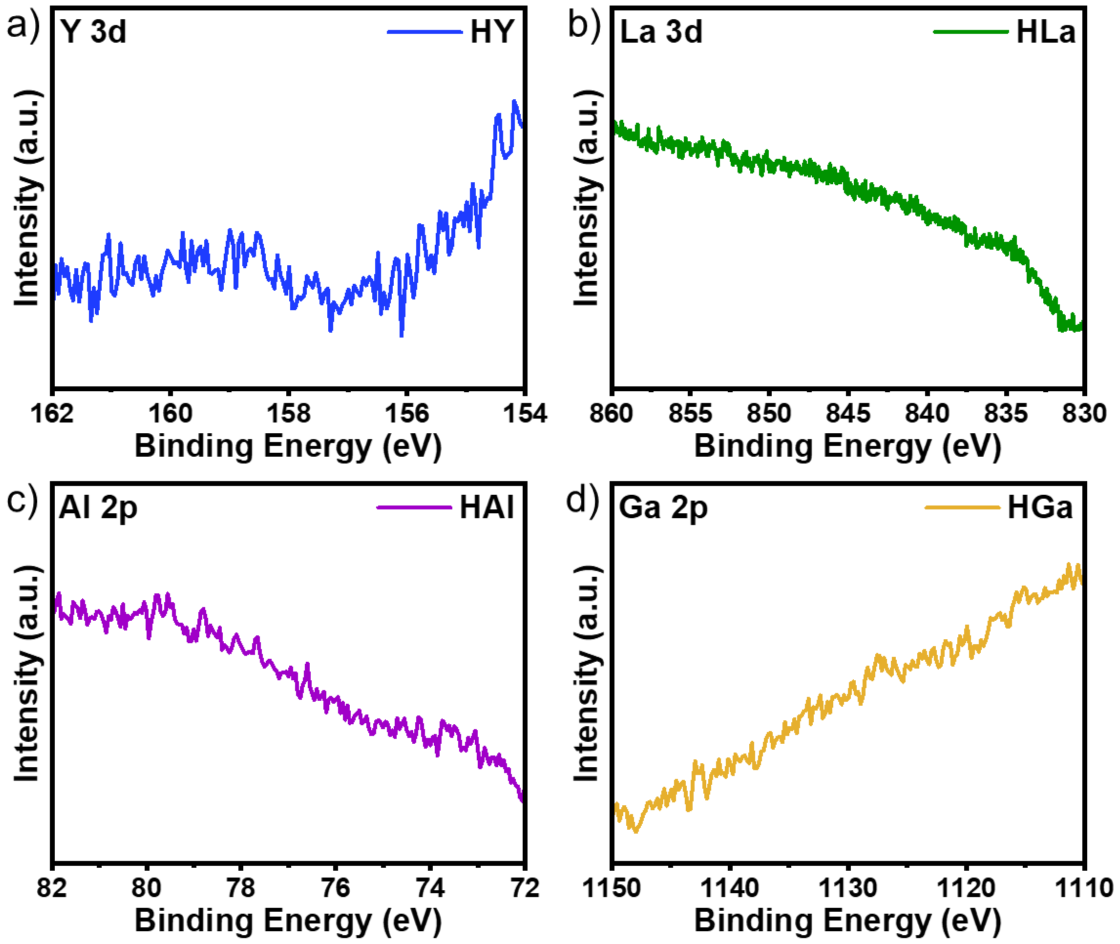

Figure S20. Deconvoluted high-resolution XPS spectra of a) Y 3d from HY, b) La 3d from HLa, c) Al 2p from HAl, and d) Ga 2p from HGa.

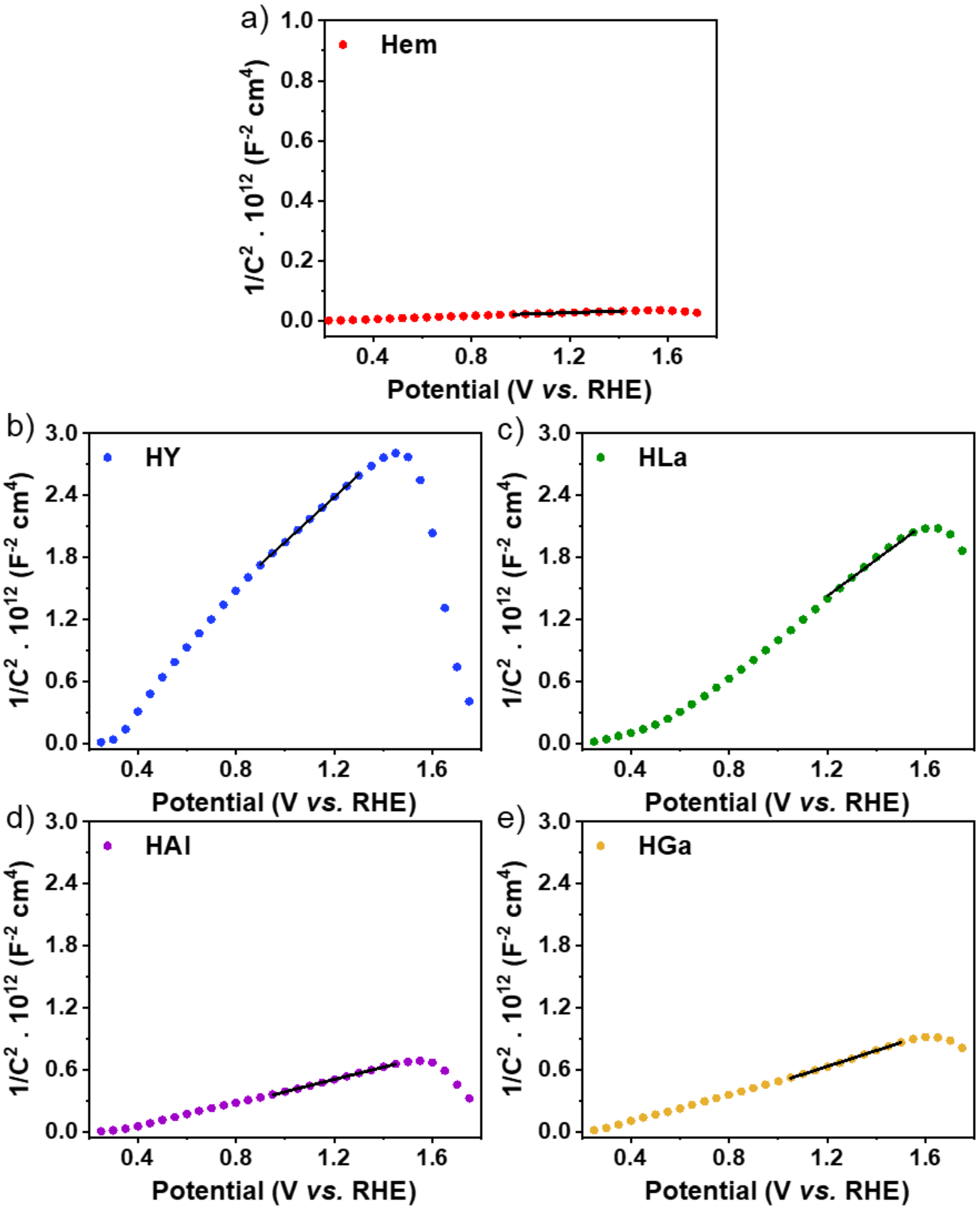

Figure S21. Mott-Schottky analysis of a) pristine hematite (Hem), b) HY, c) HLa, d) HAl, and e) HGa photoanodes. Data was extracted from electrochemical impedance spectroscopy (EIS) at the frequency of 1 kHz.

Table S10. Calculated charge donor density (N_D) for pristine hematite (Hem), HY, HLa, HAl and HGa photoanodes. Data extracted from Mott-Schottky analysis.

Photoanode	N_D (cm^{-3})
**Hem**	10^{20}
**HY**	10^{19}
**HLa**	10^{19}
**HAl**	10^{19}
**HGa**	10^{19}

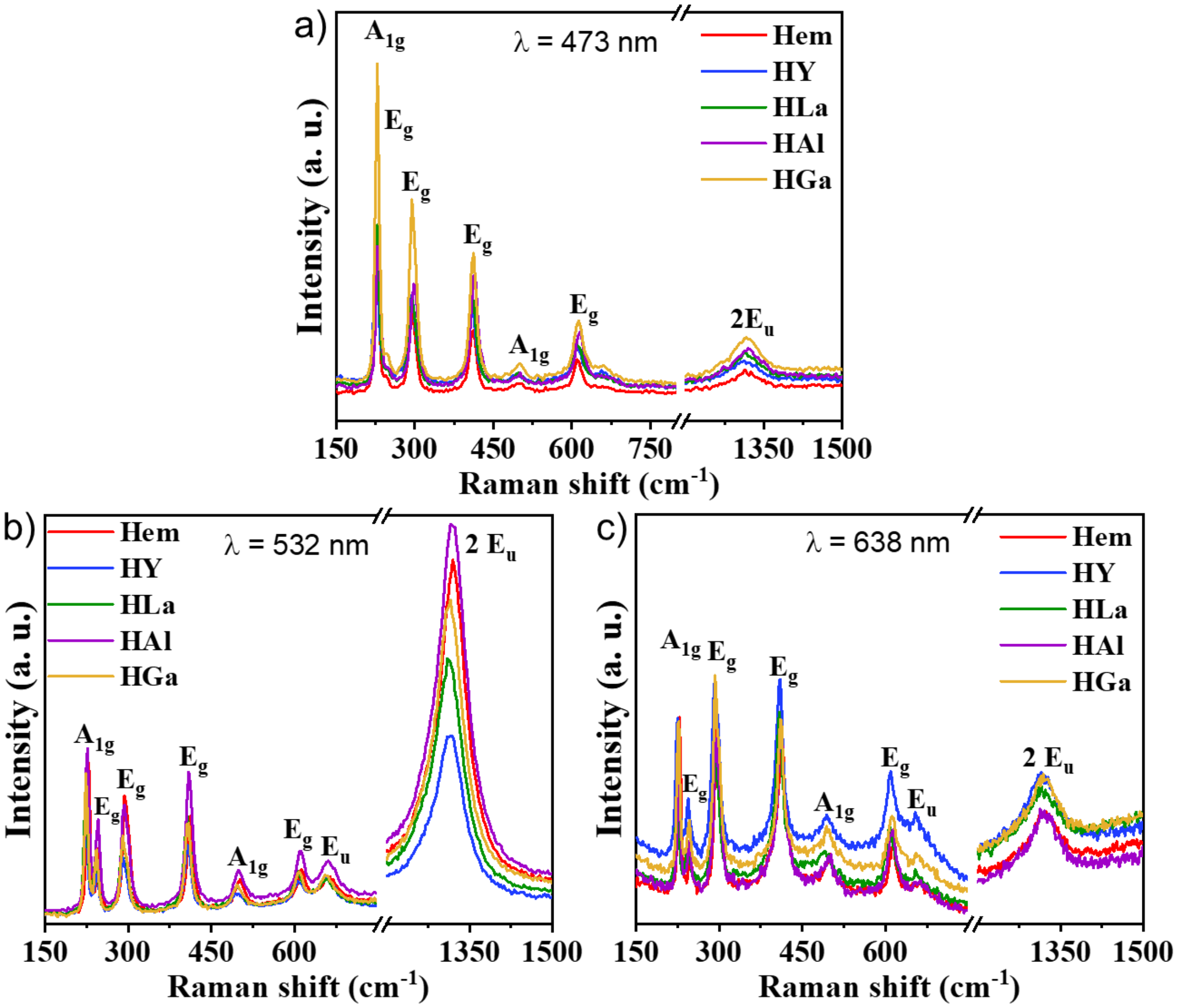

Figure S22. Raman spectra obtained for pristine hematite (Hem), HY, HLa, HAl and HGa photoanodes acquired through the irradiation of lasers with wavelength of a) 473 nm (blue), b) 532 nm (green), and c) 638 nm (red).

Table S11. Calculated full width at half maximum (FWHM) of the E_u peak at 660 cm^{-1} obtained from Raman spectroscopy analysis at λ = 532 nm for pristine hematite (Hem), HY, HLa, HAl and HGa photoanodes.

**Photoanode**	**FWHM E_u peak (660 cm^{-1})**
	**λ = 532 nm**
**Hem**	37
**HY**	34
**HLa**	35
**HAl**	36
**HGa**	37

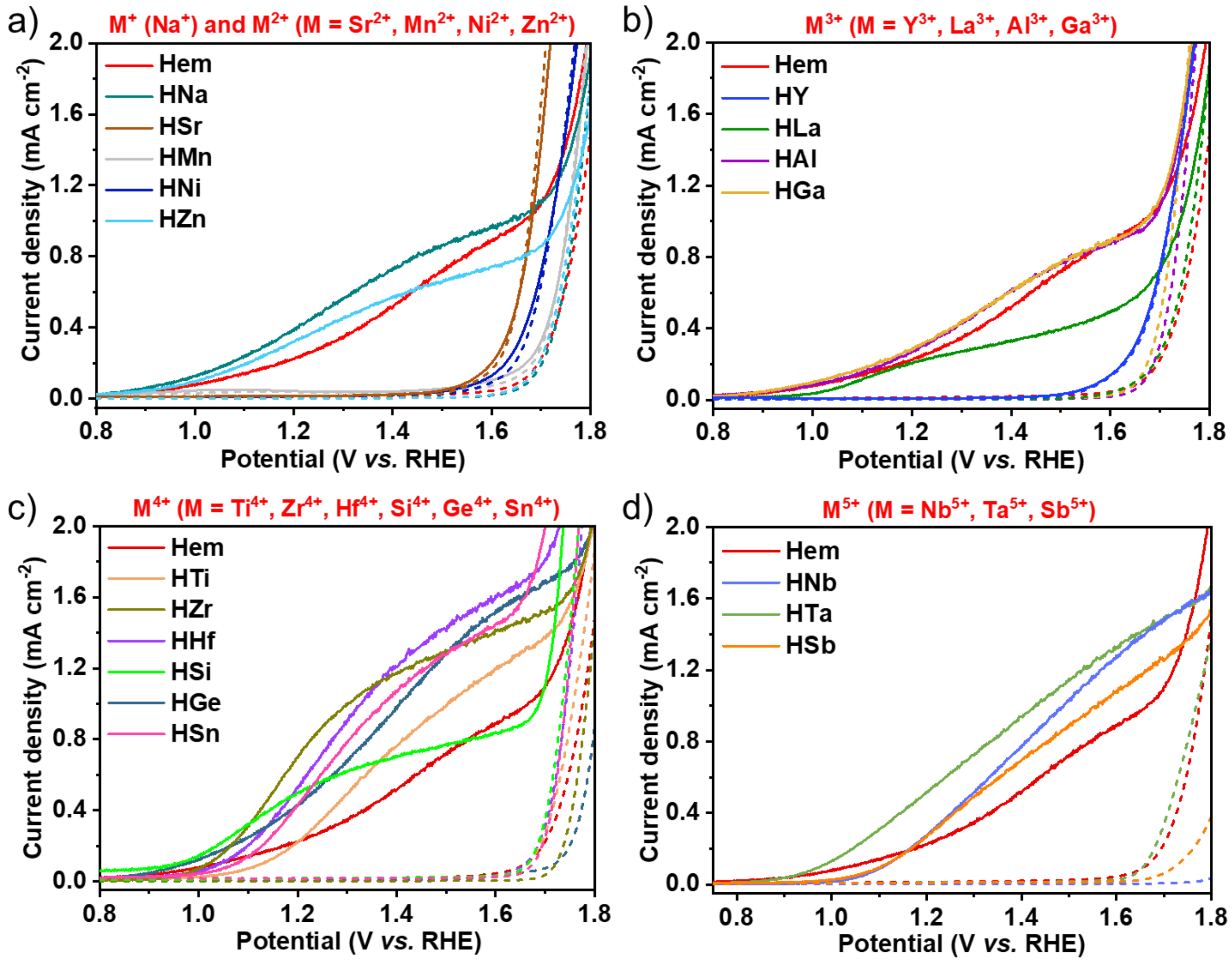

Figure S23. Photocurrent density profiles of modified hematite photoanodes under dark (dashed lines) and simulated sunlight illumination (straight lines) conditions. Modifiers (M^{X+}) were incorporated into the iron precursor solution during Step 2 of the PPS protocol (HM), with the photoelectrochemical behavior screening of a) M^{+} and M^{2+}, b) M^{3+}, c) M^{4+}, and d) M^{5+} modifying chemical elements. All data is presented in comparison to pristine hematite (Hem) and was recorded under the illumination of 100 mW cm^{-2} (1 Sun) using 1M NaOH as the supporting electrolyte.

1.4. Case study for dopant choices targeting high PEC performance on hematite

SUMMARY:

- **Figure S24 –** HAADF-STEM images, photocurrent density profile, 48-hour stability test, and gas evolution over time for the AlHZr photoanode 36
- **Figure S25 –** LSV curves and XPS spectra (Fe 2p, Al 2p, Zr 3d, and O 1s) of the AlHZr photoanode before and after the 48-hour stability test 37
- **Figure S26 –** UPS data of pristine hematite, AlH (0.5%), HZr (3.0%), and AlHZr photoanodes 38
- **Figure S27 –** Absorbance spectra and Tauc plots for pristine hematite, AlH (0.5%), HZr (3.0%), and AlHZr photoanodes 39
- **Figure S28 –** UPS data of pristine and Step 1-modified hematite 40
- **Figure S29 –** Band diagrams of pristine and Step 1-modified hematite 41
- **Figure S30 –** UPS data of pristine and Step 2-modified hematite 42
- **Figure S31 –** Band diagrams of pristine and Step 2-modified hematite 43
- **Table S12 –** Comparative table of the performance of chemically modified hematite photoanodes synthesized by different methods 44
- **Figure S32 –** Bar chart comparing the performance of chemically modified hematite photoanodes synthesized by different methods 45

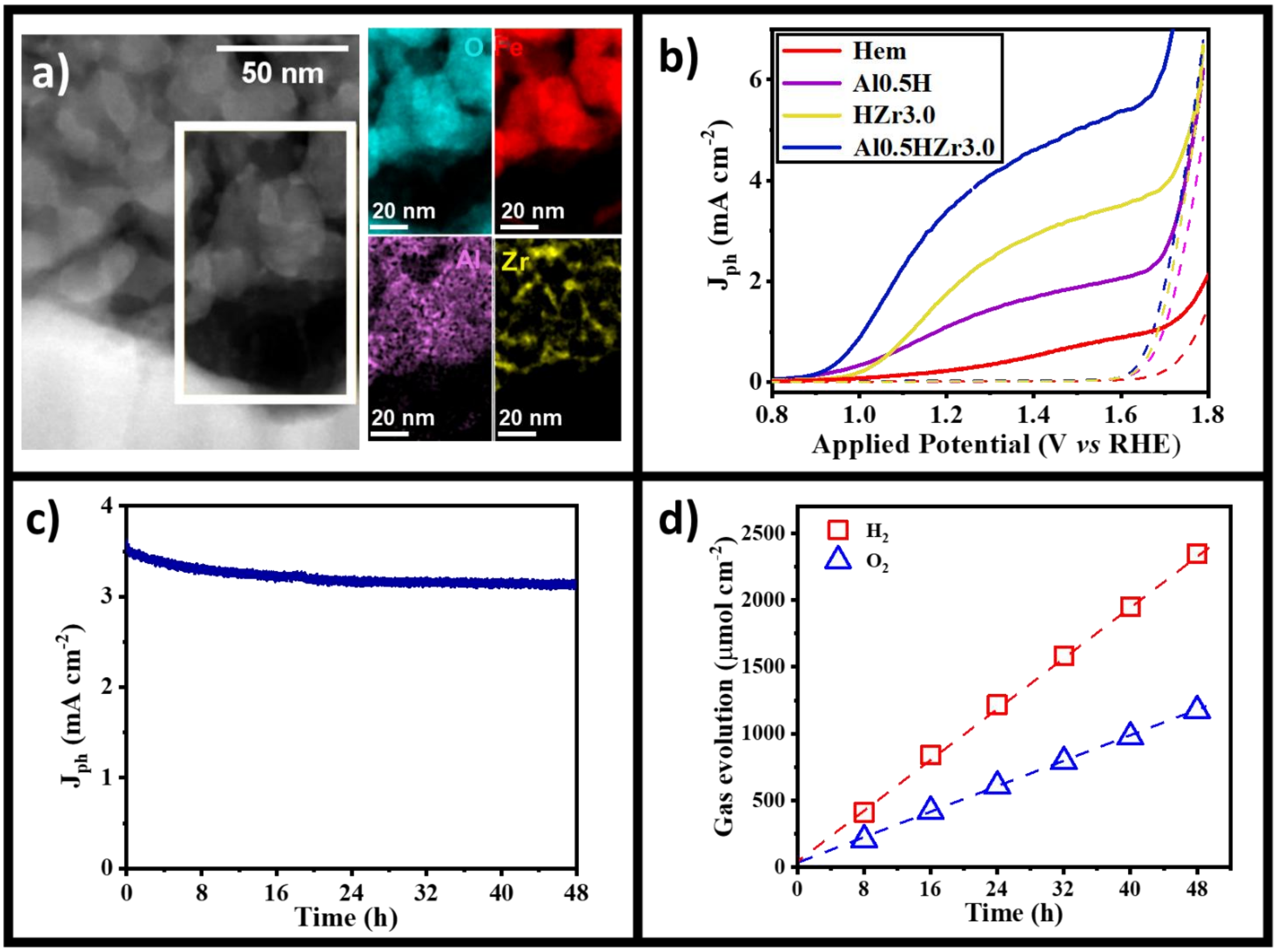

Figure S24. a) High-angle annular dark-field scanning transmission electron microscopy (HAADF-STEM) spectra of hematite photoanode modified with Al^{3+}/Zr^{4+}. Energy dispersive X-ray spectroscopy (EDS) was performed in the sample region marked by the white square, showing the distribution of Fe^{3+}, Al^{3+}, Zr^{4+}, and O^{2-}. b) Linear sweep voltammetry measurements of pristine hematite and photoanodes modified with Al^{3+}/Zr^{4+} under 100 mW cm^{-2} illumination. c) Stability test of dual modified AlHZr photoanode under light (100 mW cm^{-2}) for 48 hours. d) H_2 and O_2 evolution as a function of time recorded for AlHZr photoanode.

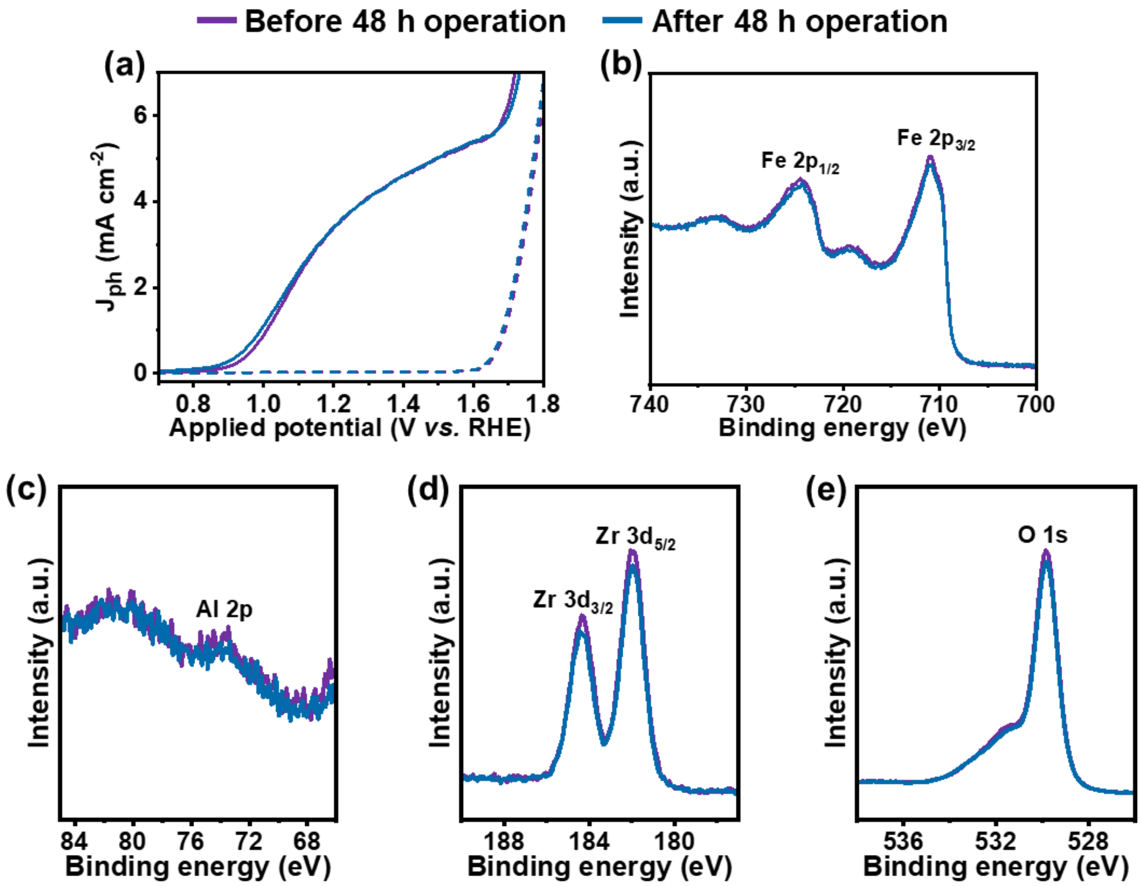

Figure S25. a) Linear sweep voltammetry (LSV) curve of AlHZr photoanode before and after the 48-hour stability test. High-resolution X-ray photoelectron spectroscopy (XPS) spectra of b) Fe 2p, c) Al 2p, d) Zr 3d, and e) O 1s of AlHZr sample before and after the long-term stability test.

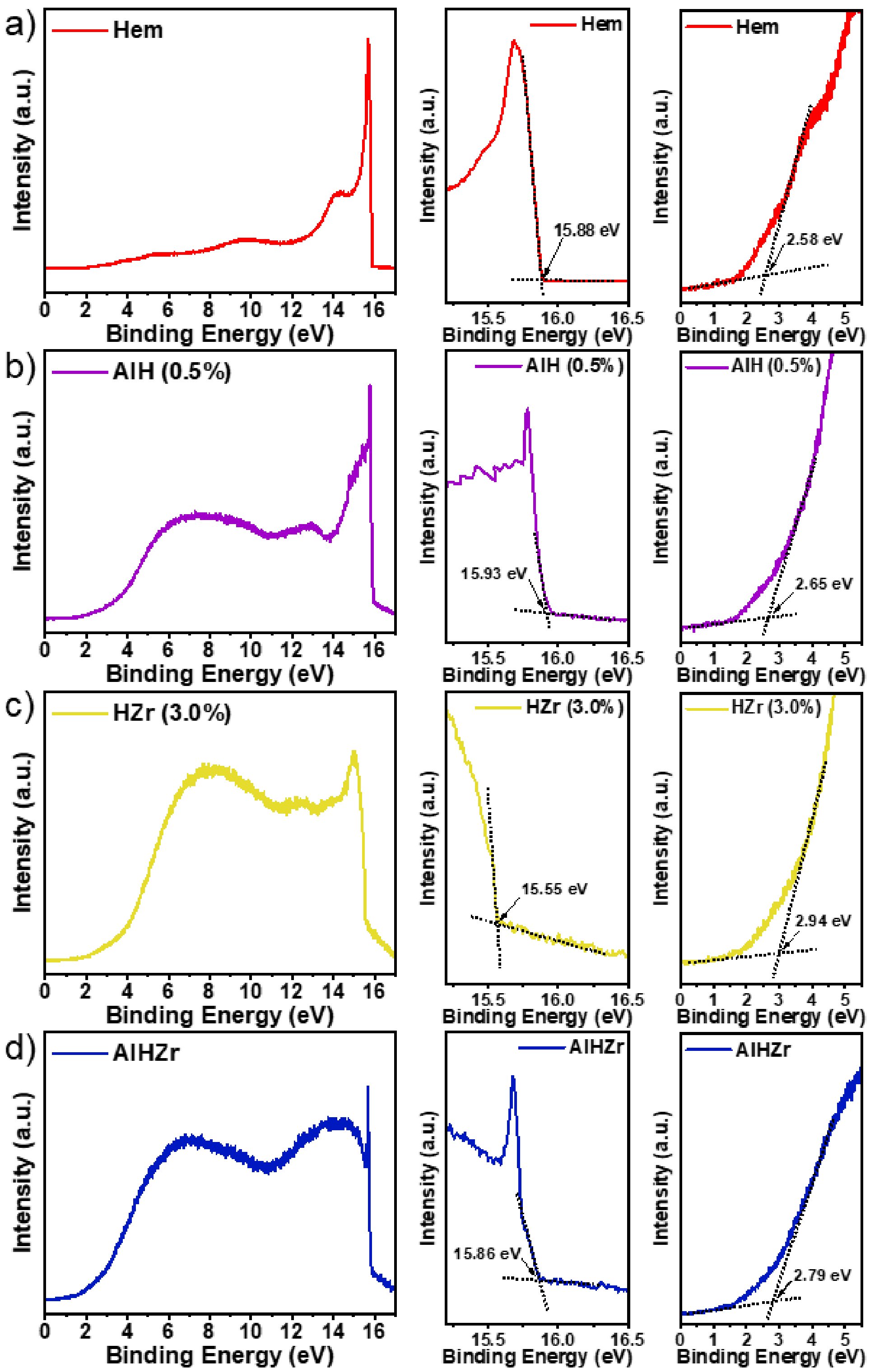

Figure S26. He I UPS plots obtained for a) pristine hematite (Hem), b) AlH (0.5%), c) HZr (3.0%), and d) AlHZr photoanodes. The first column shows the acquired spectra. Second and third columns exhibit, respectively, the determination of the secondary electron cutoff (SECO) and the expansion of the region near the Fermi level to determine the valence band maximum.

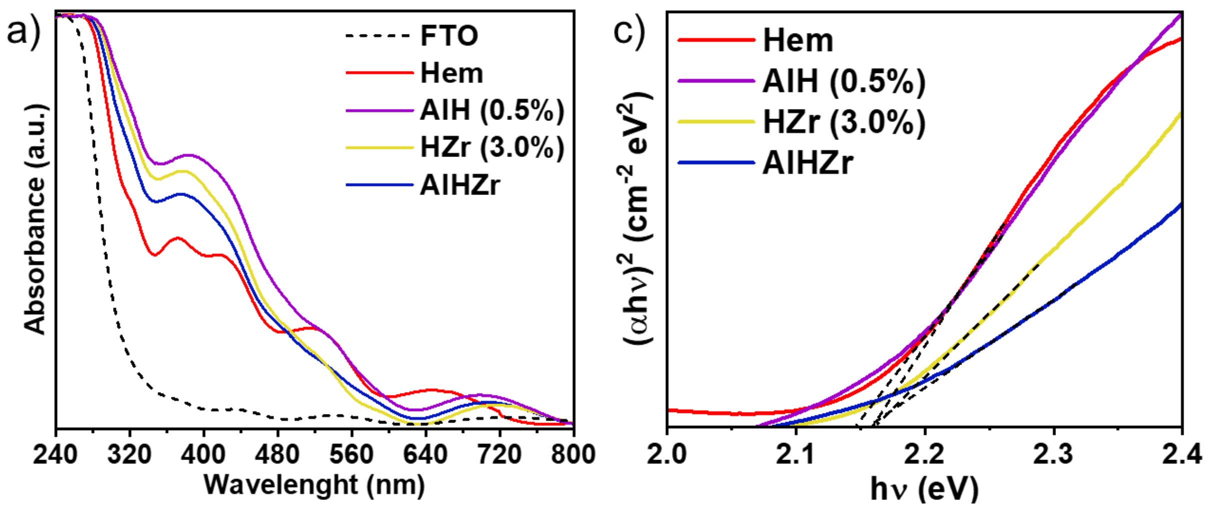

Figure S27. a) Absorbance spectra of pristine hematite (Hem), AlH (0.5%), HZr (3.0%), and AlHZr photoanodes. b) Tauc plots for direct band-gap determination of pristine hematite (Hem), AlH (0.5%), HZr (3.0%), and AlHZr photoanodes. Dashed lines represent the linear fit of the curve's linear regions.

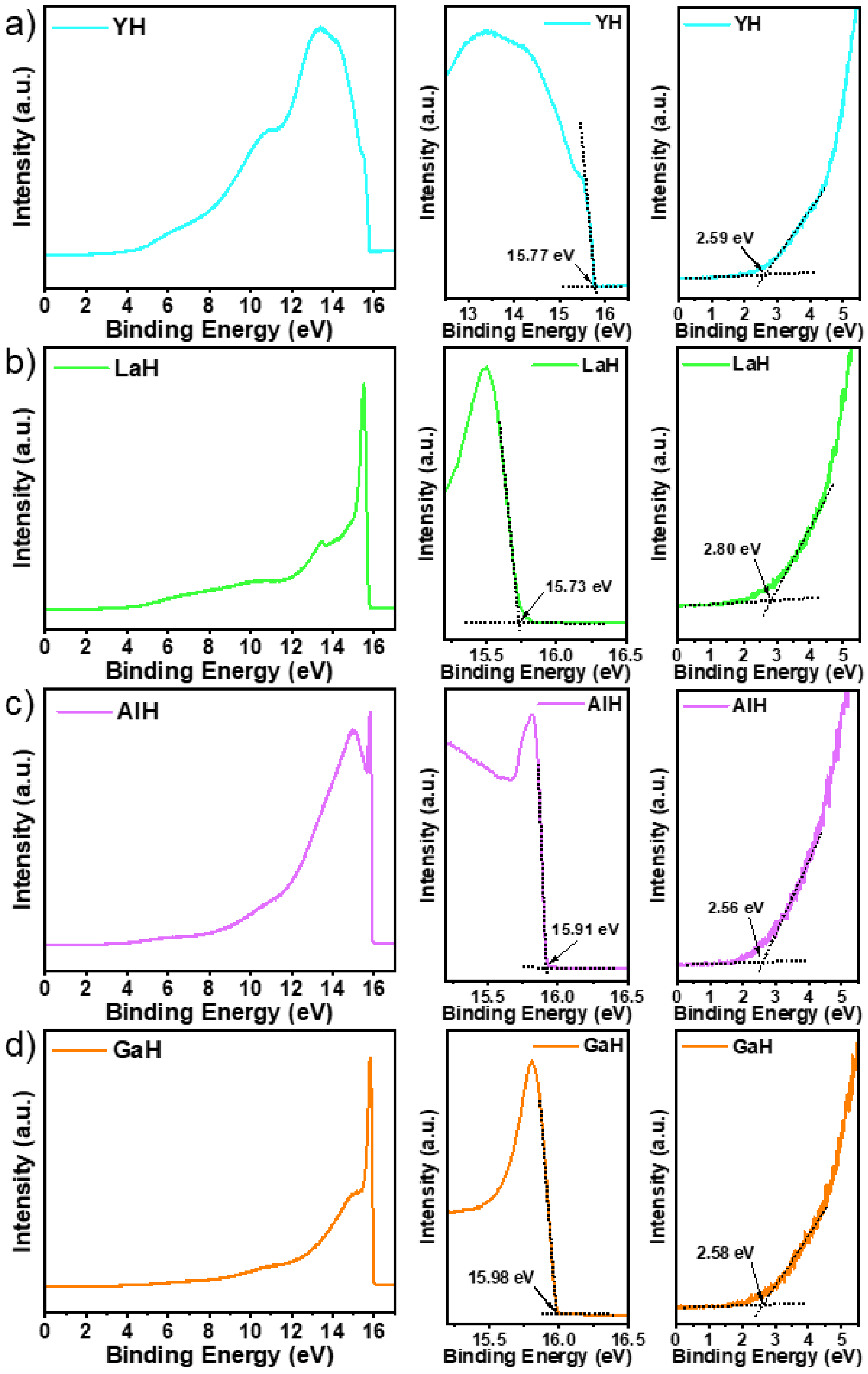

Figure S28. He I UPS plots obtained for a) YH, b) LaH, c) AlH, and d) GaH photoanodes. The first column shows the acquired spectra. Second and third columns exhibit, respectively, the determination of the secondary electron cutoff (SECO) and the expansion of the region near the Fermi level to determine the valence band maximum.

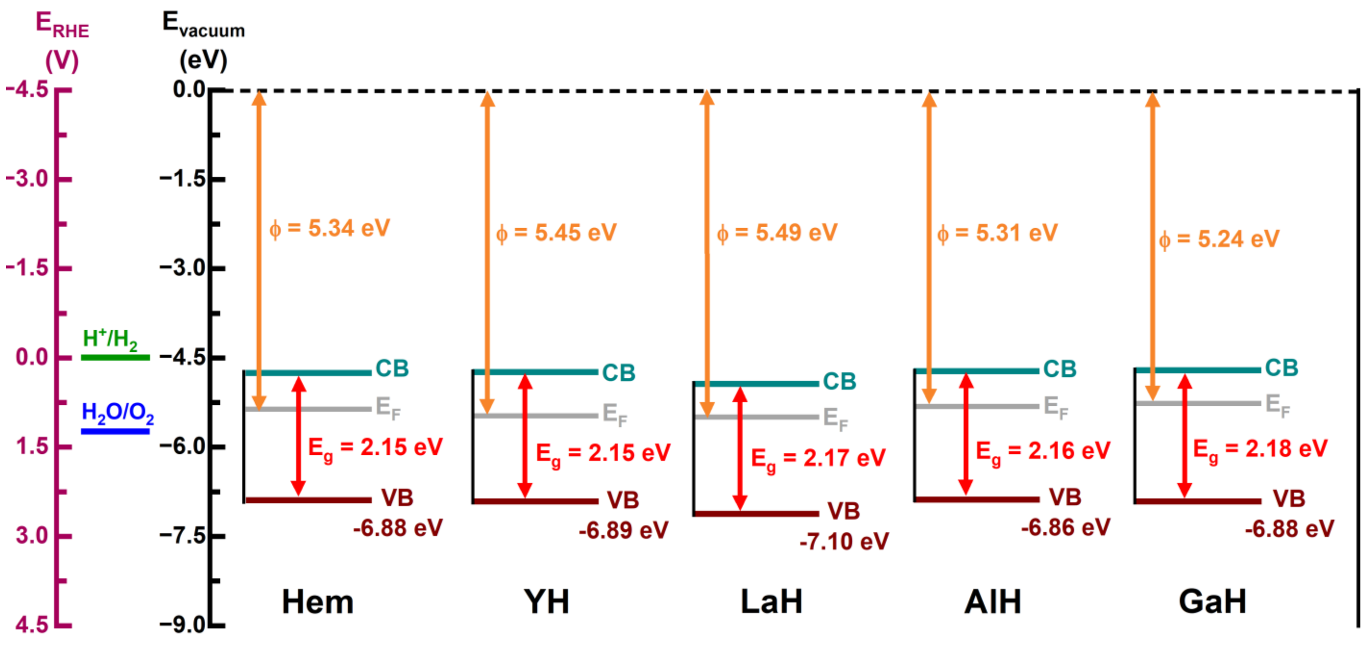

Figure S29. Band diagrams constructed from UPS data (Figure S28) and Tauc plots (Figure S8c) for pristine hematite (Hem), YH, LaH, AlH and GaH photoanodes.

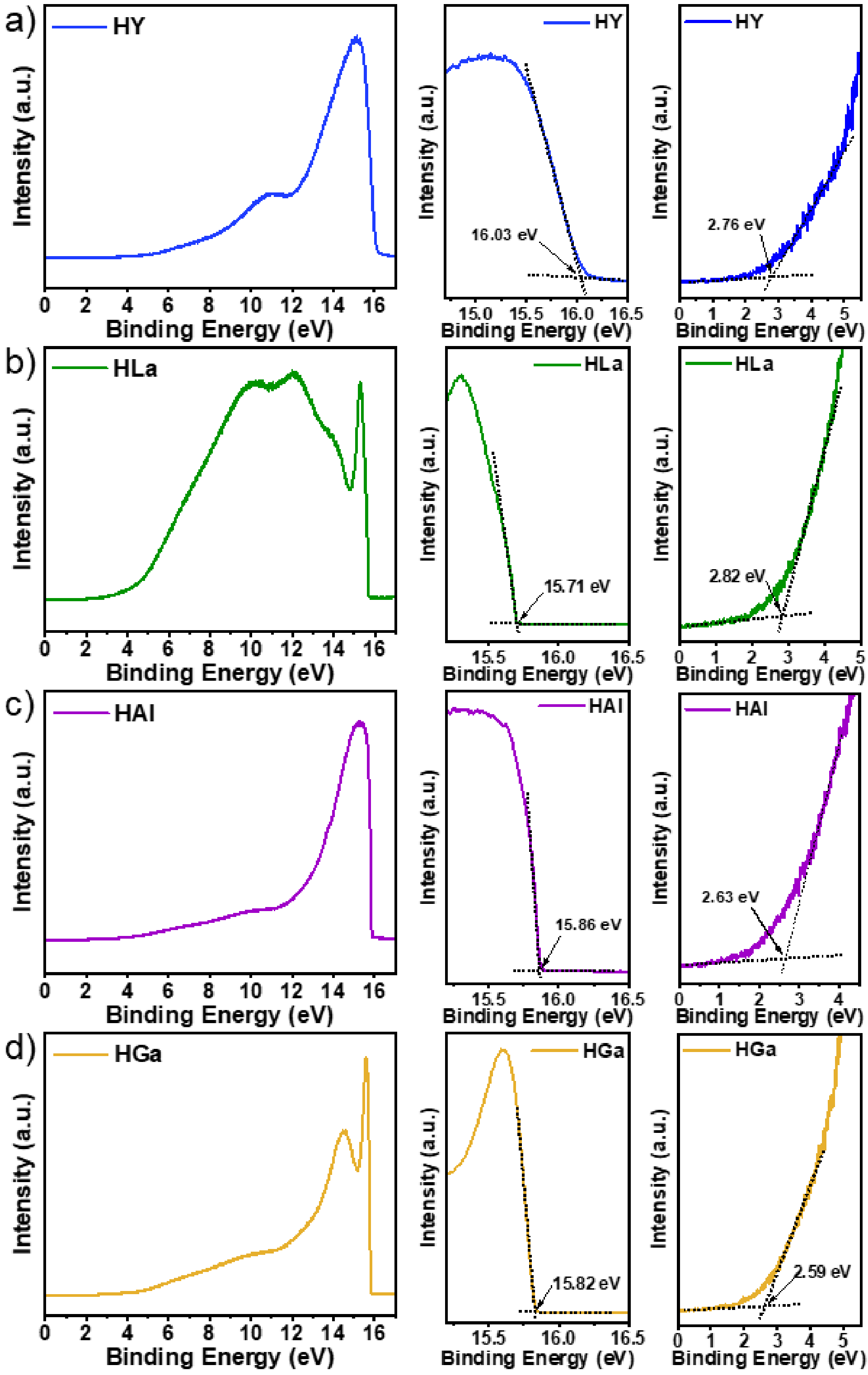

Figure S30. He I UPS plots obtained for a) HY, b) HLa, c) HAl, and d) HGa photoanodes. The first column shows the acquired spectra. Second and third columns exhibit, respectively, the determination of the secondary electron cutoff (SECO) and the expansion of the region near the Fermi level to determine the valence band maximum.

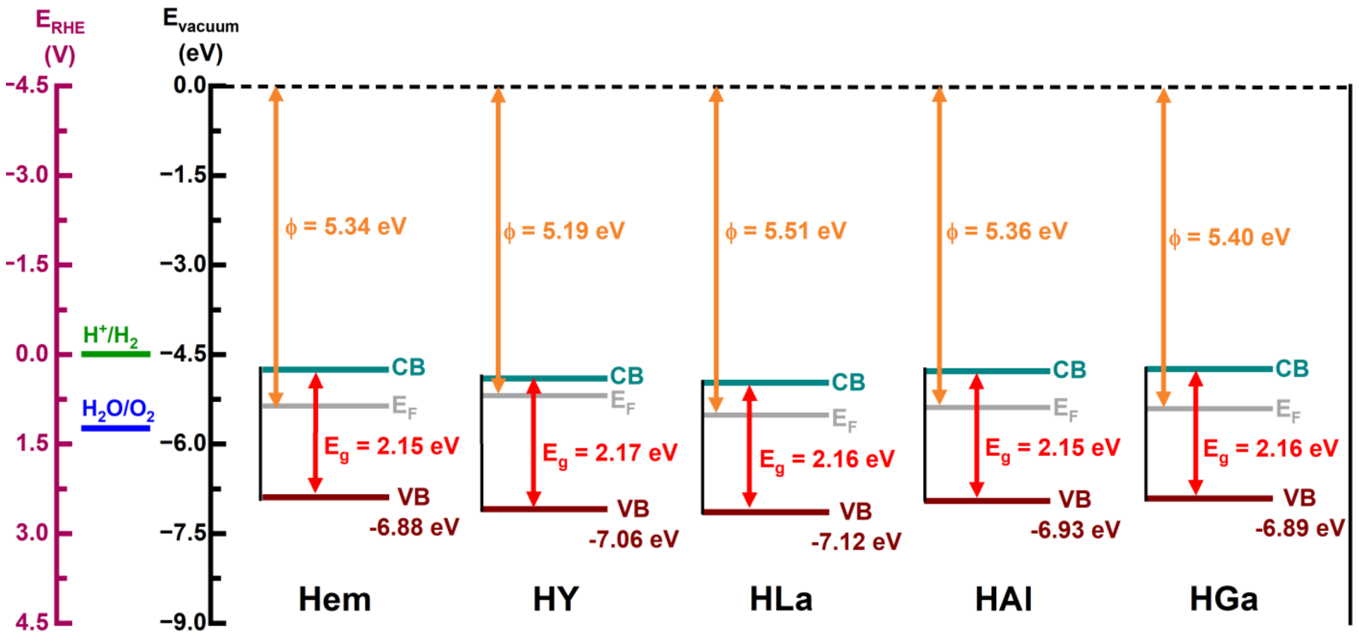

Figure S31. Band diagrams constructed from UPS data (Figure S30) and Tauc plots (Figure S18c) for pristine hematite (Hem), HY, HLa, HAl, and HGa photoanodes.

Table S12. Comparative photoelectrochemical performance of hematite photoelectrodes modified with the same chemical elements employed in this work and produced through different synthesis methods.

Photoanode	Employed modifiers	Methodology	J_{ph} @ 1.23 V_{RHE} (mA cm^{-2})	Reference
$NiFeO_x$/Al:Ti-Fe_2O_3	Al^{3+} and Ti^{4+} (co-doping), $NiFeO_x$ co-catalyst	Hydrothermal, spin-coating treatment	4.00	[10]
**Al0.5HZr3.0**	Al^{3+} and Zr^{4+} (co-doping)	**PPS protocol**	3.65	**This work**
CoPi/2:Al-Zr/HT	Al^{3+} and Zr^{4+} (co-doping), CoPi co-catalyst	Hydrothermal, magnetron sputtering	1.98	[11]
Al/Zr-HT	Al^{3+} and Zr^{4+} (co-doping)	Hydrothermal, microwave irradiation	1.88	[12]
**Ga0.5HHf3.0**	Ga^{3+} and Hf^{4+} (co-doping)	**PPS protocol**	1.81	[13]
Zr-Fe_2O_3 (I) NC - Al12%+Co-Pi	Al^{3+} and Zr^{4+} (co-doping), CoPi co-catalyst	Hydrothermal, dip-coating treatment	1.80	[14]
Pt/Al-HT	Pt^{4+} and Al^{3+} (co-doping)	Hydrothermal, microwave heat treatment	1.55	[15]
CoFe-MOF/Zr-Y:Fe_2O_3	Y^{3+} and Zr^{4+} (co-doping), CoFe-MOF co-catalyst	Hydrothermal	1.51	[16]
Fe_2O_3 - 6% Zr + 6% Al + 5mM Co	Al^{3+} and Zr^{4+} (co-doping), CoO_x co-catalyst	Hydrothermal, dipping method	1.51	[17]
Al(5mM)/Ti(4 %):Fe_2O_3	Al^{3+} and Ti^{4+} (co-doping)	Hydrothermal, microwave-assisted surface attachment	1.32	[18]
Ti-Fe_2O_3-Al	Ti^{4+} (doping), Al^{3+} (surface treatment)	Hydrothermal, chemical bath deposition	1.30	[19]
Fe_2O_3-0.46%Al	Al^{3+} (doping)	Electrodeposition	~ 1.00	[20]
YCuFe-1	Y^{3+} and Cu^{2+} (co-doping)	Hydrothermal	0.44	[21]
(Ga, Fe)$_2$O$_3$	Ga^{3+} (foward gradient doping)	Precursor spin-coating, thermal treatment	0.44	[22]

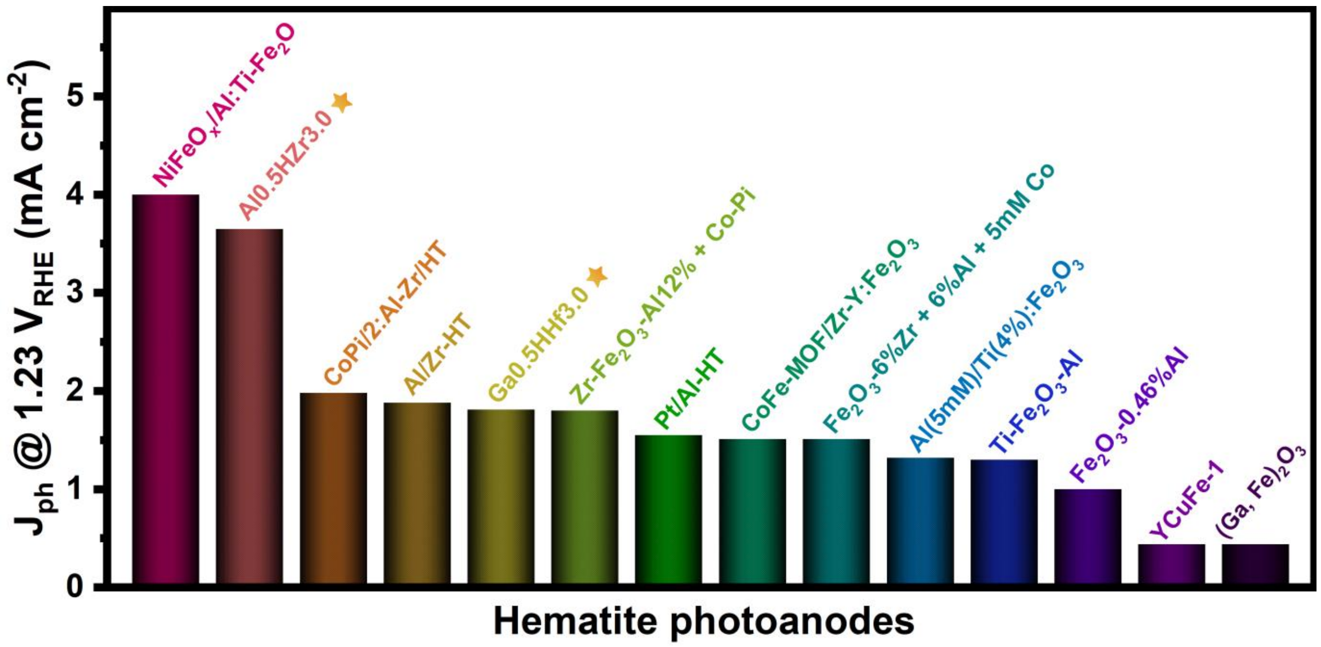

Figure S32. Bar chart comparing the photocurrent densities at 1.23 V_{RHE} of hematite photoanodes produced through the PPS methodology (marked with a yellow star) to those modified with similar elements using various synthesis methods reported in the literature.

2. EXPERIMENTAL SECTION

Structural, optical, and morphological characterizations

X-ray diffraction (XRD) patterns of pristine and chemically modified hematite photoanodes were determined through grazing incidence X-ray diffraction (GIXRD) in a Bruker D8 Advance ECO-AXS diffractometer. Cu Kα (λ = 1.54 Å) was employed as the incident radiation. The analysis was conducted in the 10° < 2θ < 90° range, with a step size of 0.02° and step time of 14 seconds. α-Fe_2O_3 and SnO_2 peaks were assigned according to respective JCPDS cards. Cell parameters of all samples were calculated through the rhombohedral system formula and considering experimentally measured peak positions. The Lotgering factor (F) of the (1 1 0) plane was calculated through Equation S1,[23] in which $P = \sum I(h\ 0\ 0)/I(h\ k\ l)$, P_0 is the P equivalent for the reference hematite JCPDS card (n° 33-0664), and I is the intensity of the peaks obtained in the analysis. The crystallite size (T_c) in the (1 1 0), (1 0 4) and (3 0 0) planes were calculated through Equation S2,[24] where K is the shape factor (K = 0.9), λ is the incident radiation wavelength, w is the full width at half maximum (FWHM) of the peak, and θ is Bragg's reflection angle.

$$F = \frac{P - P_0}{1 - P_0} \quad \text{(Equation S1)}$$

$$T_c = \frac{K\lambda}{w.\cos\theta} \quad \text{(Equation S2)}$$

The morphology of the photoelectrodes was examined by scanning electron microscopy (SEM) from in situ milling using a gallium focused ion beam (FIB) system in a dual beam scanning electron microscopy (Scios 2 DualBeam, Thermo Fisher Scientific). Thickness was estimated from the images using the ImageJ software. Topographical analysis was conducted through atomic force microscopy (AFM). AFM measurements were performed in a NX-10 Park Systems microscope, with a NanoSensorsTM Pt/Ir-coated silicon probe, PPP-EFM model. A resonance frequency (nominal) of 75 kHz was applied, as well as a force constant (nominal) of 2.8 N m^{-1}. Scanning image areas of 1 µm × 1 µm with a resolution of 512 × 512 pixels were recorded.

X-ray photoelectron spectroscopy (XPS) measurements were conducted using an Omicron-Scienta spectrometer equipped with a monochromatic Al Kα X-ray source (hν = 1486.6 eV) operated at 11 mA and

15 kV. The spectra were recorded in ultra-high vacuum conditions (pressure < 10^{-9} mbar) to minimize contamination and ensure accurate surface analysis. A hemispherical analyzer was used, with a pass energy of 30 eV and a step size of 0.05 eV, providing an overall energy resolution of 0.7 eV, as determined from the full width at half maximum (FWHM) of the Ag $3d_{5/2}$ peak. Spectral deconvolution was performed using CasaXPS software (version 2.3.27), applying a Shirley-type background subtraction. The binding energy scale was calibrated using the C 1s peak at 284.8 eV as an internal reference to correct for possible charging effects. Peak fitting was conducted with a combination of Gaussian–Lorentzian functions to accurately represent the core-level signals. To mitigate charging effects, a low-energy electron flood gun was employed for charge compensation.

Raman analysis was conducted in a XploRA Plus Confocal Raman instrument equipped with a Syncerity OE detector. Experiments were conducted in the 100-1800 cm^{-1} range, with blue (λ = 473 nm), green (λ = 532 nm) and red (λ = 638 nm) lasers being used as excitation sources. An acquisition time of 10 seconds was employed, and the spectra was recorded from 10 accumulations. The FWHM of the E_u peak located at 660 cm^{-1} was determined by deconvoluting the obtained spectra of all samples in Origin 2024 software. The peaks were fitted after appropriate background subtraction.

Optical measurements were performed in a Shimadzu UV-VIS-NIR spectrophotometer (UV-3600 Plus) equipped with an integrating sphere, recording the spectra from 220 to 800 nm. The optical band gap values for the photoanodes were estimated by the Tauc relation, as shown in Equation S3,[25] where α is the absorption coefficient, *h* is the Planck constant, ν is the frequency, *n* is ½ for allowed indirect electronic transitions and 2 for allowed direct transitions, *A* is a constant, and E_g is the estimated optical band gap energy.

$$(\alpha h\nu)^n = A\left(h\nu - E_g\right) \qquad \text{(Equation S3)}$$

Ultraviolet photoelectron spectroscopy (UPS) experiments were performed in a Specs XPS/UPS system with a Phoibos 150 analyzer and a CMOS 2D detector under ultra-high vacuum (>10^{-8} Pa). The photoanodes were irradiated with a He I line ($h\nu$ = 21.22 eV) excitation source from an ultra-high vacuum (UHV) gas discharge lamp. Spectra were recorded from -1.0 to 19.0 eV with a pass energy of 1.0 eV. An acceleration potential of -4.0 V was applied to pristine hematite (Hem), HY, HLa, HAl, HGa, AlH (0.5%),

HZr (3.0%), and AlHZr samples, while a potential of -4.5 V was applied to YH. LaH, AlH, and GaH photoanodes were subjected to a -5.0 V potential application. The work function (ϕ) of pristine and chemically modified photoelectrodes was determined through Equation S4,[26] in which $h\nu$ is the incident He I line energy and SECO is the secondary electron cutoff energy. Equation S5 was used to calculate the valence band (VB) position, where VB_{energy} is the valence band maximum energy determined at the expansion of the region near the Fermi level and ϕ_{Ag} is the standard silver work function. The conduction band (CB) position was determined by the sum of VB position and E_g calculated through Tauc plots.

$$\phi = h\nu + SECO \quad \text{(Equation S4)}$$

$$VB = -(VB_{energy}) - (\phi_{Ag}) \quad \text{(Equation S5)}$$

Differential scanning calorimetry (DSC) thermograms were obtained with the aid of a NETZSCH DSC 214 Polyma instrument. Polymeric samples were analyzed in the temperature range of -80°C to 60°C, with a heating step of 10.0 K/min.

Scanning transmission electron microscopy (STEM) analysis were performed using a JEOL JEM 2100F microscope equipped with a Gatan Tridiem 863 spectrometer for electron energy loss spectroscopy (EELS) and an Oxford SSD detector for energy dispersive spectroscopy (EDS).

Photoelectrochemical and electrochemical characterizations

Hematite-based photoanodes were assembled in a three-electrode electrochemical cell containing a platinum counter electrode and a commercial (Metrohm Autolab) Ag/AgCl(sat) reference electrode. The photoanodes were employed as the working electrode (area of 0.28 cm^2) in a 1.0 M NaOH (Sigma-Aldrich, 98%) electrolyte solution (pH = 13.6). Experiments were conducted using a potentiostat/galvanostat (Autolab PGSTAT 302N). Linear sweep voltammetry (LSV) measurements were performed under sunlight illumination (100 mW cm^{-2}) simulated by a 450 W Xe lamp (Osram, ozone free) equipped with an AM 1.5 Global filter. A potential range of 0.8-1.8 V_{RHE} was applied, with a scan rate of 50 mV s^{-1}. The power (1 Sun) was adjusted and calibrated to the position of the photoanodes with the aid of an optical power meter (Newport 843-R-USB).

In this work, the photocurrent (J_{ph}) measured at 1.23 V vs. reversible hydrogen electrode (RHE) was considered to be described by Equation S6, where J_{ph} is defined as the product of the maximum current values permitted by the absorption properties (J_{abs}) of the photoanodes and the efficiencies of charge separation ($\eta_{sep.}$) at the bulk and charge injection ate the surface, described as the catalytic efficiency ($\eta_{cat.}$).[27] The maximum current generated due to absorption properties of the nanoporous structures (J_{abs}) was calculated from the absorbance measurements as presented in Equation S7, where q is the elementary electron charge, ø is the photon flux in the AM 1.5 G filter, and λ is in the visible range for the absorption spectra.[28] The product of charge separation and catalytic efficiencies was denoted as the overall efficiency of the materials ($\eta_{overall}$), being calculated as the total yield of current generated in relation to the maximum current permitted by the absorption properties (J_{abs}) of the photoanodes (Equation S8).

$$J_{ph} = J_{abs} \cdot \eta_{sep} \cdot \eta_{cat} \quad \text{(Equation S6)}$$

$$J_{abs} = -q\,ø\left(1 - e^{(-\int Abs\, d\lambda)}\right) \quad \text{(Equation S7)}$$

$$\eta_{overall} = \frac{J_{ph}}{J_{abs}} \quad \text{(Equation S8)}$$

The electrochemically active surface area (ECSA) of hematite-based photoanodes was estimated through cyclic voltammetry (CV) under dark conditions. CV scans were performed in the non-Faradaic region of -0.3 to -0.1 V *vs.* Ag/AgCl, employing scan rates of 50 mV, 100 mV, 150 mV, 200 mV and 250 mV. The double-layer capacitance (C_{DL}) of the interface was determined considering Equation S9, in which j_a and j_c are the anodic and cathodic current densities, respectively, and v is the scan rate. The average j obtained from CV scans was plotted against v for all samples, with the slope of the curve corresponding to C_{DL}. The ECSA was then calculated through Equation S10,[29] where C_s is the specific capacitance for 1 M NaOH (C_s = 0.040 mF cm^{-2}).[30]

$$C_{DL} = \frac{j_a - j_c}{2} \cdot \frac{1}{\nu} \quad \text{(Equation S9)}$$

$$ECSA = \frac{C_{DL}}{C_s} \quad \text{(Equation S10)}$$

The charge carrier dynamics of the photoelectrodes were investigated by intensity modulated photocurrent spectroscopy (IMPS) using an Autolab PGSTAT 302N potentiostat coupled to a FRA32M module and an Autolab LED driver. Samples were assembled in the three-electrode electrochemical cell

and irradiated with blue (λ = 470 nm) high intensity LED. The base light intensity incident at the photoanodes was calibrated to be 40 mW cm^{-2}. A sinusoidal light modulation (20 kHz to 0.1 Hz frequency range) was employed, with a sinusoidal modulation amplitude of 15% of base light intensity. The charge separation efficiency *versus* light harvesting efficiency (CSE x LHE), external quantum efficiency (EQE) and charge transfer efficiency (η_{transf}) parameters were calculated according to IMPS general theory.[31]

Mott-Schottky measurements were extracted from electrochemical impedance spectroscopy (EIS) analysis performed under dark conditions in the frequency range of 10^5 to 10^2 Hz. Data was extracted at 1 kHz from 0.25 to 1.75 V_{RHE}. The charge donor density (N_D) of the photoanodes were estimated through Equation S11,[32] where C_{SC} is the space charge layer capacitance, ε is the dielectric constant of the material (for hematite, ε = 80), ε_0 is the vacuum permittivity (ε_0 = 8.8542 x 10^{-12} F m^{-1}), A is the photoanode area, e is the electron charge (e = 1.60 x 10^{-19} C), V is the applied potential, V_{FB} is the flat band potential, k is the Boltzmann constant (k = 1.38 x 10^{-23} J K^{-1}) and T is the temperature.

$$\frac{1}{C_{SC}^2} = \frac{2}{\varepsilon.\varepsilon_0.A^2.e.N_D}\left(V - V_{FB} - \frac{kT}{e}\right) \quad \text{(Equation S11)}$$

The generated photocurrent (J_{photo}) and photopotential (V_{photo}) of the dual modified AlHZr photoanode were determined as described in reference [33]. The photocurrent (J_{photo}) generated by the photoelectrode within the potential range of 0.9 to 1.8 V_{RHE} was obtained by extrapolating the current density curve under simulated sunlight illumination (J_{light}) in the region where no electrolysis contribution is observed. The intrinsic photovoltaic power (P) of the photoelectrode was estimated through Equation S12. The intrinsic solar to chemical conversion efficiency (ISTC) of the sample was calculated through Equation S13, where U_{dark} is the potential that needs to be applied to the photoelectrode under dark conditions to match the photocurrent (J_{photo}) generated under the simulated sunlight illumination of 100 mW cm^{-2}.[33]

$$P(\%) = J_{photo} \times V_{photo} \quad \text{(Equation S12)}$$

$$ISTC(\%) = \frac{1.23\ (V_{RHE})}{U_{dark}\ (V_{RHE})} \times \left[\frac{J_{photo} \times V_{photo}}{100\ (mW\ cm^{-2})}\right] \times 100 \quad \text{(Equation S13)}$$

The long-term stability test of the dual modified AlHZr photoanode was evaluated through chronoamperometry under 100 mW cm^{-1} simulated sunlight illumination for 48 hours. A potential of 1.23

V_{RHE} was applied to the sample, and signals were recorded with an interval of 1 second. During the stability test, gas chromatography was employed to quantify the gaseous products (hydrogen and oxygen). A 50 µL aliquot of the products was extracted from the photoelectrochemical cell every 8 hours and analyzed in a GC System 7890B Agilent Technologies instrument equipped with a thermal conductivity detector (TCD). The total quantity of gases in the cell was quantified considering the total cell volume and previously constructed O_2 and H_2 calibration curves.

All potentials used to report electrochemical data were converted to the reversible hydrogen electrode (RHE) using the Nernst Equation (Equation S14):

$$E_{RHE} = E_{Ag/AgCl} + E^{0}_{Ag/AgCl} + 0.059\ pH \qquad \text{(Equation S14)}$$

Density functional theory (DFT) calculations

First-principles calculations based on Density Functional Theory were performed using the Vienna Ab initio Simulation Package (VASP). For the exchange-correlation energy, the generalized gradient approximation (GGA) as parameterized by Perdew, Burke, and Ernzerhof (PBE) was employed.[34] A plane-wave basis set with an energy cutoff of 400 eV was used to expand the Kohn-Sham orbitals. Electron-ion interactions were described using the Projector Augmented Wave (PAW) method.[35] Geometrical relaxations were carried out until the forces on all atoms were less than 0.025 eV/Å. A Gamma-centered 1 x 3 x 3 Monkhorst-Pack k-point mesh was utilized for Brillouin zone (BZ) integration in the grain boundary (GB) simulations.[36] To account for the strong on-site Coulomb interactions characteristic of Fe *d* orbitals, a Hubbard U correction (DFT+U) with U_{eff} = 4 eV was applied to the Fe sites;[37] this value is established to accurately reproduce the magnetic ordering and electronic bandgap of hematite.[38]

To analyze electron transport across the GB potential barrier, a quantum tunneling model based on the free electron approximation was utilized. Within this framework, the electron wave vector (k_i) inside a rectangular potential barrier of height V_i is given by Equation S15, where E represents the kinetic energy of the electron, m^* denotes the electron effective mass (assumed to be $4.1m_0$ for hematite's conduction band minimum),[39] and $\hbar$ is the reduced Planck constant. The corresponding transmission coefficient (T_i) through the barrier of width a (set to 2.5 Å) was then determined using Equation S16. Here, the index i distinguish

between the pristine ($i=0$) and chemically modified ($i=1$) grain boundary conditions.

$$k_i = \sqrt{[2m^*(V_i - E)]}/\hbar \quad \text{(Equation S15)}$$

$$T_i = [1 + (V_i^2 sinh^2(k_i a))/(4E(V_i - E))]^{-1} \quad \text{(Equation S16)}$$

Acute toxicity assessment

A toxicity assessment was performed using daphnids to evaluate the environmental safety of the wastewater generated in the photoelectrochemical cell, ensuring that its discharge poses no threat to aquatic organisms. Daphnids were maintained under standardized conditions (20 °C ± 2 °C; pH 7.0-7.6; and 16:8 h light-dark cycle) in biological incubators, following the parameters established by ABNT NBR 12723:2022.[40] For the ecotoxicity assays, neonates (< 24 h old) were randomly selected and exposed to zirconium (IV) oxynitrate hydrate, iron (III) nitrate nonahydrate, and aluminum nitrate nonahydrate, that were prepared in ultrapure water and subsequently diluted in reconstituted water ($NaHCO_3$ 96 mg L^{-1}, $CaSO_4.2H_2O$ 60 mg L^{-1}, $MgSO_4.7H_2O$ 123 mg L^{-1}, and KCl 4 mg L^{-1}).[40] Zr^{4+} was tested at 0.025; 0.05; 0.1 and 0.2 mg L^{-1}; Al^{3+} at 0.25; 0.5; 1.5; 3.0; 6.0 and 9.0 mg L^{-1}; Fe^{3+} at 0.5; 1.0; 2.0; 4.0; 8.0; 16.0 and 20.0 mg L^{-1}. The pH of the final test solutions was adjusted to 7.0-7.4 using 10 M NaOH, a range considered optimal for *Daphnia* survival. Each treatment was performed with six replicates, each containing five neonates (6-24 h old) exposed to 10 mL of test solution in a 6-well plate. Daphnids were exposed for 48 h, and the toxicity endpoint measured was immobility (inability to swim after gentle agitation of the plate). Negative controls (Daphnids exposed only to 10 mL of reconstituted water) were also evaluated for validity. Mortality of non-exposed organisms (negative controls) did not exceed 10%, in accordance with the validity criteria established by ABNT NBR 12723:2022. Experiments that did not meet this criteria were disregarded. These assays were repeated at least four times. The effective dose that immobilizes 50% of exposed organisms (EC_{50}) was calculated by Probit analysis and dose-response curves were obtained using GraphPad prism software.

3. REFERENCES

[1] Dimesso, L. Pechini Processes: An Alternate Approach of the Sol-Gel Method, Preparation, Properties, and Applications. In: *Handbook of Sol-Gel Science and Technology: Processing, Characterization and Applications;* Klein, L.; Aparicio, M.; Jitianu, A.; Springer, Cham., 2018, pp 1-22.

[2] Muche, D. N. F.; dos Santos, T. M. G.; Leite, G. P.; Melo Jr., M. A.; Gonçalves, R. V.; Souza, F. L. Tailoring hematite/FTO interfaces: New horizons for spin-coated hematite photoanodes targeting water splitting. *Mater. Lett.* **2019,** 254, 218-221.

[3] Kakihana, M.; Domen, K. The Synthesis of Photocatalysts Using the Polymerizable-Complex Method. *MRS Bull.* **2000,** 25, 27-31.

[4] Muche, D. N. F.; Carminati, S. A.; Nogueira, A. F.; Souza, F. L. Engineering interfacial modification on nanocrystalline hematite photoanodes: A close look into the efficiency parameters. *Sol. Energy Mater. Sol. Cells* **2020,** 208, 110377.

[5] Bedin, K. C.; Mouriño, B.; Rodríguez-Gutiérrez, I.; Souza Jr., J. B.; dos Santos, G. T.; Bettini, J.; Costa, C. A. R.; Vayssieres, L.; Souza, F. L. Solution chemistry back-contact FTO/hematite interface engineering for efficient photocatalytic water oxidation. *Chin. J. Catal.* **2022,** 43, 1247-1257.

[6] Péres, L. O.; Rodrigues, R. R.; Louarn, G. The Influence of Alkali Metals on the Doping of Poly(p-phenylene) Oligomers. *Molecules* **2022,** 27, 8699.

[7] Dubois, M.; Naji, A.; Billaud, D. Electrochemical insertion of alkaline ions into polyparaphenylene: effect of the crystalline structure of the host material. *Electrochim. Acta* **2001,** 46, 4301-4307.

[8] Tsamopoulos, A. J.; Wang, Z.-G. Ion Conductivity in Salt-Doped Polymers: Combined Effects of Temperature and Salt Concentration. *ACS Macro Lett.* **2024,** 13, 322-327.

[9] Verissimo, N. C.; Pires, F. A.; Rodríguez-Gutiérrez, I.; Bettini, J.; Fiuza, T. E. R.; Biffe, C. A.; Montoro, F. E.; Schleder, G. R.; Castro, R. H. R.; Leite, E. R.; Souza, F. L. Dual modification on hematite to minimize

small polaron effects and charge recombination for sustainable solar water splitting. *J. Mater. Chem. A* **2024,** 12, 6280-6293.

[10] Chaule, S.; Kang, J.; Ghule, B. G.; Kim, H.; Jang, J.-H. An Approach to Enhance PEC Water Splitting Performance through Al:Ti Codoping in Hematite (α-Fe_2O_3) Photoanode: The Effect of Al^{3+} as a Codopant. *ACS Mater. Lett.* **2024,** 6, 2897-2904.

[11] Koh, T. S.; Anushkkaran, P.; Hwang, J. B.; Choi, S. H.; Chae, W.-S.; Lee, H. H.; Jang, J. S. Magnetron Sputtered Al Co-Doped with Zr-Fe_2O_3 Photoanode with Fortuitous Al_2O_3 Passivation Layer to Lower the Onset Potential for Photoelectrochemical Solar Water Splitting. *Catalysis* **2022,** 12, 1467.

[12] Koh, T. S.; Anushkkaran, P.; Mahadik, M. A.; Chae, W.-S.; Lee, H. H.; Choi, S. H.; Jang, J. S. Kinetic study of the enhanced photoelectrochemical properties of microwave-assisted Al and Si co-doped Zr-Fe_2O_3 photoanodes. *Appl. Surf. Sci.* **2024,** 642, 158615.

[13] Pires, F. A.; Santos, G. T.; Bettini, J.; Costa, C. A. R.; Gonçalves, R. V.; Castro, R. H. R.; Souza, F. L. Selective placement of modifiers on hematite thin films for solar water splitting. *Sustainable Energy Fuels* **2023,** 7, 5005-5017.

[14] Jeong, I. K.; Mahadik, M. A.; Hwang, J. B.; Chae, W.-S.; Choi, S. H.; Jang, J. S. Lowering the onset potential of Zr-doped hematite nanocoral photoanodes by Al co-doping and surface modification with electrodeposited Co-Pi. *J. Colloid Interface Sci.* **2021,** 581, 751-763.

[15] Anushkkaran, P.; Mahadik, M. A.; Chae, W.-S.; Lee, H. H.; Choi, S. H.; Jang, J. S. Microwave-assisted sequential Pt/Al attachment on FeOOH for fabrication of highly efficient hematite photoanodes: Synergistic effect of Pt/Al co-doping and Al_2O_3 passivation layer. *Appl. Surf. Sci.* **2023,** 623, 157035.

[16] Long, X.; Wei, J.; Gao, L.; Shen, C.; Gao, Q.; Zhao, X. Collaborative role of co-doping and surface modifying for adjusting surface state of hematite to release the photoelectrochemical activity. *J. Alloys Compd.* **2024,** 991, 174235.

[17] Subramanian, A.; Mahadik, M. A.; Park, J.-W.; Jeong, I. K.; Chung, H.-S.; Lee, H. H.; Choi, S. H.; Chae, W.-S.; Jang, J. S. An effective strategy to promote hematite photoanode at low voltage bias via Zr^{4+}/Al^{3+} codoping and CoO_x OER co-catalysis. *Electrochim. Acta* **2019,** 319, 444-455.

[18] Hwang, J. B.; Dhandole, L. K.; Anushkkaran, P.; Chae, W.-S.; Choi, S. H.; Lee, H. H.; Jang, J. S. Microwave-assisted surface attachment of aluminum ions on *in situ* diluted titanium-doped hematite photoanodes for efficient photoelectrochemical water-splitting. *Sustainable Energy Fuels* **2022,** 6, 3056-3067.

[19] Fu, Z.; Jiang, T.; Zhang, L.; Liu, B.; Wang, D.; Wang, L.; Xie, T. Surface treatment with Al^{3+} on a Ti-doped α-Fe_2O_3 nanorod array photoanode for efficient photoelectrochemical water splitting. *J. Mater. Chem. A* **2014,** 2, 13705-13712.

[20] Kleiman-Shwarsctein, A.; Huda, M. N.; Walsh, A.; Yan, Y.; Stucky, G. D.; Hu, Y.-S.; Al-Jassim, M. M.; McFarland, E. W. Electrodeposited Aluminum-Doped α-Fe_2O_3 Photoelectrodes: Experiment and Theory. *Chem. Mater.* **2010,** 22, 510-517.

[21] Reddy, C. V.; Reddy, I. N.; Akkinepally, B.; Reddy, K. R.; Shim, J. Synthesis and photoelectrochemical water oxidation of (Y, Cu) codoped α-Fe_2O_3 nanostructure photoanode. *J. Alloys Compd.* **2020,** 814, 152349.

[22] Wang, K.; Liu, B.; Liu, Z.; Jiang, X.; Zhang, Z.; Han, W. Improved photocarrier separation in Ga/Fe gradient $(Ga, Fe)_2O_3$ thin films. *Appl. Phys. Lett.* **2023,** 122, 203901.

[23] Furushima, R.; Tanaka, S.; Kato, Z.; Uematsu, K. Orientation distribution-Lotgering factor relationship in a polycrystalline material – as an example of bismuth titanate prepared by a magnetic field. *J. Ceram. Soc. Jpn.* **2010,** 118, 921-926.

[24] Muniz, F. T. L.; Miranda, M. A. R.; Santos, C. M.; Sasaki, J. M. The Scherrer equation and the dynamical theory of X-ray diffraction. *Acta Cryst.* **2016,** A72, 385-390.

[25] Makuła, P.; Pacia, M.; Macyk. W. How to Correctly Determine the Band Gap Energy of Modified Semiconductor Photocatalysts Based on UV–Vis Spectra. *J. Phys. Chem. Lett.* **2018,** 9, 6814-6817.

[26] Baddorf, A. P. Identifying the secondary electron cutoff in ultraviolet photoemission spectra for work function measurements of non-ideal surfaces. *Sci. Rep.* **2023,** 13, 13452.

[27] Dotan, H.; Sivula, K.; Grätzel, M.; Rothschild, A.; Warren, S. C. Probing the photoelectrochemical properties of hematite (α-Fe_2O_3) electrodes using hydrogen peroxide as a hole scavenger. *Energy Environ. Sci.* **2011,** 4, 958-964.

[28] Zandi, O.; Hamann, T. W. The potential *versus* current state of water splitting with hematite. *Phys. Chem. Chem. Phys.* **2015,** 17, 22485-22503.

[29] Jung, S.; McCrory, C. C. L.; Ferrer, I. M.; Peters, J. C.; Jaramillo, T. F. Benchmarking nanoparticulate metal oxide electrocatalysts for the alkaline water oxidation reaction. *J. Mater. Chem. A.* **2016,** 4, 3068-3076.

[30] McCrory, C. C. L.; Jung, S.; Peters, J. C.; Jaramillo, T. F. Benchmarking Heterogeneous Electrocatalysts for the Oxygen Evolution Reaction. *J. Am. Chem. Soc.* **2013,** 135, 16977-16987.

[31] Rodríguez-Gutiérrez, I.; Djatoubai, E.; Su, J.; Veja-Poot, A.; Rodríguez-Gattorno, G.; Souza, F. L.; Oskam, G. An intensity-modulated photocurrent spectroscopy study of the charge carrier dynamics of WO_3/$BiVO_4$ heterojunction systems. *Sol. Energy Mater. Sol. Cells* **2020,** 208, 110378.

[32] Sivula, K. Mott-Schottky Analysis of Photoelectrodes: Sanity Checks are Needed. *ACS Energy Lett.* **2021,** 6, 2549-2551.

[33] Dotan, H.; Mathews, N.; Hisatomi, T.; Gratzel, M.; Rothschild, A. On the Solar to Hydrogen Conversion Efficiency of Photoelectrodes for Water Splitting. *J. Phys. Chem. Lett.* **2014,** 5, 3330-3334.

[34] Perdew, J. P.; Burke, K.; Ernzerhof, M. Generalized gradient approximation made simple. *Phys. Rev. Lett.* **1996,** 77, 3865–3868.

[35] Blöchl, P. E. Projector augmented-wave method. *Phys. Rev. B* **1994,** 50, 17953–17979.

[36] Monkhorst, H. J.; Pack, J. D. Special points for Brillouin-zone integrations. *Phys. Rev. B* **1976,** 13, 5188–5192.

[37] Dudarev, S. L.; Botton, G. A.; Savrasov, S. Y.; Humphreys, C. J.; Sutton, A. P. Electron-energy-loss spectra and the structural stability of nickel oxide: An LSDA + U study. *Phys. Rev. B* **1998,** 57, 1505–1509.

[38] Huang, X.; Ramadugu, S. K.; Mason, S. E. Surface-specific DFT + U approach applied to α-Fe_2O_3 (0001). *J. Phys. Chem. C* **2016,** 120, 4919–4930.

[39] De Lima, F. C.; Schleder, G. R.; Souza Junior, J. B.; Souza, F. L.; Destro, F. B.; Miwa, R. H.; Leite, E. R.; Fazzio, A. Unveiling the dopant segregation effect at hematite interfaces. *Appl. Phys. Lett.* **2021,** 118, 201602.

[40] Associação Brasileira de Normas Técnicas (ABNT). *Ecotoxicologia aquática — Toxicidade aguda — Método de ensaio com Daphnia spp (Crustacea, Cladocera).* ABNT NBR 12713:2022.
